# Composition-Defined Optical Properties and the Direct-to-Indirect Transition in Core–Shell In_1–*x*_Ga*_x_*P/ZnS Colloidal Quantum Dots

**DOI:** 10.1021/jacs.3c02709

**Published:** 2023-07-19

**Authors:** Aritrajit Gupta, Justin C. Ondry, Kailai Lin, Yunhua Chen, Margaret H. Hudson, Min Chen, Richard D. Schaller, Aaron J. Rossini, Eran Rabani, Dmitri V. Talapin

**Affiliations:** † Department of Chemistry, James Franck Institute, and Pritzker School of Molecular Engineering, University of Chicago, Chicago, Illinois 60637, United States; ‡ Center for Nanoscale Materials, Argonne National Laboratory, Argonne, Illinois 60439, United States; § Department of Chemistry, University of California, Berkeley, California 94720, United States; ∥ US DOE Ames Laboratory, Ames, Iowa 50011, United States; ⊥ Department of Chemistry, Iowa State University, Ames, Iowa 50011, United States; # Department of Chemistry, Northwestern University, Evanston, Illinois 60208, United States; ∇ Materials Sciences Division, Lawrence Berkeley National Laboratory, Berkeley, California 94720, United States; ○ The Raymond and Beverly Sackler Center of Computational Molecular and Materials Science, Tel Aviv University, Tel Aviv 69978, Israel

## Abstract

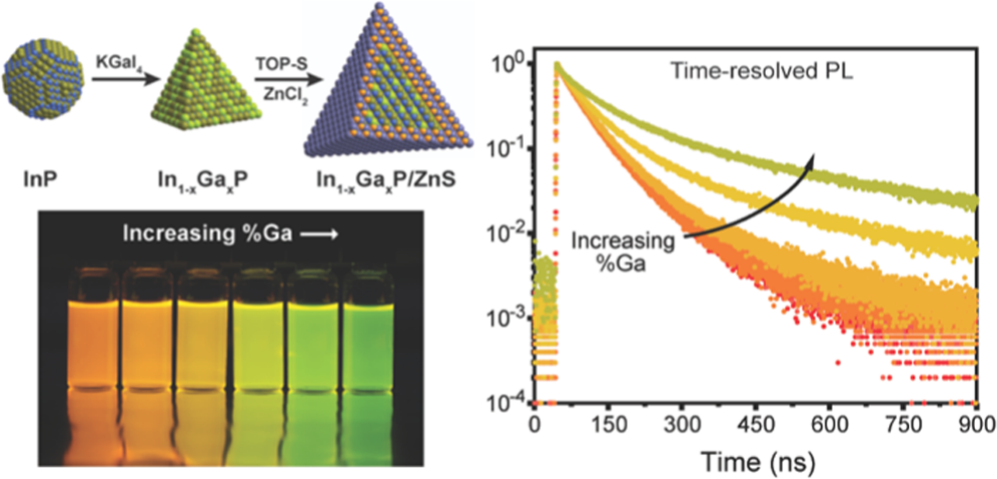

Semiconductors are commonly divided into materials with direct or indirect band gaps based on the relative positions of the top of the valence band and the bottom of the conduction band in crystal momentum (**k**) space. It has, however, been debated if **k** is a useful quantum number to describe the band structure in quantum-confined nanocrystalline systems, which blur the distinction between direct and indirect gap semiconductors. In bulk III–V semiconductor alloys like In_1–*x*_Ga*_x_*P, the band structure can be tuned continuously from the direct- to indirect-gap by changing the value of *x*. The effect of strong quantum confinement on the direct-to-indirect transition in this system has yet to be established because high-quality colloidal nanocrystal samples have remained inaccessible. Herein, we report one of the first systematic studies of ternary III–V nanocrystals by utilizing an optimized molten-salt In-to-Ga cation exchange protocol to yield bright In_1–*x*_Ga*_x_*P/ZnS core–shell particles with photoluminescence quantum yields exceeding 80%. We performed two-dimensional solid-state NMR studies to assess the alloy homogeneity and the extent of surface oxidation in In_1–*x*_Ga*_x_*P cores. The radiative decay lifetime for In_1–*x*_Ga*_x_*P/ZnS monotonically increases with higher gallium content. Transient absorption studies on In_1–*x*_Ga*_x_*P/ZnS nanocrystals demonstrate signatures of direct- and indirect-like behavior based on the presence or absence, respectively, of excitonic bleach features. Atomistic electronic structure calculations based on the semi-empirical pseudopotential model are used to calculate absorption spectra and radiative lifetimes and evaluate band-edge degeneracy; the resulting calculated electronic properties are consistent with experimental observations. By studying photoluminescence characteristics at elevated temperatures, we demonstrate that a reduced lattice mismatch at the III–V/II–VI core–shell interface can enhance the thermal stability of emission. These insights establish cation exchange in molten inorganic salts as a viable synthetic route to nontoxic, high-quality In_1–*x*_Ga*_x_*P/ZnS QD emitters with desirable optoelectronic properties.

## Introduction

Electronic and optoelectronic devices such as transistors, photodetectors, and light-emitting diodes (LEDs) employ high-quality semiconducting layers as the active material. In bulk semiconductors, electronic transitions must conserve both energy and momentum. This requirement divides semiconductors into two distinct families: direct-gap semiconductors, where the top of the valence band and bottom of the conduction band occur at the same point in the Brillouin zone (Γ point for typical II–VI materials like CdSe, and L points for typical IV–VI materials like PbS); and indirect-gap semiconductors, where this condition is not met.^1^ In sub-10 nm semiconductor nanocrystals, as the carrier wavefunctions are confined in dimensions smaller than the exciton Bohr radius, the energy gap between the highest-energy occupied and lowest-energy unoccupied states becomes a function of the particle size. The canonical examples of colloidal semiconductor nanostructures, such as CdSe nanocrystals, also known as quantum dots (QDs), show size-dependent absorption and emission spectra that are well described by particle-in-a-box models.^2^ Quantum-confined absorption and photoluminescence (PL) have been well documented in colloidal nanocrystals of elemental and compound semiconductors, alloys, and core–shell structures.^3−6^ The examples cited here are for compositions that, in their bulk form, are direct-band-gap semiconductors. Direct-gap semiconductors generally show strong dipole-allowed interband transitions and, correspondingly, quantum dots derived from direct-gap semiconductors are characterized by strong excitonic transitions,^7^ high luminescence efficiency with fast nanosecond radiative lifetimes,^8^ and state filling induced transient optical bleaching upon photoexcitation.^9,10^

In materials like silicon and germanium, which have an indirect band gap in the bulk, the effect of quantum confinement on absorption and photoluminescence is more complicated due to the momentum mismatch between the valence band maximum and conduction band minimum. In bulk crystals of these materials, absorption or emission of photons at the band gap energy requires phonons to compensate for this momentum mismatch, leading to weak band-edge absorption and very weak, often undetectable photoluminescence (PL). Despite this additional factor, Si and Ge nanocrystals show size-dependent absorption onsets^11^ and, in some cases, size-dependent PL.^12^ However, the absorption spectra do not exhibit distinct excitonic features, the PL has long μs lifetimes,^12^ and nanocrystals derived from indirect-gap materials do not display excitonic bleach signatures near the band edge.^13^ The finite size of the crystallite relaxes, at least partially, the requirement of conservation of momentum, and mixing of the Γ and X valleys may enable direct-like absorption and emission of photons without phonon-mediated processes.^14^ Indeed, low-temperature PL measurements on silicon nanocrystals show zero phonon emission for small particles, indicating that a direct (not phonon-mediated) emission pathway is possible.^15^ However, the mixing of the states is very weak, and silicon remains essentially an indirect band gap material in nanocrystalline form.^9^

**Figure 1:**
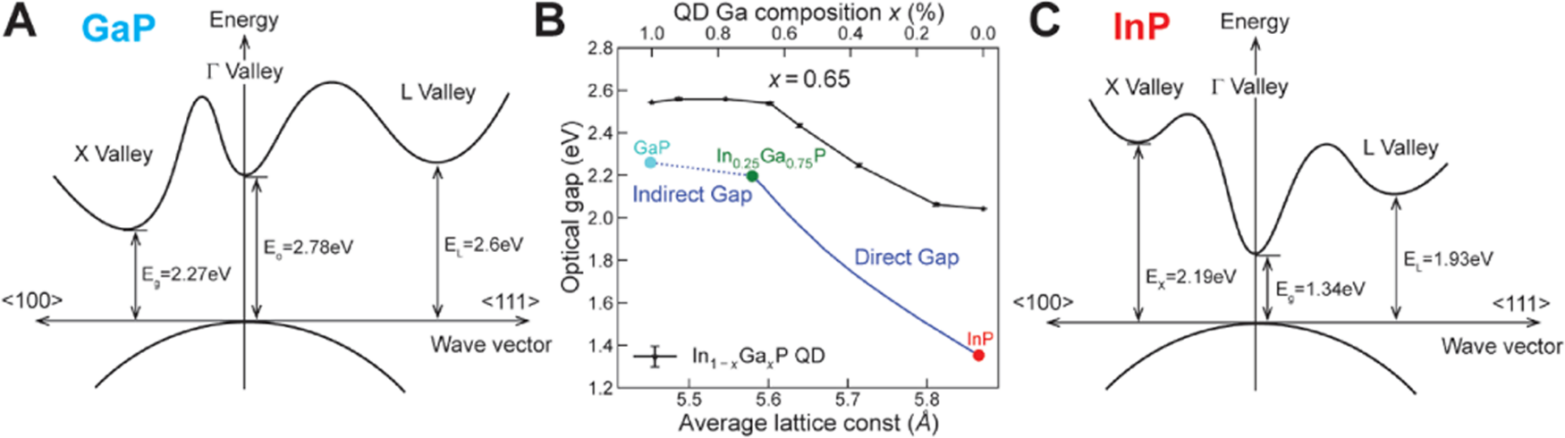
Band structure of bulk (A) GaP and (C) InP. (B) Composition dependence of band gap and lattice constant in bulk alloyed In_1–*x*_Ga*_x_*P crystals at 300 K (blue line) from previously reported data in refs (35) and (36). Theoretical predictions for the composition dependence of the band gap for 4 nm diameter In_1–*x*_Ga*_x_*P nanocrystals (black squares) calculated using a semi-empirical pseudopotential model described below and in the Supporting Information (SI) (Figures S1–S3 and Tables S1 and S2). Panel (A) was adapted with permission from ref (16) (Copyright 1971 American Physical Society). Panel (C) was adapted with permission from ref (18) (Copyright 1986 American Physical Society).

The two cases thus far discussed represent distinct material classes that cannot be continuously tuned between direct- and indirect-behaving systems. One rather unique system where the indirect/direct nature of the transition is continuously tunable is InP–GaP.^16^ InP is a direct-band-gap semiconductor with a valence band maximum and a conduction band minimum at the Γ point (Figure 1C).^17,18^ GaP is an indirect-band-gap semiconductor with a valence band maximum at the Γ point and a conduction band minimum at the X point^18^ (Figure 1A). These two materials form solid solutions at all compositions: alloying increases the energy of the Γ point conduction band minimum and lowers the energy of the X point conduction band minimum. At a bulk composition of ∼75% gallium, the Γ and X points of the conduction band cross in energy, resulting in a change in the slope of the band gap vs composition relationship (Figure 1B), and In_1–*x*_Ga*_x_*P exhibits indirect behavior at higher gallium contents.^19^

The effect of quantum confinement on the In_1–*x*_Ga*_x_*P QDs as a function of size and *x*-value has not been systematically studied. In particular, it is not clear how quantum confinement affects the nature of the composition-determined direct-to-indirect transition. For Stranski–Krastanov (SK) grown epitaxial QDs, since the strain is the driving force for island formation, it is difficult to synthesize materials with independent control over size and composition.^20^ Moreover, SK QDs are typically larger than the Bohr exciton radius and thus lie within the weak confinement regime, minimizing any potential Γ–X mixing, which may occur due to strong quantum confinement. Quantum-confined nanowire or nanorod structures grown *via* epitaxial techniques or solution techniques have more flexibility in tuning size and composition independently, but achievable dimensions are still too large to achieve strong quantum confinement in this material system.^21−23^

Solution phase colloidal synthesis is an ideal method for preparing small semiconductor crystallites with strong quantum confinement due to precisely controllable nucleation and growth kinetics. These methods have been well developed for II–VI, IV–VI, and In–Pn (Pn = P, As, Sb) materials. However, due to the high reactivity of gallium toward organic solvents at high temperatures,^24^ the synthesis of high-quality Ga–Pn and In_1–*x*_Ga*_x_*Pn materials is not well developed. Currently, there are many established routes to high-quality InP^25−28^ with control over size and morphology, which result in bright emissive semiconductors, while only a few routes to colloidal GaP^29,30^ have been reported. There are a select few examples of In_1–*x*_Ga*_x_*P particles that have been prepared via colloidal growth methods. However, based on reported data, the uniform incorporation of gallium into the InP lattice has not been unambiguously verified.^31,32^ The lack of general control over composition and size in these systems limits their utility for studying the fundamentals of the direct-to-indirect transition in quantum-confined In_1–*x*_Ga*_x_*P.

Recently, our group has developed methods to prepare stable colloids in molten-salt solvents^33,34^ and used these solvents as a high-temperature reaction medium for preparing ternary In_1–*x*_Ga*_x_*P and In_1–*x*_Ga*_x_*As colloidal nanocrystals.^35−37^ In this synthetic approach, we start with InP or InAs nanocrystals, where we can leverage their well-developed size control methods and disperse the nanocrystals in a gallium-containing molten-salt solvent. High-temperature annealing of the InP/molten-salt mixture causes solid-state diffusion of gallium into the InP lattice with coupled out-diffusion of the In, leading to In_1–*x*_Ga*_x_*P nanocrystals. The temperature and duration of annealing can be used to control the degree of gallium incorporation independent of particle size. The high-temperature stability and rigorously oxygen-free environment enabled by the molten salts further enable the study of the In_1–*x*_Ga*_x_*P without oxidation from the decomposition of oxygen-containing ligands or atmospheric contamination.

In this work, we synthesize a series of In_1–*x*_Ga*_x_*P nanocrystals where we vary the diameter from 3.2 to 4.9 nm and *x*-value from 0 to 0.92. We use solid-state NMR to validate that the molten-salt-derived nanocrystals have minimal surface oxidation and that In and Ga are alloyed uniformly within the nanocrystals rather than forming a distinct core–shell morphology. To fully exploit the oxygen-free nature of the molten-salt-derived In_1–*x*_Ga*_x_*P nanocrystal surface, we develop a ZnS shelling protocol that rigorously avoids oxygen or oxygen-containing precursors. We show that the resulting In_1–*x*_Ga*_x_*P/ZnS colloidal nanocrystals in the *x* = 0.14–0.57 range can display PL quantum yields (PLQY) as high as 89% with PL line widths of 57 nm full width at half-maximum (FWHM). Optoelectronic characterization *via* time-resolved PL (TRPL) and transient absorption (TA) spectroscopy on the resulting In_1–*x*_Ga*_x_*P/ZnS nanocrystals suggests that the oscillator strength of the emissive state decreases as the gallium content is increased. We further performed theoretical calculations via atomistic semi-empirical pseudopotential methods to capture the electronic structure of the alloyed QD systems. Calculated optoelectronic properties, including optical gaps, absorption spectra, and radiative lifetimes, show good agreement with experimental measurements, capturing the trend as gallium content increases in alloyed In_1–*x*_Ga*_x_*P QDs. Fluence-dependent transient absorption shows that the Auger recombination rate is not strongly modulated by composition; rather, well-established volume scaling dominates multiexciton physics in these quantum-confined nanocrystals. In In_1–*x*_Ga*_x_*P/ZnS samples with *x* = 0.65–0.92, we show that the excitonic bleach feature disappears for samples that contain >65% gallium, consistent with the conversion of In_1–*x*_Ga*_x_*P/ZnS to an indirect band gap material. Calculated electronic structures of the conduction band-edge states show the Γ- to X-valley transition as gallium composition increases and help decipher the direct-to-indirect band gap transition in the confined system. Finally, we show that In_1–*x*_Ga*_x_*P/ZnS nanocrystals display better thermal stability relative to InP/ZnS core-shells, likely due to a smaller mismatch of the lattice constants between the In_1–*x*_Ga*_x_*P core and ZnS shell. Together, our results provide insight into the nature of the composition-driven direct-to-indirect transition of a model semiconductor system, providing crucial insight for designing next-generation colloidal QD materials.

## Results and Discussion

### Synthesis of In_1–*x*_Ga*_x_*P Colloidal Quantum Dots and In_1–*x*_Ga*_x_*P/ZnS Core–Shell Nanocrystals

An In-to-Ga cation exchange reaction performed on indium pnictide nanocrystals dispersed in molten salts offers a viable route toward synthesizing the otherwise elusive gallium-containing ternary III–V phases.^35,37^ We exchange the native long-chain surfactants on the as-synthesized nanocrystals with compact charged inorganic ligands in order to disperse them in molten-salt media. Z-type gallium halide ligands constitute a unique candidate for this role, as they avoid introducing additional heteroatom impurities such as chalcogenides.^36,37^ Gallium is notoriously oxophilic at elevated temperatures, necessitating that oxygen-containing moieties are avoided at all subsequent steps. We, therefore, introduce an amine-based recovery in this report, allowing us to obtain a colloidal solution of alloyed ternary In_1–*x*_Ga*_x_*P nanocrystals without using carboxylate ligands. This further prevents surface oxidation in the subsequent high-temperature ZnS shelling step.

**Figure 2:**
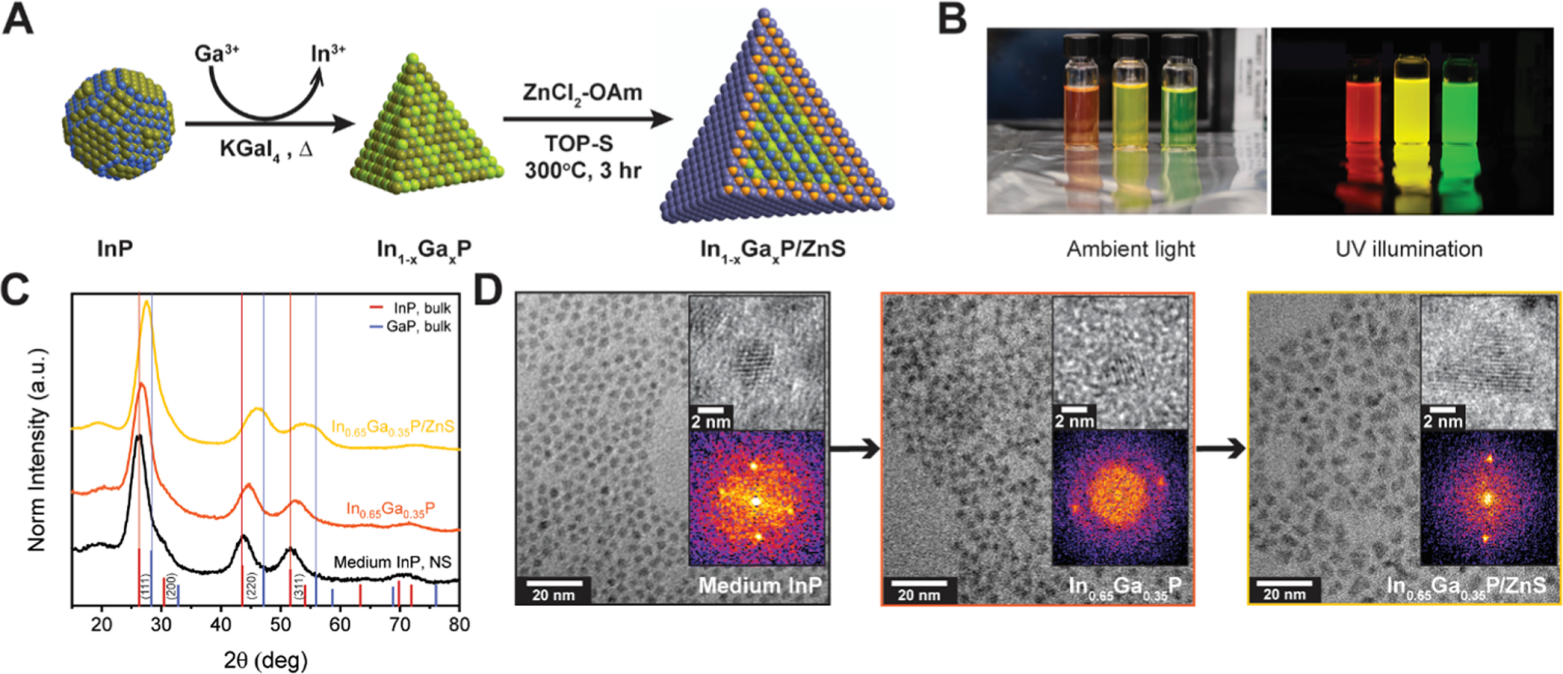
(A) Reaction scheme describing the conditions for the In-to-Ga cation exchange and the subsequent ZnS shelling steps. The photographs in (B) are of emissive core–shell In_1–*x*_Ga*_x_*P/ZnS samples with similar gallium contents synthesized from 4.9 nm InP (red emitting post cation exchange), 4.0 nm InP (yellow), and 3.2 nm InP (green) populations. (C) Representative powder X-ray diffraction (XRD) patterns indicate a zinc-blende crystal structure for the cores as well as for the core–shell samples derived from the 4.0 nm InP particles. (D) Transmission electron microscopy (TEM) images indicate the evolution of particle morphology over the cation exchange and shelling steps.

To establish the general applicability of our protocol, we focus on three kinds of colloidal InP nanocrystals with different sizes and surface terminations. Trioctylphosphine (TOP)/trioctylphosphine oxide (TOPO) passivated InP nanocrystals were prepared by a dehalosilylation reaction between InCl_3_ and tris(trimethylsilyl)phosphine, following an established synthesis pioneered by Micic et al.^30,38^ A population with an average diameter of ∼4.9 nm was size-selectively separated from the reaction mixture. We also synthesized small InP nanocrystals of ∼3.2 nm diameter via an adaptation of the indium myristate-based recipe pioneered by the Peng group.^25^ A third intermediate-sized InP population of 4.0 nm diameter, which is similar to QD cores used for display applications, was obtained from a proprietary source. The size distributions were calculated from maximum-entropy fits to the corresponding small angle X-ray scattering (SAXS) patterns, Figure S4A–F.^39^ Following previous reports, the native organic ligands on each of these populations were exchanged for inorganic Z-type GaI_3_ ligands.^36,37^ We observe only small differences between the absorption spectra recorded using stable colloidal dispersions of these inorganically capped particles before and after ligand exchange (Figure S5), which we ascribe to subtle changes in energy levels of the band structure, influenced by changes in the QD surface chemistry.^40^ The InP particles capped with GaI_3_ ligands were added to a KGaI_4_ salt matrix (Figure 2A), flame-sealed inside quartz ampoules under vacuum, and subjected to different annealing conditions to yield similar gallium compositions, as noted in Table S3. Larger nanocrystals are more resistant to cation exchange than their smaller counterparts under similar reaction conditions, thereby requiring higher annealing temperatures for similar final gallium contents. Next, the salt matrix was dissolved using anhydrous acetonitrile, and the nanocrystals, which are insoluble in acetonitrile, were recovered via centrifugation. We used a mixture of dodecylamine and octadecylamine as entropic ligands to confer colloidal stability to the In_1–*x*_Ga*_x_*P nanocrystals in toluene.^41^ Excess amine ligands were removed by precipitating the particles using anhydrous methyl acetate and redispersing them in a desired nonpolar solvent. We performed X-ray diffraction studies on the cation-exchanged products recovered with the aforementioned alkylamine ligands to ensure that there was no decomposition of the zinc-blende phase (Figure S6). We estimated lattice constants from the recorded diffraction patterns using Le Bail refinement, which allowed us to assign a resultant gallium composition to the alloyed In_1–*x*_Ga*_x_*P phases. Additional peaks appearing before the (111) peak (*i.e.*, at lower 2θ) are more pronounced in the smaller populations and likely stem from the ordering of organic capping ligands.^42^

To study the photophysics of In_1–*x*_Ga*_x_*P as a function of composition, we passivate the surface to remove surface trap states. Since gallium has a high affinity toward oxygen at elevated temperatures, exposure of the alloyed In_1–*x*_Ga*_x_*P nanocrystals to oxygen-containing shelling precursors (*e.g.*, zinc carboxylates) can potentially trigger surface oxidation during zinc sulfide shell growth. Thus, we introduce a shelling protocol that allows us to grow a wide band gap ZnS shell on the In_1–*x*_Ga*_x_*P nanocrystals in a reducing environment (Figure 2A) without oxygen-containing precursors. ZnCl_2_ in oleylamine and trioctylphosphine sulfide (TOP-S) were used as the zinc and sulfur precursors, respectively (see the Supporting Information for the detailed reaction scheme). The In-to-Ga cation exchange decreases the lattice constant of the nanocrystals relative to the initial InP. This provides a better lattice match with wide band gap shelling materials such as ZnS, thus yielding highly emissive core–shell In_1–*x*_Ga*_x_*P/ZnS QDs. We obtain red, yellow, and green emissions from the large-, medium-, and small-core populations, respectively (Figure 2B), demonstrating the expected effects of quantum confinement to tune the band gap of these alloyed nanocrystals as a function of size while retaining a similar composition at *x* ∼ 35% Ga content. Steady-state absorption and emission spectra of the individual core–shell samples are plotted in Figure S7, along with the absorption spectra of the relevant cores. In_0.65_Ga_0.35_P/ZnS nanocrystals synthesized from the 4 nm InP QDs have a 60 nm PL FWHM with a high PLQY of 75%, measured relative to the reference organic dye coumarin 153. Prior studies indicated that emission in the ∼530 nm region can be achieved with very small InP nanocrystals (∼2 nm diameter) that are inherently less stable.^26^ Notably, we could achieve green emission with a relatively high PLQY of ∼48% using In_0.66_Ga_0.34_P/ZnS QDs derived from the 3.2 nm InP population without any additional optimization. For larger 4.9 nm diameter In_0.62_Ga_0.38_P/ZnS QDs, we achieved a PLQY of ∼19%. We have previously shown that particles derived from this method have a higher density of internal defects,^37^ indicating that starting with highly crystalline InP particles is likely key to achieving good-quality In_1–*x*_Ga*_x_*P emitters. Seeking to compare the contribution of population dispersity toward emission broadening in these samples, we constructed excitation–emission maps (Figure S8A,C,E) and found that a broad emission is inherent for the large particles, perhaps resulting from their higher defect concentration. The impressive emission properties of the In_1–*x*_Ga*_x_*P/ZnS samples reported in this study suggest that the ZnS shell we have grown provides efficient surface passivation, even though the lattice mismatch of In_0.65_Ga_0.35_P with the ZnS shell is ∼5.5%. In the 4 nm population, this is evidenced by the diffraction peaks getting narrower and further shifting to higher 2θ values after shelling (Figure 2C). Transmission electron microscopy (TEM) images indicate that the In-to-Ga cation exchange reaction in a molten gallium halide environment leads to increased faceting of the nanocrystals (Figure 2D), consistent with our previous observations.^37^ We estimate that two monolayers of ZnS are grown under present shelling conditions, resulting in an average tetrahedral edge length of 9 nm for the representative faceted In_0.65_Ga_0.35_P/ZnS nanocrystals derived from 4 nm InP (Figure 2D). The reduced lattice mismatch may still be too large, leading to somewhat irregular growth of the ZnS shell at our reaction temperatures. While this proves sufficient to provide reasonably high quantum yields, pursuing lattice-matched heterostructures or graded alloy shells may enable the growth of thicker shells and more efficient surface passivation.

### Solid-State Nuclear Magnetic Resonance (ssNMR) Spectroscopy of InP and In_1–*x*_Ga*_x_*P Nanocrystals

The transformation of InP into In_1–*x*_Ga*_x_*P *via* partial cation exchange reaction is expected to yield a homogeneous alloying of Ga and In throughout the QD volume, but alternative scenarios, such as graded alloying or even phase separation, cannot be ruled out *a priori*, and previous XRD simulations suggested that this technique alone is relatively insensitive to these structural parameters.^37^ Moreover, as discussed above, the InP and In_1–*x*_Ga*_x_*P phases are susceptible to oxidation. Previous studies have shown that synthesizing InP nanocrystals from oxygen-containing indium carboxylate precursors can result in substantial surface oxidation,^36^ conventionally estimated as the fraction of total phosphorus that is present in the form of various oxidized phosphorus species.^43−45^ Surface oxidation of InP and In_1–*x*_Ga*_x_*P QDs can also be introduced during post-synthetic handling and cation exchange in the molten-salt medium. In this work, to elucidate the chemical environment of P, Ga, and In, we use a combination of ssNMR techniques to probe the structure of our QD materials. The TOP/TOPO passivated 4.9 nm InP nanocrystals likely have minimal surface oxidation, to begin with,^46^ and therefore constitute a convenient system for probing surface oxidation with ssNMR after subsequent transformations. The native organic ligands were exchanged with GaI_3_, and the particles were dispersed in a molten KGaI_4_ salt matrix and annealed at 440 °C for 4 h to yield alloyed In_1–*x*_Ga*_x_*P nanocrystals. Expectedly, we observe a blue shift of the excitonic feature in the absorption spectra after cation exchange (Figure S9A) and estimate a gallium composition of 50% from powder X-ray diffraction (Figure S9B). Purified nanocrystals were dried as a pellet for use in ssNMR experiments.

**Figure 3:**
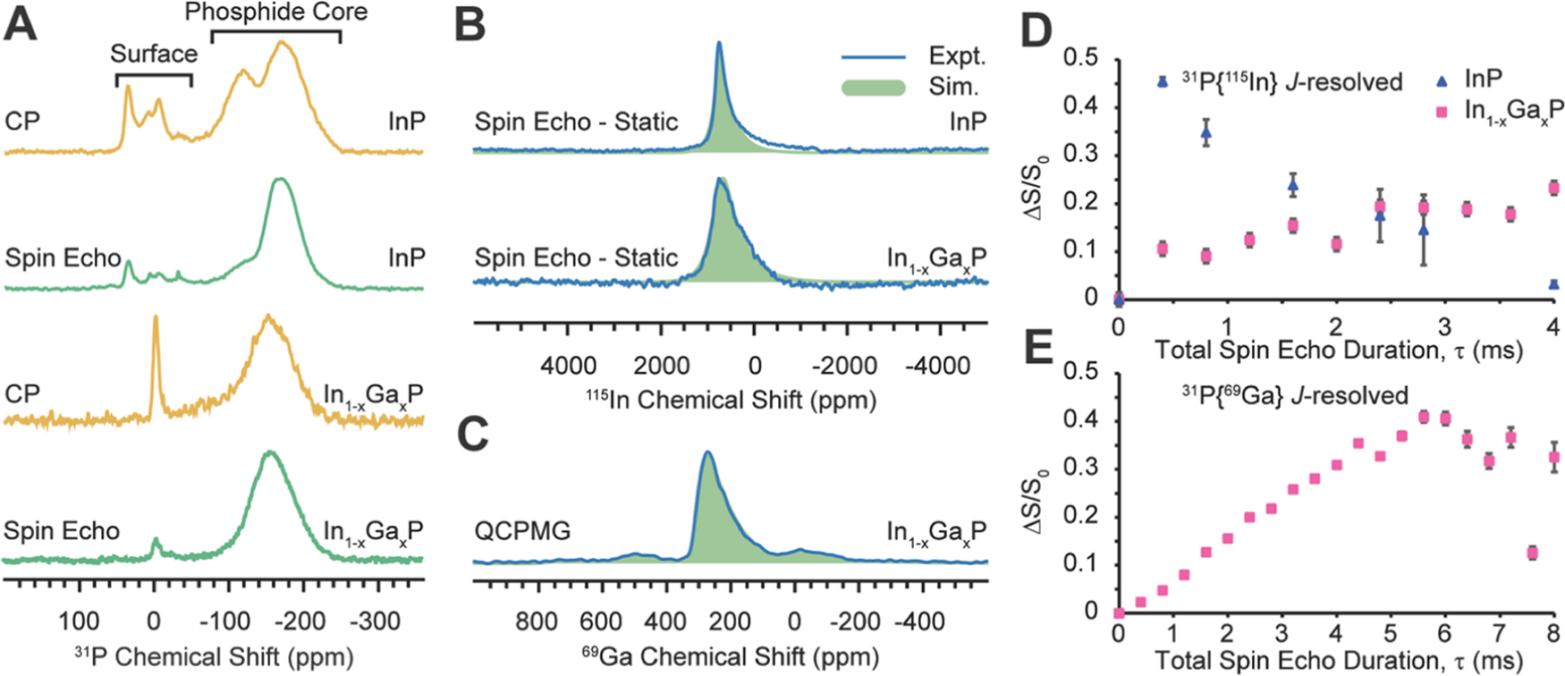
(A) 25 kHz MAS ^31^P spin-echo and ^1^H → ^31^P CP solid-state NMR spectra of TOP/TOPO-capped InP and octadecylamine/dodecylamine-capped In_0.5_Ga_0.5_P QDs. (B) Static ^115^In spin-echo NMR spectra of InP and In_0.5_Ga_0.5_P QDs. (C) The 25 kHz MAS ^69^Ga quadrupolar Carr–Purcell Meiboom–Gill (QCPMG) spectrum of In_0.5_Ga_0.5_P QDs. (B, C) Both ^115^In and ^69^Ga NMR spectra are fitted with a Czjzek distribution of quadrupolar interactions, the parameters for which are indicated in the Supporting Information. (D, E) ^31^P{^115^In} and ^31^P{^69^Ga} *J*-resolved NMR experiments. The signal dephasing as a function of the spin-echo duration (τ) is shown for the phosphide ^31^P NMR signal of InP (blue triangles) and In_0.5_Ga_0.5_P (pink squares). ^1^H → ^31^P cross-polarization was used to enhance the ^31^P NMR signals. The error bars were determined by measuring the signal-to-noise of the control spectra. One standard deviation in the signal intensities (σ) was assumed to be equal to the signal-to-noise multiplied by the signal dephasing.

For TOP/TOPO-capped InP nanocrystals and our amine-capped In_0.50_Ga_0.50_P nanocrystals, we employed ^31^P direct excitation (spin echo) and surface-selective ^1^H → ^31^P cross-polarization (CP) ssNMR spectroscopy. These experiments were performed to identify potential binding sites for trioctylphosphine (TOP), trioctylphosphine oxide (TOPO), or dodecylamine (DDA) and octadecylamine (ODA) on the nanocrystal surfaces and to differentiate bulk and surface P sites (Figure 3A). The ^31^P spin-echo NMR spectra are expected to show signals from all P species present in the sample, including surface and core phosphide sites, while the ^1^H → ^31^P CP experiment will enhance NMR signals from ^31^P spins nearer to the surface of the material, which are dipole coupled to ^1^H spins (*i.e.*, ^1^H and ^31^P spins, which are within *ca.* 5 Å of one another). The intense ^31^P NMR signal centered at −164 ppm is attributed to P atoms residing in the phosphide core of InP and In_0.50_Ga_0.50_P because this peak has the highest intensity in the spin-echo spectrum, and a similar chemical shift was reported in prior studies of InP and In_1–*x*_Ga*_x_*P phases.^46−51^ There is one additional ^31^P NMR signal at *ca.* −119 ppm observed in the spectrum of the InP QDs next to the most intense ^31^P NMR signal, which is assigned to subsurface phosphide ^31^P atoms.^46^ Since only a small fraction of the atoms constitute the surface, the chemical shifts of low-intensity ^31^P NMR signals in the range of +50 to −50 ppm are assigned to the surface P sites. Based on prior assignments, the ^31^P NMR signals at *ca.* 35, 6, −7, and −32 ppm correspond to phosphonate, phosphate, poly/pyrophosphate, and TOP, respectively.^46,51−54^ The residual oxidation is likely caused by oxygen-containing organic ligands, post-synthetic treatments such as non-solvent wash, or minor surface oxidation during sample transfer. We can estimate the extent of InP oxidation by comparing the areas of resonance assigned to InP and various oxidized InPO*_y_* (*y* = 2–4) species in the spin-echo spectra. Evidently, the extent of oxidation is negligible and largely localized to the nanocrystal surface. For reference, previous NMR studies of InP QDs showed a significantly higher degree of surface oxidation.^44,55,56^

Static ^115^In spin-echo spectra of InP and In_0.50_Ga_0.50_P QDs are displayed in Figure 3B. Both spectra have a slightly asymmetric flank to the low-frequency side. ^115^In is a quadrupolar (*I* = 9/2) nucleus with a sizeable quadrupolar moment. The asymmetric broadening is indicative of the presence of a distribution of quadrupolar coupling values.^57−60^ We note that the ^115^In NMR spectrum of bulk InP does not exhibit such asymmetric broadening because the highly symmetric zinc-blende lattice of InP results in negligible ^115^In quadrupolar interactions.^61^ However, in InP nanocrystals, the symmetry of the lattice will be disrupted in the regions that are proximate to the surface of the particles. Hence, quadrupolar broadening of the ^115^In NMR peaks is expected in all QD samples. The ^115^In NMR spectra were fit by considering a Czjzek distribution in the ^115^In quadrupolar coupling constant (*C*_Q_, see Table S4). Additional broadening of ^115^In NMR signals will also arise due to dipolar and scalar couplings to the ^31^P spins; however, this broadening is usually symmetric, so it was not accounted for in the simulations. The ^115^In NMR spectrum of the In_0.50_Ga_0.50_P QDs is broader than that of the InP QDs. Additional quadrupolar broadening is expected to arise if gallium is alloyed into the In_0.50_Ga_0.50_P QDs because there will now be a distribution in In–P bond lengths and angles that will depend upon the number of nearest In and Ga neighbors situated in the lattice,^50^ and this observed broadening further supports our inference that an alloy rather than a core–shell structure has formed in the In_0.50_Ga_0.50_P QDs. The 25 kHz MAS ^69^Ga quadrupolar Carr–Purcell Meiboom–Gill (QCPMG) spectrum of In_0.50_Ga_0.50_P QDs is displayed in Figure 3C. Again, the shape of the peak suggests that there is a distribution of quadrupolar coupling values (Table S4). We do not observe separated domains in the local coordination environment around Ga that are fully ordered, consistent with random alloying of In and Ga in the In_0.50_Ga_0.50_P QDs.^62^ The satellite peaks at −50 and 500 ppm are sidebands from magic angle spinning.

Finally, ^1^H → ^31^P{^115^In} and ^1^H → ^31^P{^69^Ga} CP *J*-resolved experiments were used to confirm alloying of Ga into the phosphide core of the In_0.50_Ga_0.50_P QDs. In these experiments, the total ^31^P spin-echo duration is incremented. For each spin-echo duration, the ^31^P phosphide integrated NMR signal intensity is measured with and without saturation pulses applied on the ^115^In/^69^Ga channels to obtain dephased (*S*) and control (*S*_0_) spectra. Plotting the normalized *J*-dephasing curve gives insight into the distribution of In and Ga in the InP and In_0.50_Ga_0.50_P QDs (Figure 3D,E). The dephasing should be maximized when τ = 1/*J* (assuming a single value of ^1^*J*(^31^P, ^115^In) and/or ^1^*J*(^31^P, ^69^Ga)). It has previously been reported that ^1^*J*(^31^P, ^115^In) is approximately 225 Hz within InP.^51^ However, the ^31^P{^115^In} *J*-dephasing curve for InP QD shows a maximum at a *J*-coupling evolution time of 400 μs, which would indicate ^1^*J*(^31^P, ^115^In) ≈ 2500 Hz. We note that the ^31^P *T*_2_′ is quite short in this sample, leading to a large uncertainty in the signal intensity and an apparent reduction in *J*-dephasing at longer *J*-evolution times. Regardless, the most important observation is that at a short *J*-evolution time of 400 μs, the normalized *J*-dephasing is already 45% for the InP QDs. In comparison, the ^31^P{^115^In} *J*-dephasing curve of In_0.50_Ga_0.50_P QDs shows a much slower build-up with the maximum dephasing occurring at approximately 4 ms total *J*-evolution time, consistent with the literature-reported values of ^1^*J*(^31^P, ^115^In) ≈ 250 Hz for InP.^51,63−68^ Comparison of the ^31^P{^115^In} *J*-dephasing observed at short *J*-evolution times for InP and In_0.50_Ga_0.50_P QD shows a significant reduction in dephasing in the latter. This reduction in dephasing is attributed to a displacement of In by alloying of Ga into the phosphide core; fewer P atoms directly bonded to In atoms will lead to reduced *J*-dephasing. Consistent with this hypothesis, there is significant *J*-dephasing observed in the ^1^H → ^31^P{^69^Ga} CP *J*-resolved experiments performed on In_0.50_Ga_0.50_P QDs. The maximum *J*-dephasing reaches a value of *ca.* 40% at ∼5.6 ms of *J*-evolution. Considering that ^69^Ga has only 60% natural abundance, the observed extent of dephasing is suggestive of alloying of Ga into the phosphide core. Finally, ^1^H{^115^In} and ^1^H{^69^Ga} rotational echo saturation pulse double resonance (RESPDOR) experiments were performed on In_0.50_Ga_0.50_P QDs to determine if the ^1^H spins associated with organic ligands are dipole coupled to both types of metal nuclei. For both ^115^In and ^69^Ga, significant dipolar dephasing was observed, suggesting that both types of metals are present near the surface of the particles (Figure S10). To summarize, the ssNMR experiments show that the In_1–*x*_Ga*_x_*P nanocrystals we prepared via molten-salt processing are largely devoid of surface oxidation; the extent of oxidation of the phosphide phase is overall negligible. Further, the Ga distribution is likely homogeneous across the breadth of the nanocrystals. While ssNMR data cannot rule out some local inhomogeneities in the Ga-to-In ratio, it can be concluded that In_0.50_Ga_0.50_P QDs adopt an alloyed ternary III–V phase rather than a heterostructured morphology composed of InP and GaP domains.

### Evolution of Optoelectronic Properties as a Function of Ga Composition

**Figure 4:**
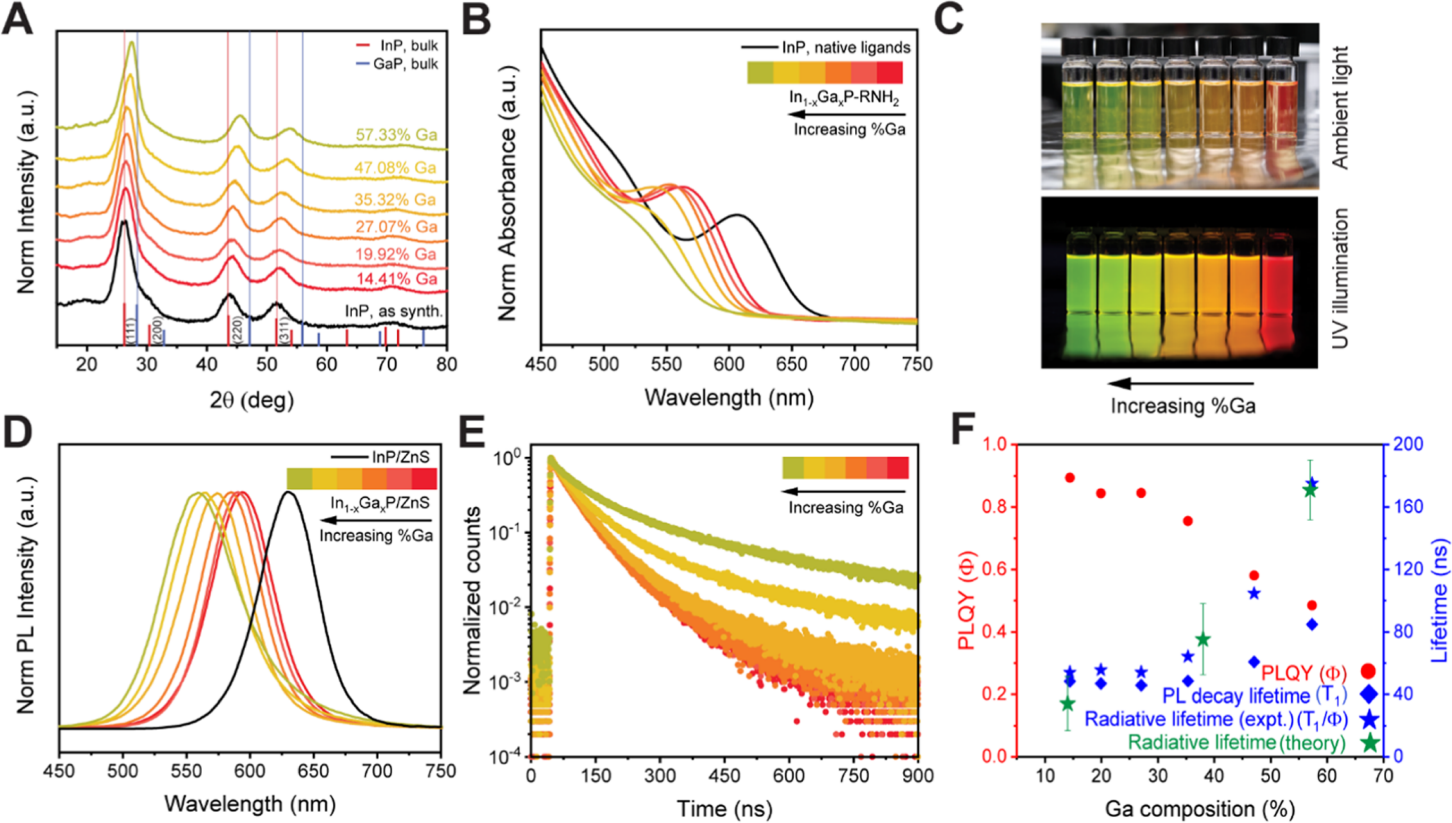
(A) Powder X-ray diffraction patterns of In_1–*x*_Ga*_x_*P cores derived from the 4 nm InP nanocrystals. (B) Absorption spectra of the alloyed In_1–*x*_Ga*_x_*P cores (normalized at 400 nm) indicate a diminishing excitonic feature with an increase in gallium composition and a blue shift of the absorption onset. (C) Photographs show the large range of emission colors produced by core–shell In_1–*x*_Ga*_x_*P/ZnS samples with varying gallium contents derived from the same 4.0 nm InP nanocrystals. (Red emission is from the InP/ZnS sample corresponding to *x* = 0.) (D) The corresponding emission spectra. (E) Time-resolved PL dynamics integrated over the emission spectra of the same samples indicate an increasing decay lifetime with gallium content. (F) The trends in PLQY decay time constants pertaining to the predominant excitonic decay event and the corresponding radiative lifetime. The calculated radiative lifetimes for In_1–*x*_Ga*_x_*P QDs of random gallium distributions are shown to agree well with the experimental results. The trace colors in panels (B), (D), and (E) are the same as in panel (A).

Equipped with an oxygen-free shelling protocol that yields good-quality emissive samples, we next seek to understand how the optoelectronic properties of these core–shell samples evolve as a function of gallium composition. Starting with the 4 nm InP nanocrystals, we synthesized a series of alloyed In_1–*x*_Ga*_x_*P nanocrystals by performing In-to-Ga cation exchange in a KGaI_4_ molten-salt reaction medium under different combinations of annealing temperature and duration, as summarized in Table S5. The amine-capped In_1–*x*_Ga*_x_*P nanocrystals were recovered as a colloidal solution in toluene and characterized *via* X-ray diffraction; subsequently, the gallium compositions were estimated to be between 14 and 60% (Figure 4A). We performed elemental analysis using X-ray fluorescence (XRF) on the In_1–*x*_Ga*_x_*P/ZnS core–shell samples, and the results are included in Table S5. For each sample, the elemental analysis method estimates the gallium content at ∼25% higher than that estimated by employing XRD measurement of the lattice constant and subsequent Vegard’s law interpolation of the gallium content. We have previously observed a similar discrepancy in gallium composition reported by elemental analysis techniques (XRF or inductively coupled plasma (ICP)) and powder XRD (PXRD) for small nanocrystals with a large surface-to-volume ratio.^36^ The 4 nm diameter QDs used in this study have approximately 25% of their atoms at the surface. Thus, one can imagine that changes in the composition of the outer surface layer can dramatically change the QD composition with only a minor impact on the QD interior. For example, if a surface monolayer of metal-rich InP exchanges to GaP, the elemental composition could be up to 25% gallium, while the XRD pattern would show only minor changes, resulting primarily from the surface strain rather than bulk lattice reorganization. As expected, absorption peaks shift monotonically to higher energies with an increasing gallium content, indicative of a widening band gap. Notably, we observe a continuous decline in the sharpness of the excitonic feature with an increasing gallium content (Figure 4B). We can attribute this observation either to the broadening of nanocrystal size distribution over the course of high-temperature annealing or to the mixing of excitonic states from direct and indirect valleys in the conduction band. Photographs of the particles under UV illumination (Figure 4C) show that we can achieve bright emission in a wide range of colors between red and green by varying gallium compositions between 0% and 58% for 4 nm diameter cores. The recorded PL spectra corroborate our visual observations: a blue shift of the emission peak accompanies the increasing gallium content (Figure 4D).

To quantitatively rationalize the experimentally observed optoelectronic properties of In_1–*x*_Ga*_x_*P QDs, the electronic structure and optical properties of alloyed In_1–*x*_Ga*_x_*P QDs are calculated using atomistic semi-empirical pseudopotential methods^69−72^ combined with the Bethe–Salpeter equation (BSE).^73,74^ To yield a unified description of inter-atomic interactions in the alloyed systems, single atomistic pseudopotentials each for In, Ga, P, and As were fitted to accurately reproduce the bulk band structures and bulk deformation potentials of relevant binary semiconductors (*i.e.*, InP, GaP, InAs, and GaAs).^71,75^ The pseudopotential has a local form in the reciprocal space, with a prefactor to account for local strain effects. The alloyed In_1–*x*_Ga*_x_*P QD configurations were constructed by randomly replacing In cations in a regular tetrahedral InP QD with Ga cations. The QD structures were then relaxed using a previously parameterized Tersoff-type force field^76^ and passivated with pseudo-ligand potentials. Given the relaxed QD geometry, the single particle Hamiltonian was constructed with the fitted atomistic pseudopotentials and diagonalized using the stochastic filter diagonalization technique to obtain quasiparticle states of the QD near the highest occupied molecular orbital (HOMO) and lowest unoccupied molecular orbital (LUMO) energies.^72^ The Bethe–Salpeter equation was solved within the static screening approximation to obtain correlated electron–hole pair (i.e., exciton) states.^74^ Our theoretical approach allows for joint investigations and direct comparisons between theory predictions and experimental measurements as it permits accurate atomistic calculations of the optoelectronic properties for In_1–*x*_Ga*_x_*P nanocrystals with arbitrary gallium composition with experimentally relevant sizes. More details on the semi-empirical pseudopotential method, pseudopotential fitting to bulk III–V binary semiconductors, the Bethe–Salpeter equation, and the equilibrium geometries of the alloyed In_1–*x*_Ga*_x_*P QDs can be found in the SI.

Electronic structure calculations confirm the experimentally observed peak shifts. As shown in Figure 1B, the calculated optical gaps for In_1–*x*_Ga*_x_*P QDs show a monotonic increase in energy with higher gallium concentration and a similar dependence on the average lattice constant as compared to the bulk alloyed In_1–*x*_Ga*_x_*P. The vertical shift of the optical gap between the QDs and bulk ternary alloys is attributed to the confinement energy of QD systems. The slope of the QD optical gap changes at 65% gallium composition, indicating an equivalent transition between direct- and indirect-gap regimes as in the bulk alloy In_1–*x*_Ga*_x_*P (blue line). And the shift of the transition point from 75% in the bulk to 65% for 4 nm QDs can be explained by the quantum confinement and the much smaller electron effective mass in the Γ valley as compared to the effective mass in the X and L valleys. In addition, spatially inhomogeneous In-to-Ga cation exchange is studied through theoretical calculations. As shown in Figure S2, the calculated optical properties (namely, optical gap, oscillator strengths, and absorption spectra) for QDs with Ga-rich surfaces differ largely from experimental and theoretical results for QDs with spatially random Ga distributions. These results are consistent with our ssNMR studies, suggesting that Ga is homogeneously distributed in the particle.

**Figure 5:**
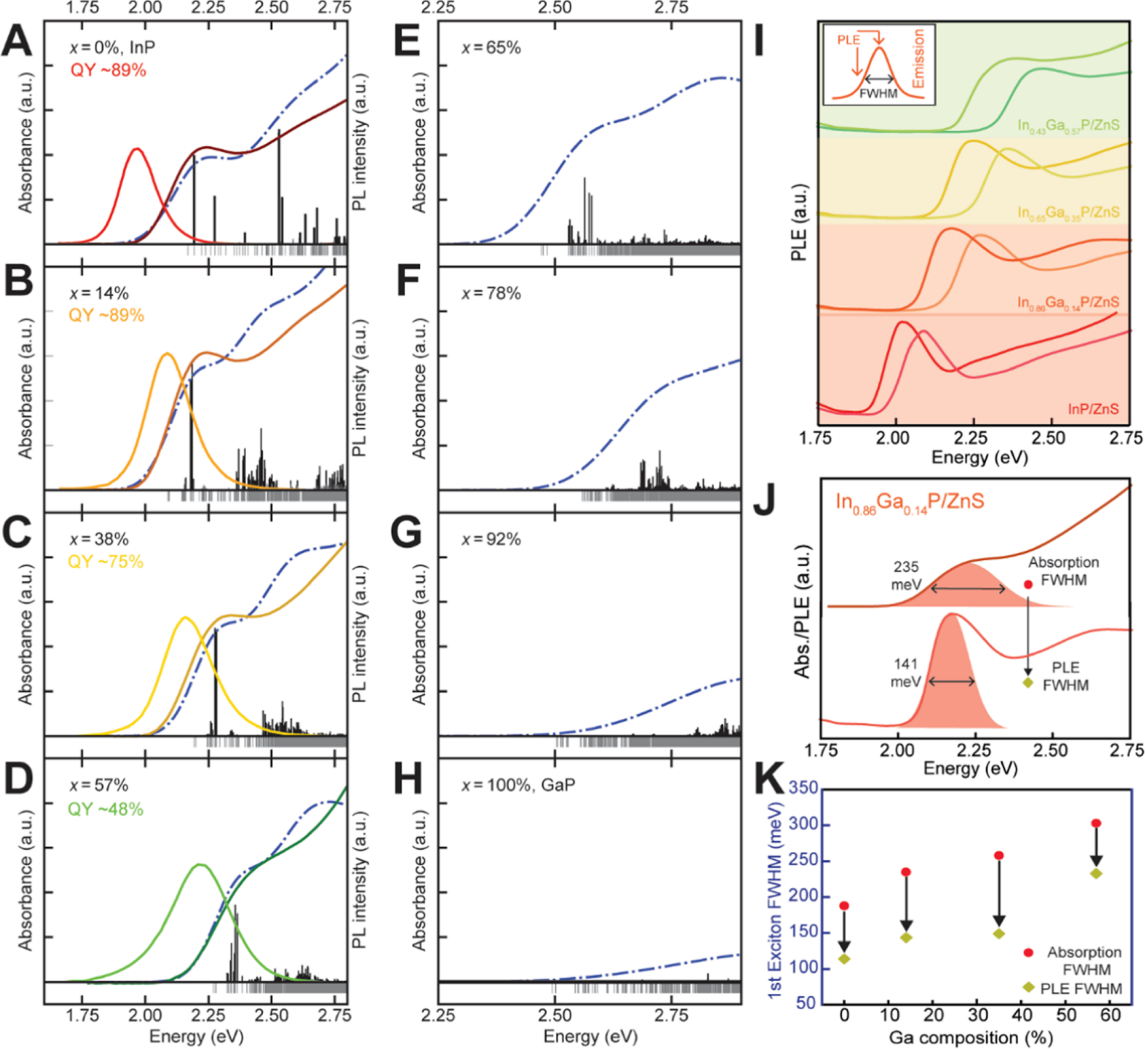
(A–H) Experimental absorption, emission spectra (solid-colored lines), and calculated absorption spectra (blue dash-dot curves) of representative core–shell In_1–*x*_Ga*_x_*P/ZnS samples with 0–57% Ga content, synthesized from 4 nm InP QDs. The exciton density of states and oscillator strengths from theoretical predictions are plotted as gray sticks below the *x*-axis and black sticks above the *x*-axis, respectively. The height of the sticks for the oscillator strengths are on the same y-scale for panels A-E allowing for direct comparson of the change in oscillator strength for different compositions. (I) PL excitation (PLE) spectra of the same samples were collected at the corresponding emission maxima as well as at half the maximum emission intensity on the high-energy flank. The inset plot describes the same schematically. (J) Comparison of line widths of the first excitonic feature on the ensemble absorption spectrum and PLE spectrum of the representative In_0.86_Ga_0.14_P/ZnS sample, collected at the emission maximum. The line widths are estimated as the FWHM of Gaussian fits (shaded) to the low energy flanks of the respective peaks. (K) Summary and comparison of absorption and PLE line widths extracted for the representative core–shell In_1–*x*_Ga*_x_*P/ZnS samples noted in (A)–(D).

We carried out steady-state absorption and PL spectroscopy on each sample, and a few representative experimental absorption and emission spectra from these measurements are plotted in Figures 4B,D and S11 for samples in the *x* range of 0–58%. In_0.86_Ga_0.14_P/ZnS is the brightest sample among the alloyed populations, registering a quantum yield of 89% along with good color purity (57 nm FWHM of the emission peak). For the 4 nm nanocrystals studied so far, it is difficult to incorporate larger amounts of gallium (*x* > 58%) due to high-temperature decomposition. However, we analyzed all possible gallium compositions computationally for 4 nm diameter In_1–*x*_Ga*_x_*P nanocrystals by calculating the absorption spectra of InP QD, GaP QD, and alloyed In_1–*x*_Ga*_x_*P QDs ranging from 14 to 92% gallium content using the atomistic pseudopotential methods, as shown in Figure 5A–H. To generate the absorption spectra, the oscillator strength  (where **μ** is the transition dipole moment and *E* is the exciton energy) for each excitonic state (gray lower lines) is calculated, and its magnitude is represented by the height of the upper black lines in Figure 5A–H. For all of the samples with gallium content below 57%, the majority of the low energy states in the density of states (DOS) have large oscillator strength, consistent with the strong excitonic emission observed for each excitonic transition^77^ (upper black lines).

For In_1–*x*_Ga*_x_*P QDs with *x* > 57%, the computation results show a continued decrease in the sharpness of the absorption onset as the gallium content increases (Figure 5E–H). The decrease in the magnitude of the absorption onset is a result of the decrease in oscillator strength of the lowest-energy excitonic transitions, consistent with indirect-like electronic transitions. This shows that extensive gallium exchange creates many low-energy “dim” excitonic states at the band edge, which is indicative of optical transitions with a more “indirect” character.

Calculated absorption spectra are consistent with the experimental observation that the sharpness of the excitonic features declines as gallium content increases. Importantly the theoretical observations are not convoluted with other sources of broadening, such as size distribution or composition distributions. Although ssNMR studies establish that the In and Ga atoms are randomly distributed at the cation sites of the ternary III–V lattice, particle-to-particle compositional variation remains a possible source of added heterogeneity in alloyed nanocrystals. We have previously established that ensemble diffraction techniques are insensitive to these subtle structural differences,^37^ but the emissive core–shell In_1–*x*_Ga*_x_*P/ZnS nanocrystals allow us to explore this optically. Recording PL excitation (PLE) spectra allows us to selectively look at the spectral features of a smaller subpopulation instead of the entire ensemble. We, therefore, seek to compare the spectral heterogeneity of these alloyed nanocrystals using the corresponding absorption and PLE line widths for excitonic transitions. To this end, we constructed excitation–emission maps (Figure S11), allowing us to extract representative PLE spectra at the corresponding emission maxima, as well as at the point of half the maximum emission intensity on the high-energy flank (Figure 5I). We can further quantify the PLE and ensemble absorption line widths using the full width at half-maximum (FWHM) of Gaussian fits to the low energy flanks of the respective peaks. Figure 5J explains this analytic scheme for a representative sample In_0.86_Ga_0.14_P/ZnS; the results for these In_1–*x*_Ga*_x_*P/ZnS samples are summarized in Figure 5K. It is evident that each individual PLE spectrum becomes broader at higher gallium concentrations. Ensemble broadening from population ripening at elevated temperatures alone cannot explain the broadening of excitonic features in individual PLE spectra. Previous computational and experimental works have established that the energy states of homogeneously alloyed nanocrystals are not simple arithmetic means of their individual components.^78,79^ Instead, atomic disorder-related symmetry breaking can lead to the formation of additional mixed states, resulting in broadened spectral features during excitonic transitions, consistent with our observations. Furthermore, the theoretical model in this work incorporates the atomistic details and local strain effects through atomistic pseudopotentials, and thus, the “mixing of excitonic states” of the alloyed system is directly captured by our electronic structure calculations and can be observed from the density of states in Figure 5A–H. Sample inhomogeneity is accounted for in the calculated absorption spectra through the energy-dependent Gaussian broadening and is kept the same across different Ga compositions. Therefore, the theoretical calculations in this work help conclude that exciton state mixing due to symmetry breaking is the main contributor to the broadened absorption features in In_1–*x*_Ga*_x_*P QDs with high Ga composition. We probe this in more detail with transient absorption studies, and the results are summarized in a later section.

We estimate the PL decay lifetimes from time-resolved PL studies (Figure 4E). Time-resolved photoluminescence (TRPL) of the In_1–*x*_Ga*_x_*P/ZnS samples with varying sizes and Ga-to-In ratios does not show any fast early-time (sub-nanosecond) decay components usually associated with non-radiative recombination channels, which is typical for samples with high PL quantum yield. The faster radiative decay channel is the major contributor in each of these high-quality core–shell samples from a subsequent biexponential fit to the decay profile, shown in Figures S8 and S11. A comparison of the TRPL decay profiles of these In_1–*x*_Ga*_x_*P/ZnS samples in Figure 4E illustrates a remarkable trend: the PL decay lifetime increases monotonically, while the PLQY decreases with increasing gallium content, which is difficult to explain by a trapping mechanism. We estimate the radiative lifetime by combining the PL 1/*e* decay time constant and PLQY, providing us with an insight into the materials’ intrinsic radiative lifetime (Figure 4F). Importantly by directly measuring the radiative lifetimes, the trends we observe are independent of the quality of surface passivation during synthesis or other defects, which can lead to non-radiative recombination.

Radiative lifetimes at room temperature are calculated from the exciton energy and transition dipole moment for the calculated exciton states according to the equation , where *c* is the speed of light, and ℏω is the exciton energy. For the same gallium percentage, multiple configurations with random gallium distributions are considered, rendering the mean and standard deviation of the radiative lifetimes. See the SI for details on calculating the radiative lifetimes. As summarized in Figure 4F, the trends are quite evident. An addition of up to ∼30% Ga into the In_1–*x*_Ga*_x_*P cores has only a minor effect on radiative lifetime and quantum yield, while a further increase of Ga content up to 60% is accompanied by a decline in PLQY down to 48%, along with a more than 3-fold increase in radiative lifetime, from 54 to 175 ns. The rate of radiative decay is inversely proportional to the calculated radiative lifetime, so we conclude that the oscillator strength of excitonic recombination^80^ in these systems is independent of composition when Ga content is below 35% and gradually decreases with increasing gallium content above 35%. Theoretical calculations in this work show good agreement with experimental measurements and demonstrate the same trend of decreasing absolute absorption intensity (Figure 5A–H) and increased radiative lifetimes (Figure 4F) as Ga concentration increases in alloyed In_1–*x*_Ga*_x_*P QDs of the same size. A direct-gap-like excitonic transition characterized by a large oscillator strength would be from the Γ valley of the conduction band to the Γ valley of the valence band. In bulk crystals, the oscillator strength for the transitions from X or L valleys to Γ valley is significantly lower. We ascribe the diminishing oscillator strength of excitonic recombination in Ga-rich In_1–*x*_Ga*_x_*P/ZnS QDs to an increased mixing of electron states originating from the X and Γ valleys. In bulk In_1–*x*_Ga*_x_*P crystals, the direct-to-indirect transition is observed at *x* ∼ 0.75 gallium content (Figure 1), thus motivating exploration of quantum-confined samples with even higher gallium contents.

### Trends at Higher Ga Content

**Figure 6:**
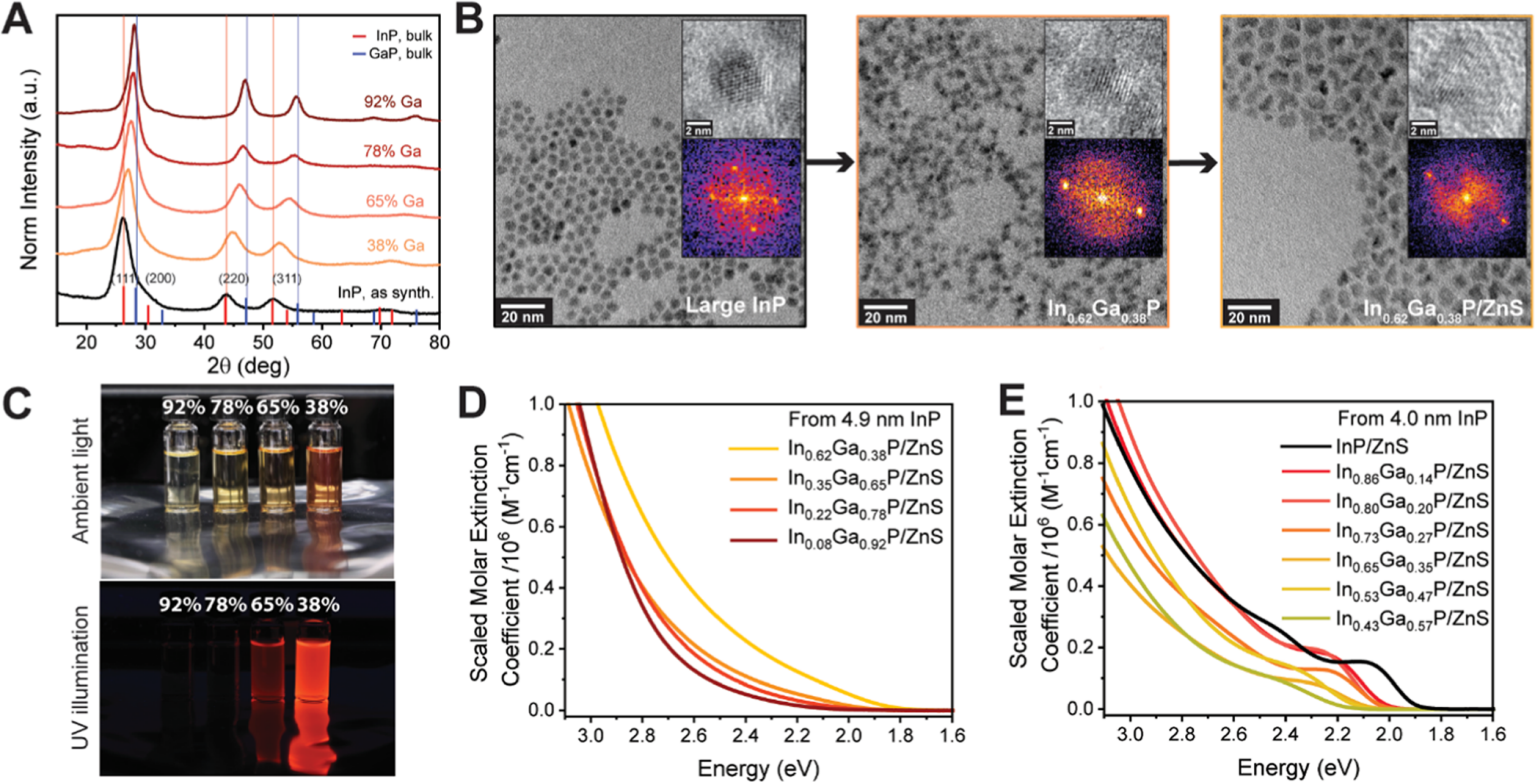
(A) PXRD patterns of In_1–*x*_Ga*_x_*P cores with high gallium content derived from the 4.9 nm InP population. (B) Representative TEM images of the InP, In_0.62_Ga_0.38_P, and In_0.62_Ga_0.38_P/ZnS samples indicate the evolution of particle morphology during the cation exchange and ZnS shell growth. (C) Photographs of the relevant core–shell samples derived from the same 4.9 nm InP population in ambient light and under UV illumination. Absorbance plots were scaled to the measured absorption cross-section at 400 nm (3.1 eV) to yield molar extinction coefficient plots of representative In_1–*x*_Ga*_x_*P/ZnS samples synthesized from the (D) 4.9 nm InP population and (E) 4.0 nm InP population.

Our studies suggest that the larger InP nanocrystals are more resistant toward decomposition under similar reaction conditions as compared to smaller nanocrystals, whereas higher temperatures and longer annealing times are required to achieve high gallium contents. We, therefore, focus on particles derived from the large 4.9 nm InP population to achieve higher gallium contents. Bright In_0.62_Ga_0.38_P/ZnS samples could be synthesized from 4.9 nm InP cores, as shown in Figures 2B and S7. We further prepared three populations of cation-exchanged In_1–*x*_Ga*_x_*P cores in the 65–92% gallium content range; the reaction conditions are listed in Table S6. The nanocrystalline phases were characterized via PXRD, and gallium compositions were estimated from the diffraction patterns (Figure 6A). Representative TEM images provided in Figure 6B confirm that we have grown a widegap ZnS shell on the ternary alloyed nanocrystals; detailed reaction conditions are provided in the Supporting Information. Steady-state optical characterization of the core–shell In_0.35_Ga_0.65_P/ZnS sample (Figure S12A) indicates that this sample is weakly emissive, with a PLQY of 2%. The corresponding excitation–emission map is provided in Figure S12B.

An excitonic feature is not resolved in the absorption spectra of samples with even higher gallium content, In_0.22_Ga_0.78_P/ZnS and In_0.08_Ga_0.92_P/ZnS (Figure S12C,E). Notably, these are also completely nonemissive, as the excitation–emission maps indicate (Figure S12D,F). Visual confirmation can be obtained from the photographs in Figure 6C. Using fluence-dependent transient absorption and a Poisson excitation model^6^ (discussed in more detail in the next section), we determined the absorption cross-section and, subsequently, the molar extinction coefficient of each sample^81^ at 400 nm (3.1 eV) (Figure S13). The corresponding absorption spectra were scaled to the extinction coefficients at 400 nm accordingly (Figure 6D). A similar treatment of the absorption spectra of 4 nm core diameter In_1–*x*_Ga*_x_*P/ZnS samples is shown in Figure 6E. This demonstrates that samples with a higher gallium composition show a decrease in absolute absorbance of the excitonic feature. This is consistent with a weakened oscillator strength of excitonic transitions, which was suggested by the increase in radiative lifetime. We conclude that the optically dark In_0.22_Ga_0.78_P/ZnS and In_0.08_Ga_0.92_P/ZnS samples obtained from 4.9 nm InP are potentially behaving as indirect semiconductors, *i.e.*, the Γ and X valleys are the highest valence band and lowest conduction band positions, respectively. We further performed Tauc analysis to estimate the corresponding indirect gaps. A baseline correction for the disorder-induced Urbach tail was performed according to previously prescribed protocols,^82^ thus allowing us to estimate the indirect gaps at 2.71 and 2.68 eV for Γ to X-valley transitions in In_0.22_Ga_0.78_P/ZnS and In_0.08_Ga_0.92_P/ZnS, respectively (Figure S14).

### Transient Absorption (TA) Spectroscopy

**Figure 7:**
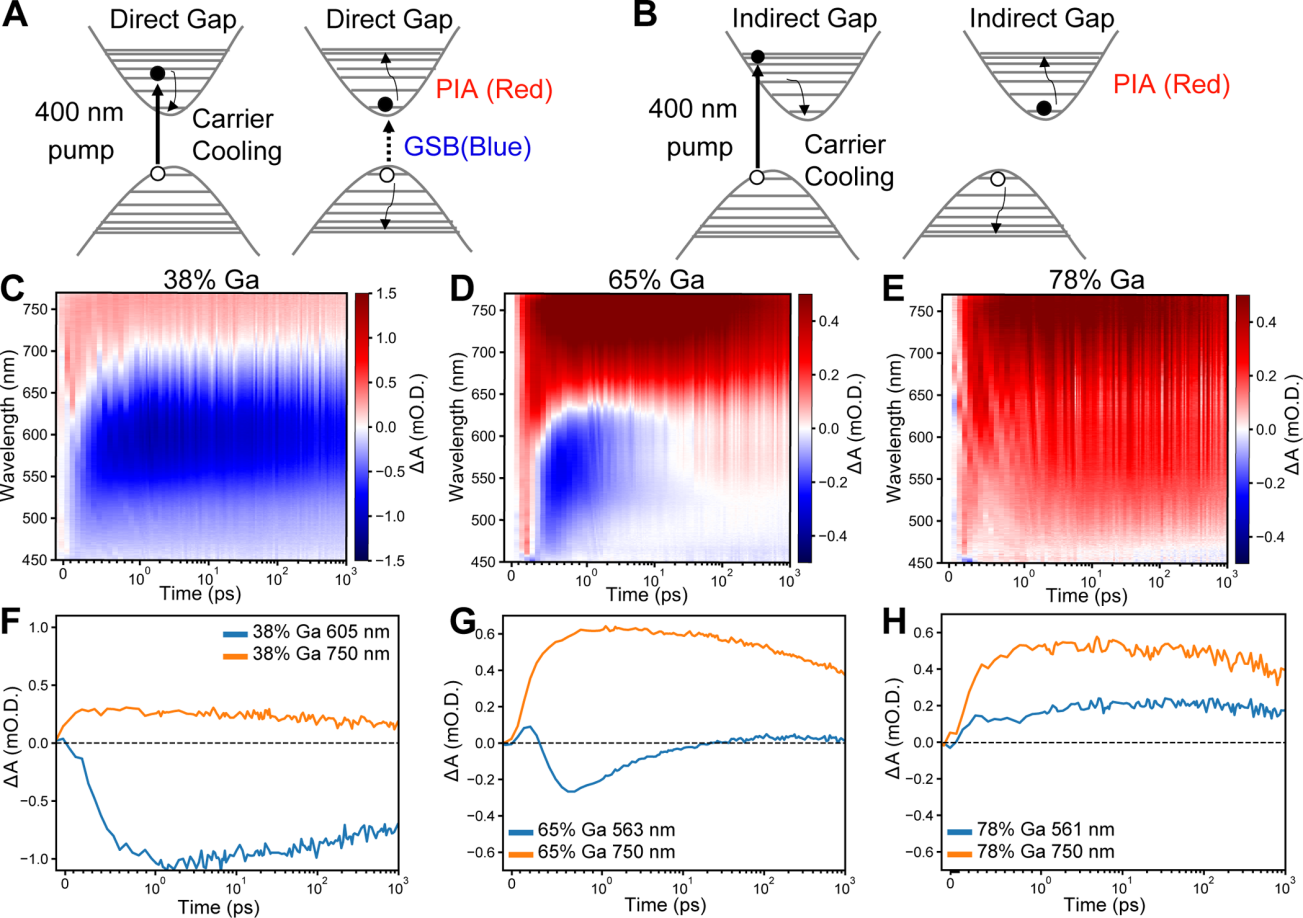
Schematic representation of the expected transient absorption signals expected for (A) a direct-band-gap semiconductor and (B) an indirect-band-gap semiconductor. GSB: ground state bleach, negative Δ*A*; PIA: photoinduced absorption, positive Δ*A*. Low fluence transient absorption dynamics (400 nm pump) are shown for In_1–*x*_Ga*_x_*P/ZnS QDs with (C) 38%, (D) 65%, and (E) 78% gallium content. Line cuts of the TA signal at maximum bleach amplitude as well as PIA traces at 750 nm are shown for large In_1–*x*_Ga*_x_*P/ZnS nanocrystals with (F) 38%, (G) 65%, and (H) 78% gallium content.

We performed femtosecond transient absorption measurements on the In_1–*x*_Ga*_x_*P/ZnS nanocrystals synthesized from 4.9 nm InP cores, which displayed absorption and PL characteristics of direct-gap-like behavior (38% Ga), intermediate behavior (65% Ga), and indirect-gap-like behavior (78% Ga), respectively. Measurements were performed using a 35-fs, 2-kHz Ti:sapphire laser with time-delayed white light probe pulses produced in a 2 mm sapphire plate and 1-kHz pump pulses centered at 400 nm produced from the second harmonic generation of the fundamental. At these pump energies, we are exciting the samples well above the band gap of the In_1–*x*_Ga*_x_*P core but below the absorption onset of the ZnS shell. All time-resolved absorption spectra were collected at room temperature from stirred solutions, first at low excitation fluence, such that only single exciton dynamics were observed. TA data was collected on samples with ZnS shells, so the TA dynamics better reflected intrinsic carrier dynamics rather than charge trapping dynamics. The similarity between the UV–vis absorption feature of unshelled and shelled samples indicates that the presence of the shell has minimal impacts on the band-edge absorption spectra of these materials, in agreement with a type-I band alignment between the core and the shell materials (Figures S11 and S12). Figure 6A,B shows schematic representations of the expected transient absorption signals for direct and indirect band gap materials. The key observation is that a direct-gap semiconductor is expected to show both ground state bleach (GSB) signal and photoinduced absorption (PIA) signal, whereas indirect-gap materials are only expected to show a PIA signal. Figure 7C shows a transient absorption spectroscopy map for In_0.62_Ga_0.38_P/ZnS nanocrystals. We observe a strong ground state bleach signal (negative Δ*A*) peaked at 605 nm, which develops within ∼1 ps after the pump pulse and slowly decays over the nanosecond timescale of the measurement. The bleach signal corresponds well to the excitonic feature we observe by linear absorption of the sample (Figure S7A). This behavior is consistent with a typical direct-band-gap semiconductor nanocrystal where the 1S_e_–1S_h_ excitonic transition is bleached once the photoexcited carrier cools to the band edge. A line cut of the TA signal at the maximum bleach amplitude (Figure 7F) shows slow decay of the bleach signal, consistent with the timescales observed by TRPL. We also observe a photoinduced absorption (PIA) signal at wavelengths to the red of the bleach feature, which results chiefly from electrons in the conduction band undergoing excitation to the near-continuum of states above the band edge by the probe pulse.

Next, we turn our attention to the In_0.22_Ga_0.78_P/ZnS sample, which showed a diffuse absorption onset and lacked detectable photoluminescence at room temperature or 77 K. Transient absorption dynamics for In_0.22_Ga_0.78_P/ZnS are shown in Figure 7E,H. Here, we exclusively observe a PIA signal for all wavelengths and do not detect any trace of a bleach feature in the probed spectral range or timescale. This behavior is consistent with what is expected for indirect-gap materials, where the photoexcited electron and hole reside in different valleys of the conduction and valence band; the momentum mismatch between the electron and hole prevents the formation of an optical bleach signature. The PIA signals measured at 750 and 561 nm both form within 1 ps and slowly decay over the 1 ns measurement window.

Next, we discuss the TA dynamics for the In_0.35_Ga_0.65_P/ZnS sample. This sample showed a diffuse absorption onset and weak PL at room temperature. Transient absorption shows the formation of a bleach signal centered at 563 nm, which has a maximum amplitude at 800 fs. This bleach signal quickly decays within 20 ps such that at later times, only a PIA signal is observed across all wavelengths after ∼30 ps (Figure 7D,G). These carrier dynamics are remarkable since they are not simply a linear combination of direct-like and indirect-like dynamics of the low and high gallium content samples, respectively. One possibility is that after photoexcitation and carrier cooling processes, there is initially a population of carriers in the direct Γ-like valleys as well as a second conduction band state, which has an X-like character with similar or slightly lower energy than the Γ-like valley. Intervalley scattering from the Γ-like valley to the X-like valley within ∼20 ps efficiently removes carriers that can undergo direct transitions, causing the optical bleach signature to quickly decay. Interestingly, the TRPL of the In_0.35_Ga_0.65_P/ZnS sample shows an IRF-limited fast component and also an extremely long decay component (Figure S15), suggesting that scattering back into the direct channel at a late time can lead to PL. The existence of low-energy dark states and higher-energy bright states is consistent with the exciton manifold for the 65% Ga sample shown in Figure 5E.

Fluence-dependent transient absorption dynamics for the samples discussed here are presented in Figure S16; the 38% Ga and 78% Ga samples show characteristics of Auger recombination, as discussed later. The bleach signal of the 65% Ga sample with high pump fluences decays faster than expected for Auger recombination,^11^ indicating that these dynamics originate from a different process. We also pumped the In_0.35_Ga_0.65_P/ZnS sample directly into the lowest energy states using a pulse centered at 640nm (Figure S17) and found that the decay of the optical bleach signature follows similar dynamics as with the 400 nm pump. In this experiment, we preferentially excited the sample into band-edge states that have large oscillator strength. Since we observe similar decay dynamics starting from the band-edge state, we conclude that the carrier population transfer occurs directly from the direct-gap-like state to some other optically inactive state. While the bleach signature decays on the ∼20 ps timescale, the PIA signal remains relatively constant over the 1 ns measurement window, indicating that photoexcited carriers are still present within the nanocrystal but have a momentum mismatch that prevents optical bleaching. Due to the lighter effective mass of the electron, the PIA signal is likely sensitive primarily to the electron population, and thus the retention of the PIA signal despite the fast decay of the bleach is consistent with the transfer of the electron population to X-like valleys. Taken together, the transient absorption results are consistent with the conversion of these QD emitters from direct-like to indirect-like band structures at ∼65% gallium. Near the direct-to-indirect transition composition, the excited-state dynamics can become complicated by nearly isoenergetic states, which have different oscillator strengths.

### State Degeneracy in In_1–*x*_Ga*_x_*P/ZnS Nanocrystals

**Figure 8:**
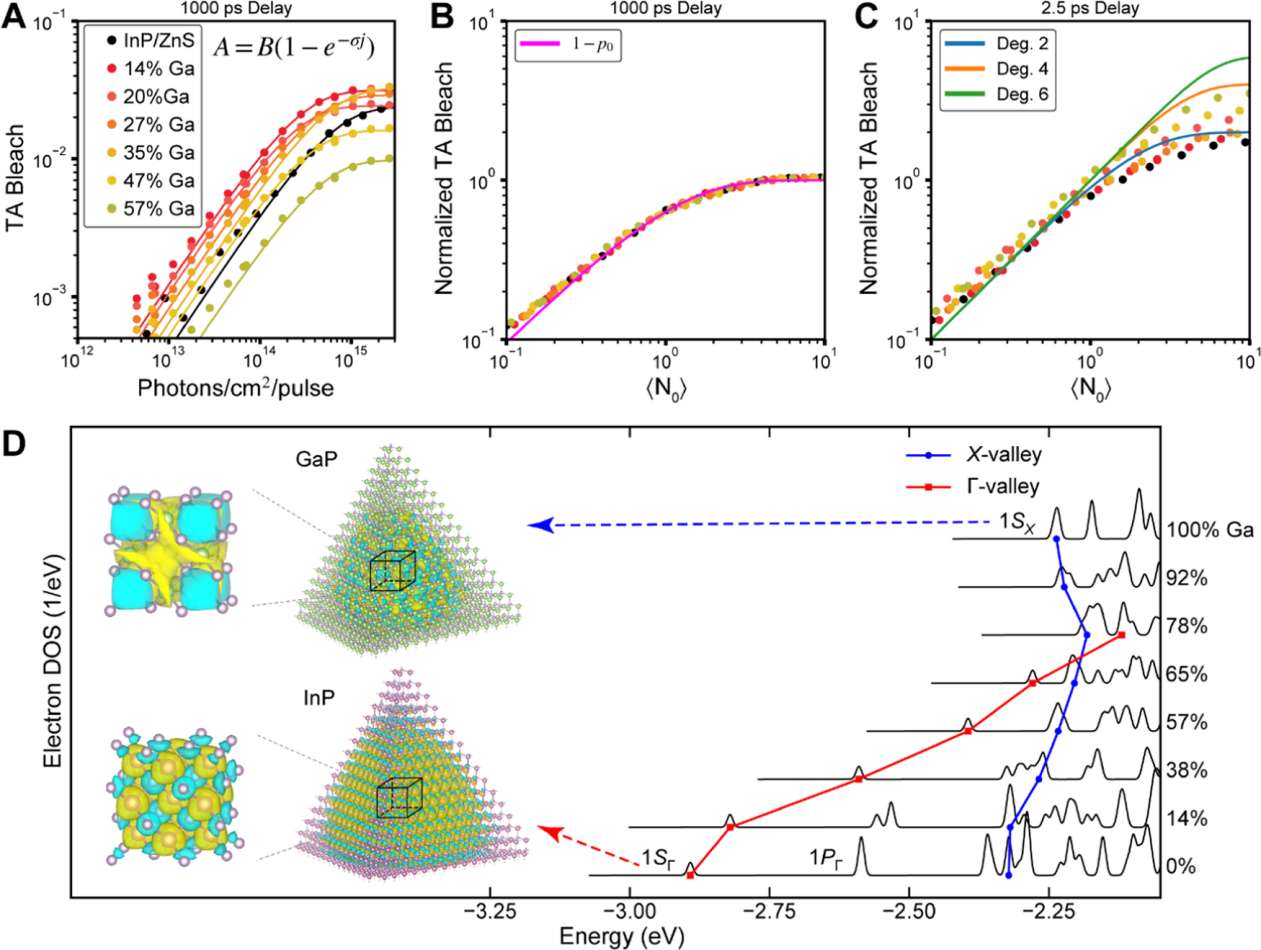
State filling in 4 nm core diameter In_1–*x*_Ga*_x_*P/ZnS nanocrystals. (A) Bleach amplitude (*A*) at 1000 ps delay as a function of pump fluence (circles) with fits to single excitation Poissonian statistics to determine the absorption cross-section (σ) and bleach amplitude scaling factor (*B*) of In_1–*x*_Ga*_x_*P/ZnS nanocrystals with varying gallium contents. (B) The normalized TA bleach signal at 1000 ps delay as a function of the initial average excitation ⟨*N*_0_⟩. The pink line is a Poissonian distribution describing the total number of singly photoexcited nanocrystals that remain after Auger recombination. (C) The normalized TA bleach signal at 2.5 ps delay (before significant impact from Auger recombination) as a function of the initial average excitation ⟨*N*_0_⟩. The blue, orange, and green lines are model Poissonian excitation statistics for degeneracies of 2, 4, and 6, respectively, for the ground state bleach signal. (D) The density of states (DOS) for the conduction band-edge electron states of alloyed QDs are shown. The DOS of each gallium composition is shifted vertically for clarity. Twofold-degenerate Γ-valley-like (1S_Γ_, red squares) and sixfold-degenerate X-valley-like (1S_X_, blue circles) conduction band states and their energies are traced for each gallium content. In the inset, the conduction band-edge electron wavefunctions of tetrahedral InP and GaP QDs (1S_Γ_ and 1S_X_, respectively) are shown. The Bloch wavefunctions are extracted from the center of each QD. Atom colors: pink—In, green—Ga, purple—P. Isosurface colors: yellow—positive, blue—negative.

The previously discussed transient absorption dynamics, TRPL, radiative rate, and molar extinction coefficient measurements indicate that the band-edge electronic state in In_1–*x*_Ga*_x_*P/ZnS with >50% Ga may contain states with indirect-gap-like character, which have a lower oscillator strength. In bulk III–V semiconductors, the degeneracy of the conduction band Γ-valley is 2, the conduction band L valley is 8, and the conduction band X-valley is 6. In strongly quantum-confined semiconductors, the definition of direct *vs* indirect transitions becomes fuzzy owing to translational momentum relaxation. The presence of multiple states arising from the different valleys in the band structure may alter the degeneracy of the band-edge exciton. To understand this effect experimentally, we probe state filling in the In_1–*x*_Ga*_x_*P/ZnS QDs synthesized from 4.0 nm InP cores using fluence-dependent TA. We use the bleach signal for analysis, which reflects the population of the electron states due to the high degeneracy of the valence band and lighter effective mass of the electrons compared to the holes.^83^ First, we determine the absorption cross-section, σ, and bleach amplitude scaling factor by fitting the power-dependent bleach signal at 1000 ps (after Auger recombination, ensuring that only single excitons remain in the QDs) according to the equation *A* = *B*(1 – e^–σ*j*^) where *A* is the magnitude of the TA bleach signal, *B* is the bleach amplitude scaling factor, σ is the absorption cross-section per dot at the pump wavelength, and *j* is the pump laser fluence (Figure 8A).^6^Figure 8B shows that all samples fall on a universal curve for single excitations at long times, using σ and *B* determined from the fits.^77^ In Figure 8C, we use *B* and σ to measure the normalized bleach amplitude for the early-time signal at 2.5 ps (before significant contribution from Auger recombination). For In_1–*x*_Ga*_x_*P/ZnS samples that have up to 35% Ga incorporated (red to orange dots), we observe saturation at a normalized bleach signal of 2, consistent with a band-edge degeneracy of 2. The blue line is a Poissonian excitation model^84^ with a degeneracy of 2 and describes the low gallium content data well. This result is consistent with the behavior of a typical III–V direct-gap semiconductor. For samples with 47 and 57% gallium, we observe deviation of the fluence-dependent bleach signal from excitation statistics for a band-edge degeneracy of 2. Instead, the bleach amplitude continues to increase at high excitation densities before eventually becoming sublinear. The effect is more pronounced in the highest gallium content sample (green, 57% Ga). The data for the 47–57% Ga samples approach the excitation statistics of 4 (orange line). Thus, our data suggest that incorporating gallium into our samples alters the degeneracy of the band-edge state.

The evolution of band-edge state degeneracy as the gallium concentration is increased is further analyzed using atomistic pseudopotential calculations.^200^ The main changes observed in bulk alloys were attributed to changes in the conduction band minima as the system transitions from InP to GaP. In NCs in the strong quantum confinement regime, one has to consider excitonic effects; however, since the calculated excitonic states near the onset of absorption comprise mainly a single quasi-electron state at any gallium composition (see the SI for details), it is sufficient to analyze the quasi-electron structure as the content of Ga varies. Figure 8D shows the results for In_1–*x*_Ga*_x_*P QDs constructed from 4.0 nm InP NC. At 0% gallium (i.e., InP QD), the first several electron states are the 2-fold and 6-fold degenerate states with envelope function of 1S and 1P shapes, as expected from the effective mass approximation.^5^ Meanwhile, at 100% gallium (i.e., GaP QD), the lowest electron states are 6-fold degenerate. These states in GaP also have spherical 1S envelope functions, but their Bloch wavefunctions (extracted from the center of the QD) have significantly different symmetry and spatial attributes (as shown in Figure 8D insets). Specifically, in the tetrahedral InP QD, the lowest quasi-electron density is centered on each atom, while in the GaP QD, it is centered around the tetrahedral cavity formed by four neighboring P atoms. The two Bloch functions and their corresponding electron states are named “Γ-valley” and “X-valley”, respectively.

Using the symmetry and spatial features of the Bloch wavefunctions, we identify and track the 1S_Γ_ and 1S_X_ electron states as gallium content increases. As shown in Figure 8D, the energy of 1S_Γ_ electron states increases faster than 1S_X_, leading to a crossover of the Γ-valley-like to X-valley-like band-edge electron state at 65–78% gallium content, in agreement with the experimental observations that the direct-to-indirect transition in In_1–*x*_Ga*_x_*P QDs occur around *x* = 65–78% and helps decipher the atomistic details in its electronic structures. The electronic structure calculations allow us to better interpret the observed increase in state degeneracy measured by TA. Formally the calculations show that the lowest 1S_Γ_ state is 2-fold degenerate and the 1S_X_ state is 6-fold degenerate, not exactly the ∼4-fold degeneracy inferred by TA. We notice that as the gallium content in In_1–*x*_Ga*_x_*P is increased, the splitting between the 1S_Γ_ and 1S_X_ electron states decreases. Both the 1S_Γ_ and 1S_X_ electron states give rise to the manifold of bright excitonic transitions observed in Figure 5D. Due to the close spacing of the states, the bleach observed is actually measuring the state filling of the manifold of closely spaced states with mixed 1S_Γ_ and 1S_X_ characters. Thus, based on our measurements, in the 57% gallium sample, up to four excitons can reside near the band edge simultaneously. In support of this interpretation, we observe a redshift of the bleach feature from early time to late time, suggesting that as Auger removes the multicarrier population from higher energy states in the manifold, only the exciton in the lowest energy state remains (Figure S18).

### Multiexciton Dynamics in In_1–*x*_Ga*_x_*P/ZnS Nanocrystals

**Figure 9:**
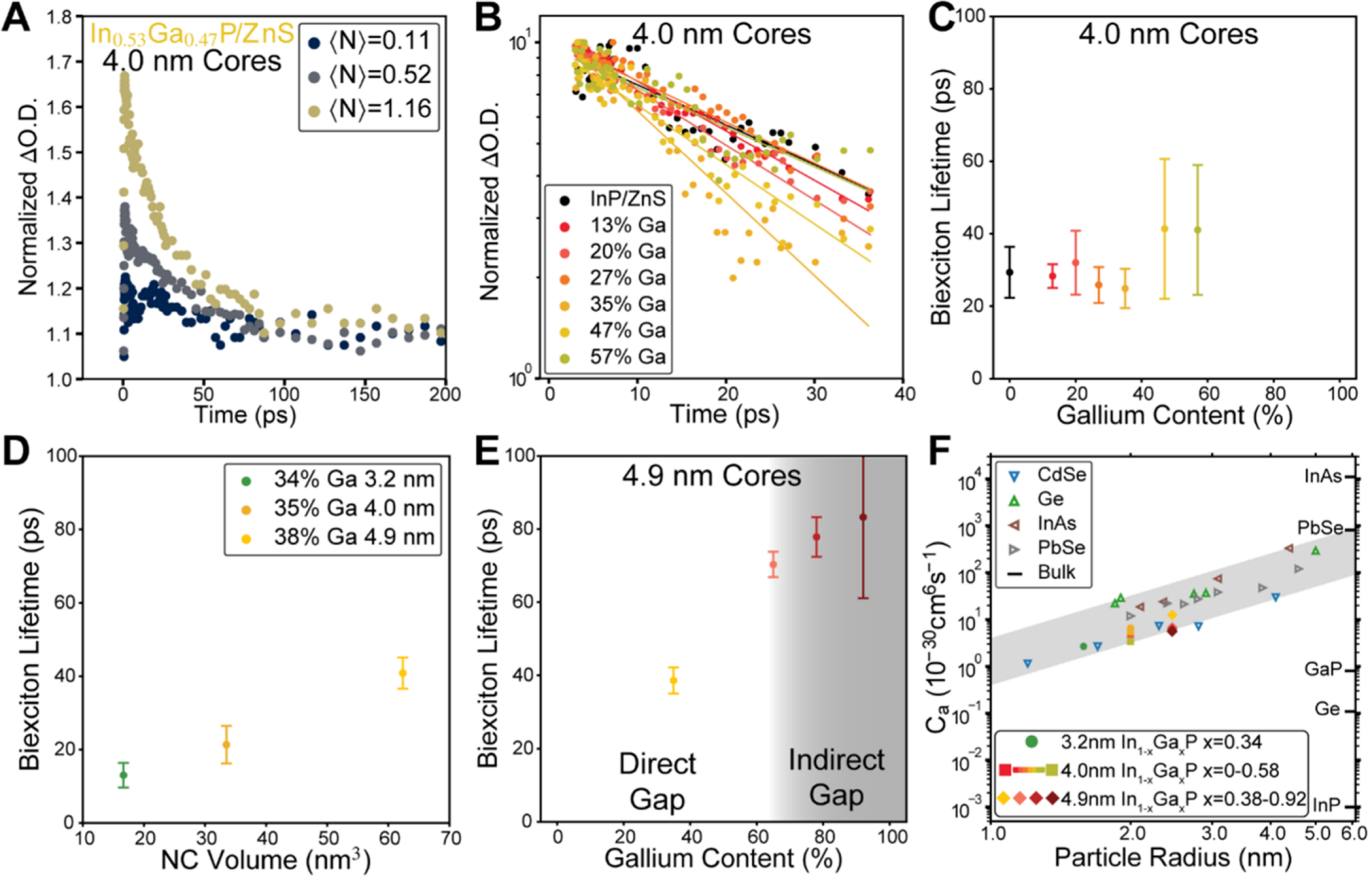
Multiexciton dynamics of In_1–*x*_Ga*_x_*P/ZnS QDs. (A) Power-dependent photoinduced bleach dynamics for ∼4 nm core diameter In_0.53_Ga_0.47_P/ZnS QDs normalized by the signal at long times (1–2 ns average). (B) Difference between high and low fluence bleach dynamics normalized at long times (1–2 ns average) plotted on a log-linear scale for ∼4 nm core diameter In_1–*x*_Ga*_x_*P/ZnS nanocrystals with varying Ga contents. The solid lines are single exponential fits to the data from which the biexciton lifetimes are extracted. (C) Measured biexciton lifetime for 4 nm core diameter In_1–*x*_Ga*_x_*P/ZnS QDs with different gallium contents. The biexciton lifetime displayed is the average of the biexciton lifetime determined using the bleach signal and the photoinduced absorption signal (Figure S24). (D) Biexciton lifetime for In_1–*x*_Ga*_x_*P/ZnS QDs with similar gallium content but different core sizes, showing linear volume scaling. (E) Biexciton lifetimes extracted using the photoinduced absorption signal for 4.9 nm diameter In_1–*x*_Ga*_x_*P/ZnS with high gallium content. (F) Effective Auger constants calculated for the samples measured in this study plotted with literature values for a variety of direct- and indirect-gap semiconductor nanocrystals and the Auger constants of corresponding bulk semiconductors.

In the previous sections, we have elucidated that the band-edge electronic structure is significantly altered in In_1–*x*_Ga*_x_*P/ZnS nanocrystals as more gallium is incorporated. Next, we aim to understand how multicarrier dynamics^85^ are modified by the incorporation of gallium as a function of nanocrystal composition and size. We probe this by measuring fluence-dependent early-time transient absorption dynamics. Figure 9A shows the early-time dynamics of the TA bleach feature in In_0.53_Ga_0.47_P/ZnS nanocrystals for pump fluences which give ⟨*N*_0_⟩, from 0.11 to 1.16. In this figure, the TA signal has been normalized for the signal averaged at 1000–2000 ps.^6^ We observe a fast decay component that increases in amplitude for high pump fluences, indicating significant bi- and multiexciton populations. The observed decay is consistent with a fast Auger recombination, which is typical for both direct- and indirect-gap semiconductor nanocrystals. Next, we measure the biexciton kinetics for In_1–*x*_Ga*_x_*P/ZnS QDs with a 4 nm diameter core and gallium content from 0% to 57%. Data are extracted from fluence-dependent TA maps (Figures S19–S23). Figure 9B shows the normalized TA bleach signal as a function of time on a semi-log scale for all samples with 0–57% gallium. Single exponential fits to the first 35 ps of the bleach decay are used to extract the biexciton lifetime from the bleach signal (Figure S24A,C). Across all samples, we find biexciton lifetimes of ∼30 ps with a significant spread in the data. Importantly, we do not find a significant trend relating gallium content with the biexciton lifetime. We also extracted biexciton lifetimes using the PIA signal for all samples (Figure S24B,D) and found general agreement among the lower gallium content samples (up to 35% Ga). We measure significantly longer biexciton lifetimes for the samples with Ga contents of 47% and 57%, at 57 and 45 ps, respectively, using the PIA signal. This difference parallels our observation that the high gallium content 4.0 nm core In_1–*x*_Ga*_x_*P/ZnS QDs display excited-state dynamics, which are distinct from those of typical direct-gap semiconductor nanocrystals. In Figure 9C, we plot the biexciton lifetime as an average of the lifetimes measured using bleach and PIA signals. The large error bars seen for the 47 and 57% Ga samples are a result of the very different values determined with the two signals. Importantly, we do not observe a meaningful trend with biexciton lifetime and increased gallium content, suggesting that simple volume scaling, discussed later in more detail, continues to be the main factor governing biexciton lifetimes and Auger recombination in quantum-confined semiconductors.

The difference in measured biexciton lifetime for high gallium content samples using the bleach and PIA signals is perplexing. One possibility is that the bleach and PIA signals are sensitive to different excited-state populations. The bleach signal is sensitive only to excitons with a direct-like character, while the PIA signal is sensitive to all photoexcited electrons regardless of their momentum match to the holes. As a result, the photoinduced bleach signal probing primarily biexciton states in the Γ-like valley provides the lower bound on biexciton lifetimes. Such an observation suggests that the biexciton lifetime in samples near the direct-to-indirect transition can be changed by approximately a factor of 2 due to details of the band structure. This is consistent with an approximate factor of 2 increase in the biexciton lifetime for indirect-behaving samples with 78–92% gallium, as discussed later.

Next, we explore the biexciton lifetime as the core size of In_1–*x*_Ga*_x_*P/ZnS QDs is varied but with similar gallium content. To this end, we measured the biexciton lifetime for In_1–*x*_Ga*_x_*P/ZnS QDs with nominal core diameters of 3.2, 4.0, and 4.9 nm and measured gallium contents of 34, 35, and 38% gallium, respectively. The measured biexciton lifetime using the bleach signal for fitting (Figure S25A,C) as a function of particle volume is shown in Figure 9D. Here, we observe a linear scaling of the biexciton lifetime with particle volume. This is consistent with the well-established volume scaling of biexciton lifetimes for a variety of direct and indirect-band-gap semiconductors.^11^ The linear volume dependence holds regardless of gallium content (Figure S26). It appears that for samples displaying clearly direct-like behavior (*i.e.*, low gallium content), Auger recombination dynamics are unaffected by alloying.

The observed Auger recombination behavior has important implications for the design of semiconductor nanocrystals for light emission applications at high powers. For applications such as color converters for micro-LEDs, active materials in quantum dot LEDs, and lasers, the non-radiative Auger recombination of bi- and multiexciton states can limit device performance at high power. Extending the biexciton lifetime is thus a key engineering goal for materials design. In a binary semiconductor, the emission wavelength can be tuned by size, where smaller particles are necessary to achieve bluer emission wavelengths. An unfortunate corollary of this is that smaller particles have shorter biexciton lifetimes due to the volume scaling of Auger recombination. This would be particularly acute for green-emitting InP nanocrystals, which require particles with a ∼2 nm diameter to achieve green emission.^26^ Alloying with a component that increases the band gap of the material would enable green emission from a particle with a larger diameter. This is clearly seen in the cases of InP and In_1–*x*_Ga*_x_*P, where green emission for InP requires particles with a ∼2 nm diameter but can be achieved by 3.7 nm diameter In_1–*x*_Ga*_x_*P QDs with 57% gallium incorporation. The biexciton lifetime we measure for our large-size green-emitting In_1–*x*_Ga*_x_*P/ZnS QDs is ∼40 ps, compared to the expected biexciton lifetime for green-emitting InP/ZnS of ∼2.2 ps based on extrapolation from experimental data on larger nanocrystals using volume scaling arguments. Our results show more generally that alloying can decouple the size/wavelength/biexciton lifetime relationship for semiconductor QDs and provide an additional parameter to engineer bright emitters for high-power applications.

For 4.9 nm diameter In_1–*x*_Ga*_x_*P QDs, we clearly traverse a direct-to-indirect-like transition at ∼65% gallium content. We measure the biexciton lifetime for these samples using the PIA signal (Figure S27) since there is no bleach signal in the indirect-like samples (Figure 9E). For the sample with 38% gallium, we measure a 40 ps lifetime. For 65–91% gallium content, we measure a biexciton lifetime of 70–80 ps. This represents a significant increase, but it is still far from the orders-of-magnitude difference expected if four-particle phonon-assisted models need to be invoked as in bulk semiconductors.^11^ Nonetheless, our results indicate that the direct-like or indirect-like nature of the first exciton can modulate the biexciton lifetimes of materials, which cannot be explained by simple volume scaling arguments.

We also calculated the effective Auger constants for our samples, which allow for the comparison of materials with varying sizes and compositions with their bulk counterparts. In Figure 9F, we show our samples (solid data point symbols) compared to several previously studied colloidal nanocrystals (open data point symbols). Our results fall within the data spread, which has been observed for II–VI and IV–VI materials already studied.^11^ This indicates that alloying does little to affect the biexciton lifetimes of colloidal nanocrystals, and volume scaling effects are the key factor for determining biexciton lifetimes. Our results here provide considerable guidance for further engineering long biexciton lifetimes in III–V/II–VI heterostructures. Alloying with a wider band gap material to achieve large cores with a given emission energy, combined with other strategies, such as graded alloy shells^86^ and quasi type-II band alignments,^87^ should enable synergistic improvement of optical properties.

### PL Characteristics at Elevated Temperatures

**Figure 10:**
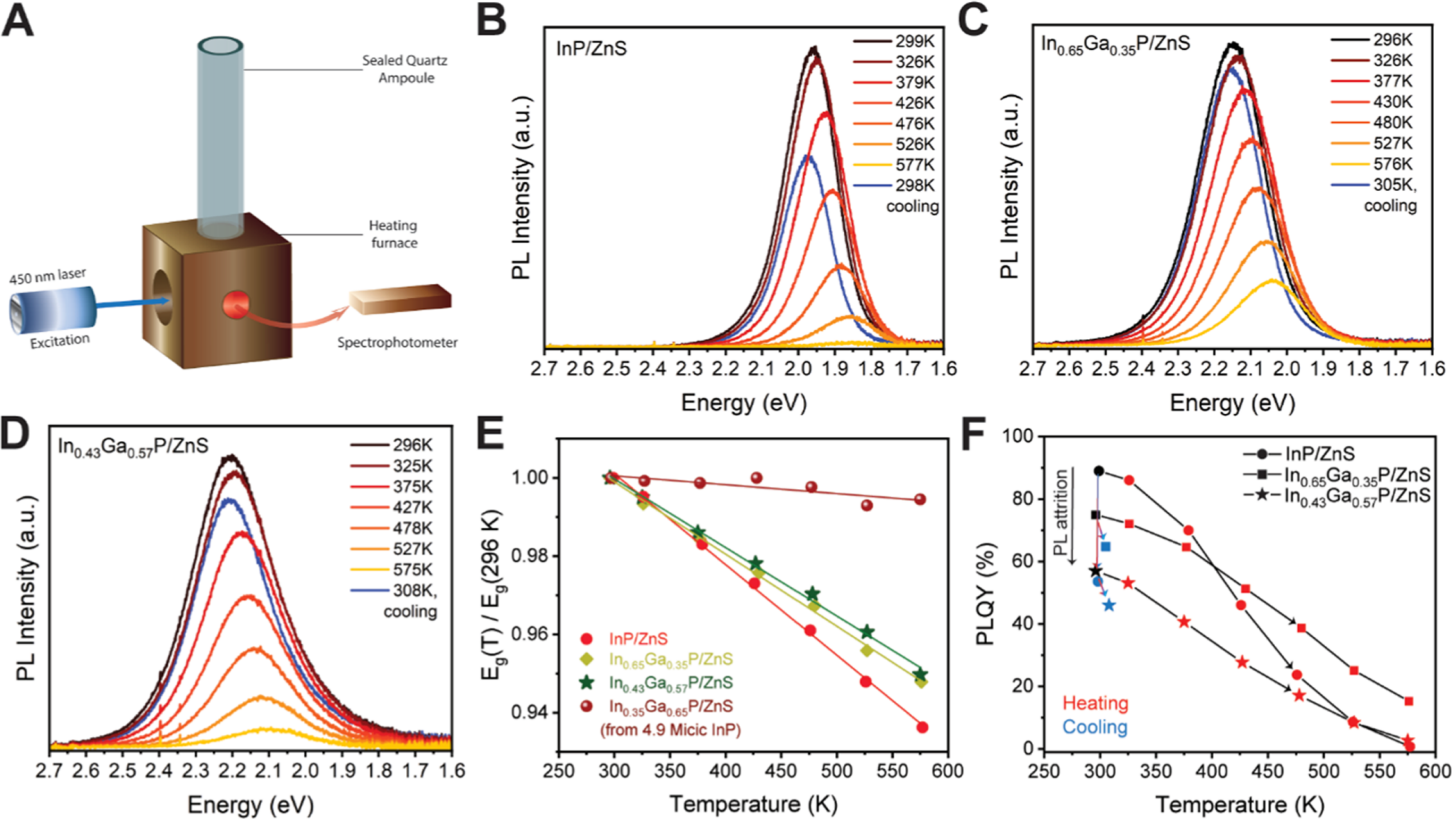
(A) Scheme of a home-built setup for investigating the high-temperature PL characteristics of In_1–*x*_Ga*_x_*P/ZnS QDs. Evolution of PL spectra as a function of temperature in emissive core–shell (B) InP/ZnS, (C) In_0.65_Ga_0.35_P/ZnS, and (D) In_0.43_Ga_0.57_P/ZnS QD samples, all derived from the 4 nm InP. (E) A Varshni plot (normalized at the room-temperature band gap for all samples) exhibits linear trends in the evolution of the optical band gap with temperature. (F) Changes in PLQY as a function of temperature were estimated as fractional changes in the integrated PL intensity.

When used for on-chip color conversion or incorporated into light-emitting diodes (LED), emissive materials often undergo significant thermal cycling in display panels. We employed a home-built setup to explore the PL characteristics of our emissive core–shell In_1–*x*_Ga*_x_*P/ZnS samples at elevated temperatures in solution, a schematic of which is presented in Figure 10A. A dilute solution of QDs in 1-octadecene was sealed inside a quartz ampoule using an oxy-hydrogen torch and excited using a 450 nm laser; the PL spectra were recorded using a fiber-coupled spectrophotometer collecting emission at 90 degrees with respect to the excitation beam. The sealed ampoule was placed inside a heating block, allowing us to raise the temperature externally. Figure 10B–D, respectively, illustrates the temperature-dependent PL spectra of the bright InP/ZnS, In_0.65_Ga_0.35_P/ZnS, and In_0.43_Ga_0.57_P/ZnS QD samples between ∼296 and 577 K, all derived from the 4 nm InP population. Minimal changes of the emission wavelength as a function of temperature are highly desirable to retain a consistent display color gamut and other characteristics of optoelectronic devices utilizing QD materials. To this end, we can empirically establish a Varshni relation for emissive samples by exploring the dependence of the band gap on temperature.^88^ A first-order high-temperature approximation of the semi-empirical Varshni relation, *E*_g_(*T*) = *E*_g_(296 K) – α*T*, allows us to employ linear fits to quantify the temperature dependence of the band gap of the In_1–*x*_Ga*_x_*P/ZnS QD samples. Lattice expansion at elevated temperatures contributes to a reduction of the direct band gap between the Γ-valley of the conduction band and the top of the valence band, evident in the associated Varshni plot (Figures 10E and S28). However, this trend is the opposite for the indirect gap between the Γ-valley of the valence band and the X-valley of the conduction band, where the band gap increases with temperature, thus counteracting the previous effect. Indeed, the linear fits reveal that the dependence of the band gap on temperature is weaker for the In_1–*x*_Ga*_x_*P/ZnS samples with higher Ga content. We attribute this observation to a mutual compensation of temperature effects for states with direct and indirect character and a larger contribution from the X-valley indirect-like states in samples with a higher Ga content, consistent with the previously described trends in the optoelectronic properties.

We also performed temperature-dependent PL studies on emissive samples derived from the large 4.9 nm InP nanocrystals, the results of which are summarized in Figure S29. For the compositions close to the direct-to-indirect transition, such as In_0.35_Ga_0.65_P/ZnS, the emission wavelength becomes nearly temperature-independent between 296 and 575 K (Figure 10E). Promisingly, these observations indicate that the alloyed nanocrystals retain their emission color gamut more efficiently than their binary counterparts at high temperatures.

We used the ratio of integrated PL intensities at ambient and elevated temperatures recorded in otherwise similar conditions to estimate the PLQY of emissive samples at elevated temperatures. The reduction in PLQY at elevated temperatures is typically attributed to thermally activated trapping of photoexcited carriers,^89−91^ whereas PL attrition post-annealing is ascribed to the surface reorganization and/or irreversible loss of ligands from the nanocrystal surface at higher temperatures.^92,93^ We measured the room-temperature PLQY of the InP/ZnS and a heavily optimized proprietary InP/ZnSe/ZnS sample to be 89 and 93%, respectively, using an integrating sphere setup (Figure S30). The trends in PLQY are summarized in Figure 10F for clarity. We observe a steeper decline in PLQY of the InP/ZnS sample at higher temperatures as well as a poorer PL recovery after cooling as compared to its cation-exchanged In_1–*x*_Ga*_x_*P counterparts, indicating that the alloy samples provide better high-temperature performance. As observed via TEM, the same reaction conditions allow for the growth of only one monolayer of the ZnS shell on InP nanocrystals (Figure S31). The substantial lattice mismatch between InP and ZnS (7.5%) likely prevents the shell from growing any thicker. Additionally, persistent interfacial strain at the core–shell interface can adversely affect optical performance via the propagation of defects.^35^ To test this hypothesis, we further performed temperature-dependent PL studies on the heavily optimized InP/ZnSe/ZnS commercial core–shell sample. The ZnSe shell has a lattice constant (0.5668 nm) between that of InP (0.5869 nm)^94^ and ZnS (0.5441 nm), thereby reducing interfacial strain from lattice mismatch, resulting in a near-perfect PLQY recovery post-annealing (Figure S32). This set of experiments reaffirms the relevance of In-to-Ga cation exchange as an important tool for reducing strain at the core–shell interface.

## Conclusions

To summarize, we have demonstrated a molten-salt-based In-to-Ga cation exchange and a subsequent oxygen-free shelling protocol to yield highly emissive In_1–*x*_Ga*_x_*P/ZnS colloidal QDs. Solid-state NMR studies show that the extent of oxidation of the core phosphide phase is negligible and limited to the surface. There is no discernible signature of the formation of segregated In and Ga domains, e.g., InP/GaP core–shells, suggesting that the alloy profile across the breadth of the core is homogeneous. We have performed transient absorption spectroscopy and photoluminescence studies, which demonstrate that the incorporation of gallium is accompanied by a decrease in oscillator strength of excitonic transitions, as indicated by a decreasing absorption cross-section and a monotonic increase in the radiative lifetime of emission. Atomistic calculations agree well with experimental observations and confirm the trends of optical properties as gallium composition changes in the alloyed QDs. The calculations provide direct access to the change in the degeneracies and wavefunction symmetry at the conduction band edge as the content of Ga is varied and provides further evidence for the direct-like to indirect-like transition at around 65% gallium composition. These results inform us that incorporating more gallium in In_1–*x*_Ga*_x_*P/ZnS CQDs can only be part of a strategy to prepare blue III-P emitters since high gallium contents result in low oscillator strength of emissive states. We further demonstrate that volume scaling effects, instead of composition, predominantly determine the biexciton lifetimes in these alloyed nanocrystals, a promising indication that deleterious Auger processes are slower in In_1–*x*_Ga*_x_*P/ZnS CQDs as compared to their smaller InP-based counterpart emitting at similar wavelengths. Compared to InP, a better lattice match between In_1–*x*_Ga*_x_*P and the ZnS shell allows for better PLQY recovery during thermal cycling. These results establish molten-salt-synthesized In_1–*x*_Ga*_x_*P nanocrystals as a viable alternative to InP for designing next-generation III–V QDs for displays and other applications. Further, we establish that alloying can decouple mutually size-dependent properties, enabling flexible engineering of emitters with desired properties.

## Supporting Information Available

Additional absorption and emission spectra; time-resolved photoluminescence decay profiles; X-ray diffraction patterns and corresponding peak shifts; SAXS data and photographs of the experimental setup (PDF)

## References

[ref1] Ashcroft N. W.; Mermin N. D. Solid State Physics; W. B. Saunders Company, 1976; pp 131–173.

[ref2] Brus L. E. Electron–electron and electron-hole interactions in small semiconductor crystallites: The size dependence of the lowest excited electronic state. J. Chem. Phys. 1984, 80, 4403–4409. 10.1063/1.447218.

[ref3] Schaller R. D.; Klimov V. I. High Efficiency Carrier Multiplication in PbSe Nanocrystals: Implications for Solar Energy Conversion. Phys. Rev. Lett. 2004, 92, 186601 10.1103/PhysRevLett.92.186601. 15169518

[ref4] Ellingson R. J.; Beard M. C.; Johnson J. C.; Yu P.; Micic O. I.; Nozik A. J.; Shabaev A.; Efros A. L. Highly Efficient Multiple Exciton Generation in Colloidal PbSe and PbS Quantum Dots. Nano Lett. 2005, 5, 865–871. 10.1021/nl0502672. 15884885

[ref5] Efros A. L.; Rosen M. The Electronic Structure of Semiconductor Nanocrystals. Annu. Rev. Mater. Sci. 2000, 30, 475–521. 10.1146/annurev.matsci.30.1.475.

[ref6] Chang A. Y.; Liu W.; Talapin D. V.; Schaller R. D. Carrier Dynamics in Highly Quantum-Confined, Colloidal Indium Antimonide Nanocrystals. ACS Nano 2014, 8, 8513–8519. 10.1021/nn5031274. 25106893

[ref7] Murray C. B.; Norris D. J.; Bawendi M. G. Synthesis and characterization of nearly monodisperse CdE (E = sulfur, selenium, tellurium) semiconductor nanocrystallites. J. Am. Chem. Soc. 1993, 115, 8706–8715. 10.1021/ja00072a025.

[ref8] Balan A. D.; Eshet H.; Olshansky J. H.; Lee Y. V.; Rabani E.; Alivisatos A. P. Effect of Thermal Fluctuations on the Radiative Rate in Core/Shell Quantum Dots. Nano Lett. 2017, 17, 1629–1636. 10.1021/acs.nanolett.6b04816. 28183177

[ref9] Efros A. L.; Brus L. E. Nanocrystal Quantum Dots: From Discovery to Modern Development. ACS Nano 2021, 15, 6192–6210. 10.1021/acsnano.1c01399. 33830732

[ref10] Klimov V. I.; Schwarz C. J.; McBranch D. W.; Leatherdale C. A.; Bawendi M. G. Ultrafast dynamics of inter- and intraband transitions in semiconductor nanocrystals: Implications for quantum-dot lasers. Phys. Rev. B 1999, 60, R2177–R2180. 10.1103/PhysRevB.60.R2177.

[ref11] Robel I.; Gresback R.; Kortshagen U.; Schaller R. D.; Klimov V. I. Universal Size-Dependent Trend in Auger Recombination in Direct-Gap and Indirect-Gap Semiconductor Nanocrystals. Phys. Rev. Lett. 2009, 102, 177404 10.1103/PhysRevLett.102.177404. 19518831

[ref12] Carroll G. M.; Limpens R.; Neale N. R. Tuning Confinement in Colloidal Silicon Nanocrystals with Saturated Surface Ligands. Nano Lett. 2018, 18, 3118–3124. 10.1021/acs.nanolett.8b00680. 29659285

[ref13] Klimov V. I.; Schwarz C. J.; McBranch D. W.; White C. W. Initial carrier relaxation dynamics in ion-implanted Si nanocrystals: Femtosecond transient absorption study. Appl. Phys. Lett. 1998, 73, 2603–2605. 10.1063/1.122519.

[ref14] Hybertsen M. S. Absorption and emission of light in nanoscale silicon structures. Phys. Rev. Lett. 1994, 72, 1514–1517. 10.1103/PhysRevLett.72.1514. 10055628

[ref15] Kovalev D.; Heckler H.; Ben-Chorin M.; Polisski G.; Schwartzkopff M.; Koch F. Breakdown of the k-Conservation Rule in Si Nanocrystals. Phys. Rev. Lett. 1998, 81, 2803–2806. 10.1103/PhysRevLett.81.2803.

[ref16] Onton A.; Lorenz M. R.; Reuter W. Electronic Structure and Luminescence Processes in In_1–x_Ga_x_P Alloys. J. Appl. Phys. 1971, 42, 3420–3432. 10.1063/1.1660748.

[ref17] James L. W.; Van Dyke J. P.; Herman F.; Chang D. M. Band Structure and High-Field Transport Properties of InP. Phys. Rev. B 1970, 1, 3998–4004. 10.1103/PhysRevB.1.3998.

[ref18] Williams G. P.; Cerrina F.; Lapeyre G. J.; Anderson J. R.; Smith R. J.; Hermanson J. Experimental study of the band structure of GaP, GaAs, GaSb, InP, InAs, and InSb. Phys. Rev. B 1986, 34, 5548–5557. 10.1103/PhysRevB.34.5548. 9940388

[ref19] Onton A.; Chicotka R. J. Photoluminescence Processes in In_1–x_Ga_x_P at 2°K. Phys. Rev. B 1971, 4, 1847–1853. 10.1103/PhysRevB.4.1847.

[ref20] Lee J.-W.; Schremer A. T.; Fekete D.; Shealy J. R.; Ballantyne J. M. Growth of direct bandgap GalnP quantum dots on GaP substrates. J. Electron. Mater. 1997, 26, 1199–1204. 10.1007/s11664-997-0020-0.

[ref21] Gao H.; Sun Q.; Sun W.; Tan H. H.; Jagadish C.; Zou J. Understanding the Effect of Catalyst Size on the Epitaxial Growth of Hierarchical Structured InGaP Nanowires. Nano Lett. 2019, 19, 8262–8269. 10.1021/acs.nanolett.9b03835. 31661618

[ref22] Gao H.; Sun W.; Sun Q.; Tan H. H.; Jagadish C.; Zou J. Compositional Varied Core–Shell InGaP Nanowires Grown by Metal–Organic Chemical Vapor Deposition. Nano Lett. 2019, 19, 3782–3788. 10.1021/acs.nanolett.9b00915. 31117755

[ref23] Kornienko N.; Whitmore D. D.; Yu Y.; Leone S. R.; Yang P. Solution Phase Synthesis of Indium Gallium Phosphide Alloy Nanowires. ACS Nano 2015, 9, 3951–3960. 10.1021/nn507335j. 25839336

[ref24] Srivastava V.; Liu W.; Janke E. M.; Kamysbayev V.; Filatov A. S.; Sun C.-J.; Lee B.; Rajh T.; Schaller R. D.; Talapin D. V. Understanding and Curing Structural Defects in Colloidal GaAs Nanocrystals. Nano Lett. 2017, 17, 2094–2101. 10.1021/acs.nanolett.7b00481. 28191964

[ref25] Li Y.; Hou X.; Dai X.; Yao Z.; Lv L.; Jin Y.; Peng X. Stoichiometry-Controlled InP-Based Quantum Dots: Synthesis, Photoluminescence, and Electroluminescence. J. Am. Chem. Soc. 2019, 141, 6448–6452. 10.1021/jacs.8b12908. 30964282

[ref26] Kim Y.; Ham S.; Jang H.; Min J. H.; Chung H.; Lee J.; Kim D.; Jang E. Bright and Uniform Green Light Emitting InP/ZnSe/ZnS Quantum Dots for Wide Color Gamut Displays. ACS Appl. Nano Mater. 2019, 2, 1496–1504. 10.1021/acsanm.8b02063.

[ref27] Won Y.-H.; Cho O.; Kim T.; Chung D.-Y.; Kim T.; Chung H.; Jang H.; Lee J.; Kim D.; Jang E. Highly efficient and stable InP/ZnSe/ZnS quantum dot light-emitting diodes. Nature 2019, 575, 634–638. 10.1038/s41586-019-1771-5. 31776489

[ref28] Ramasamy P.; Ko K.-J.; Kang J.-W.; Lee J.-S. Two-Step “Seed-Mediated” Synthetic Approach to Colloidal Indium Phosphide Quantum Dots with High-Purity Photo- and Electroluminescence. Chem. Mater. 2018, 30, 3643–3647. 10.1021/acs.chemmater.8b02049.

[ref29] Kim Y.-H.; Jun Y.-w.; Jun B.-H.; Lee S.-M.; Cheon J. Sterically Induced Shape and Crystalline Phase Control of GaP Nanocrystals. J. Am. Chem. Soc. 2002, 124, 13656–13657. 10.1021/ja027575b. 12431078

[ref30] Micic O. I.; Sprague J. R.; Curtis C. J.; Jones K. M.; Machol J. L.; Nozik A. J.; Giessen H.; Fluegel B.; Mohs G.; Peyghambarian N. Synthesis and Characterization of InP, GaP, and GaInP_2_ Quantum Dots. J. Phys. Chem. A 1995, 99, 7754–7759. 10.1021/j100019a063.

[ref31] Kim K.-H.; Jo J.-H.; Jo D.-Y.; Han C.-Y.; Yoon S.-Y.; Kim Y.; Kim Y.-H.; Ko Y. H.; Kim S. W.; Lee C.; Yang H. Cation-Exchange-Derived InGaP Alloy Quantum Dots toward Blue Emissivity. Chem. Mater. 2020, 32, 3537–3544. 10.1021/acs.chemmater.0c00551.

[ref32] Jeong B. G.; Chang J. H.; Hahm D.; Rhee S.; Park M.; Lee S.; Kim Y.; Shin D.; Park J. W.; Lee C.; Lee D. C.; Park K.; Hwang E.; Bae W. K. Interface polarization in heterovalent core–shell nanocrystals. Nat. Mater. 2022, 21, 246–252. 10.1038/s41563-021-01119-8. 34795403

[ref33] Zhang H.; Dasbiswas K.; Ludwig N. B.; Han G.; Lee B.; Vaikuntanathan S.; Talapin D. V. Stable colloids in molten inorganic salts. Nature 2017, 542, 328–331. 10.1038/nature21041. 28202966

[ref34] Kamysbayev V.; Srivastava V.; Ludwig N. B.; Borkiewicz O. J.; Zhang H.; Ilavsky J.; Lee B.; Chapman K. W.; Vaikuntanathan S.; Talapin D. V. Nanocrystals in Molten Salts and Ionic Liquids: Experimental Observation of Ionic Correlations Extending beyond the Debye Length. ACS Nano 2019, 13, 5760–5770. 10.1021/acsnano.9b01292. 30964280

[ref35] Srivastava V.; Kamysbayev V.; Hong L.; Dunietz E.; Klie R. F.; Talapin D. V. Colloidal Chemistry in Molten Salts: Synthesis of Luminescent In_1–x_Ga_x_P and In_1–x_Ga_x_As Quantum Dots. J. Am. Chem. Soc. 2018, 140, 12144–12151. 10.1021/jacs.8b06971. 30125092

[ref36] Hudson M. H.; Gupta A.; Srivastava V.; Janke E. M.; Talapin D. V. Synthesis of In_1–x_Ga_x_P Quantum Dots in Lewis Basic Molten Salts: The Effects of Surface Chemistry, Reaction Conditions, and Molten Salt Composition. J. Phys. Chem. C 2022, 126, 1564–1580. 10.1021/acs.jpcc.1c10394.

[ref37] Gupta A.; Ondry J. C.; Chen M.; Hudson M. H.; Coropceanu I.; Sarma N. A.; Talapin D. V. Diffusion-Limited Kinetics of Isovalent Cation Exchange in III–V Nanocrystals Dispersed in Molten Salt Reaction Media. Nano Lett. 2022, 22, 6545–6552. 10.1021/acs.nanolett.2c01699. 35952655 PMC9413424

[ref38] Mićić O. I.; Nozik A. J.; Lifshitz E.; Rajh T.; Poluektov O. G.; Thurnauer M. C. Electron and Hole Adducts Formed in Illuminated InP Colloidal Quantum Dots Studied by Electron Paramagnetic Resonance. J. Phys. Chem. B 2002, 106, 4390–4395. 10.1021/jp014180q.

[ref39] Ilavsky J.; Jemian P. R. Irena: Tool Suite for Modeling and Analysis of Small-Angle Scattering. J. Appl. Crystallogr. 2009, 42, 347–353. 10.1107/S0021889809002222.

[ref40] Kroupa D. M.; Voros M.; Brawand N. P.; McNichols B. W.; Miller E. M.; Gu J.; Nozik A. J.; Sellinger A.; Galli G.; Beard M. C. Tuning colloidal quantum dot band edge positions through solution-phase surface chemistry modification. Nat. Commun. 2017, 8, 15257 10.1038/ncomms15257. 28508866 PMC5440806

[ref41] Yang Y.; Qin H.; Jiang M.; Lin L.; Fu T.; Dai X.; Zhang Z.; Niu Y.; Cao H.; Jin Y.; Zhao F.; Peng X. Entropic Ligands for Nanocrystals: From Unexpected Solution Properties to Outstanding Processability. Nano Lett. 2016, 16, 2133–2138. 10.1021/acs.nanolett.6b00730. 26923682

[ref42] Calvin J. J.; Kaufman T. M.; Sedlak A. B.; Crook M. F.; Alivisatos A. P. Observation of ordered organic capping ligands on semiconducting quantum dots via powder X-ray diffraction. Nat. Commun. 2021, 12, 2663 10.1038/s41467-021-22947-x. 33976186 PMC8113276

[ref43] Cros-Gagneux A.; Delpech F.; Nayral C.; Cornejo A.; Coppel Y.; Chaudret B. Surface chemistry of InP quantum dots: a comprehensive study. J. Am. Chem. Soc. 2010, 132, 18147–18157. 10.1021/ja104673y. 21126088

[ref44] Tessier M. D.; Baquero E. A.; Dupont D.; Grigel V.; Bladt E.; Bals S.; Coppel Y.; Hens Z.; Nayral C.; Delpech F. Interfacial Oxidation and Photoluminescence of InP-Based Core/Shell Quantum Dots. Chem. Mater. 2018, 30, 6877–6883. 10.1021/acs.chemmater.8b03117.

[ref45] Hanrahan M. P.; Stein J. L.; Park N.; Cossairt B. M.; Rossini A. J. Elucidating the Location of Cd^2+^ in Post-synthetically Treated InP Quantum Dots Using Dynamic Nuclear Polarization ^31^P and ^113^Cd Solid-State NMR Spectroscopy. J. Phys. Chem. C 2021, 125, 2956–2965. 10.1021/acs.jpcc.0c09601.

[ref46] Tomaselli M.; Yarger J. L.; Bruchez M.; Havlin R. H.; deGraw D.; Pines A.; Alivisatos A. P. NMR study of InP quantum dots: Surface structure and size effects. J. Chem. Phys. 1999, 110, 8861–8864. 10.1063/1.478858.

[ref47] Lütgemeier H. Die chemische Verschiebung der Kernresonanzlinien in A(III)B(V)-Verbindungen. Z. Naturforsch., A 1964, 19, 1297–1300. 10.1515/zna-1964-1106.

[ref48] Duncan T. M.; Karlicek R. F.; Bonner W. A.; Thiel F. A. A ^31^P Nuclear Magnetic Resonance study of InP, GaP and InGaP. J. Phys. Chem. Solids 1984, 45, 389–391. 10.1016/0022-3697(84)90145-8.

[ref49] Tycko R.; Dabbagh G.; Kurtz S. R.; Goral J. P. Quantitative study of atomic ordering in Ga_0.5_In_0.5_P thin films by ^31^P nuclear magnetic resonance. Phys. Rev. B 1992, 45, 13452–13457. 10.1103/PhysRevB.45.13452. 10001431

[ref50] Knijn P. J.; van Bentum P. J. M.; Fang C. M.; Bauhuis G. J.; de Wijs G. A.; Kentgens A. P. M. A multi-nuclear magnetic resonance and density functional theory investigation of epitaxially grown InGaP_2_. Phys. Chem. Chem. Phys. 2016, 18, 21296–21304. 10.1039/C5CP04593B. 27424548

[ref51] Tomaselli M.; deGraw D.; Yarger J. L.; Augustine M. P.; Pines A. Scalar and anisotropic J interactions in undoped InP: A triple-resonance NMR study. Phys. Rev. B 1998, 58, 8627–8633. 10.1103/PhysRevB.58.8627.

[ref52] Adamová G.; Gardas R. L.; Rebelo L. P. N.; Robertson A. J.; Seddon K. R. Alkyltrioctylphosphonium chloride ionic liquids: synthesis and physicochemical properties. Dalton Trans. 2011, 40, 12750–12764. 10.1039/C1DT10332F. 21996935

[ref53] Thomas L. C. The Identification of Functional Groups in Organophosphorus Compounds; Academic Press: New York, 1974; pp 5–121.

[ref54] Crutchfield M. M.; Griffith E. J.; Grayson M. Topics in Phosphorus Chemistry; Interscience Publishers: New York, 1967; Vol. 5.

[ref55] Tessier M. D.; De Nolf K.; Dupont D.; Sinnaeve D.; De Roo J.; Hens Z. Aminophosphines: A Double Role in the Synthesis of Colloidal Indium Phosphide Quantum Dots. J. Am. Chem. Soc. 2016, 138, 5923–5929. 10.1021/jacs.6b01254. 27111735

[ref56] Virieux H.; Le Troedec M.; Cros-Gagneux A.; Ojo W.-S.; Delpech F.; Nayral C.; Martinez H.; Chaudret B. InP/ZnS Nanocrystals: Coupling NMR and XPS for Fine Surface and Interface Description. J. Am. Chem. Soc. 2012, 134, 19701–19708. 10.1021/ja307124m. 23131073

[ref57] Kemp T. F.; Smith M. E. QuadFit—A new cross-platform computer program for simulation of NMR line shapes from solids with distributions of interaction parameters. Solid State Nucl. Magn. Reson. 2009, 35, 243–252. 10.1016/j.ssnmr.2008.12.003. 19186033

[ref58] Neuville D. R.; Cormier L.; Massiot D. Al environment in tectosilicate and peraluminous glasses: A ^27^Al MQ-MAS NMR, Raman, and XANES investigation. Geochim. Cosmochim. Acta 2004, 68, 5071–5079. 10.1016/j.gca.2004.05.048.

[ref59] d’Espinose de Lacaillerie J.-B.; Fretigny C.; Massiot D. MAS NMR spectra of quadrupolar nuclei in disordered solids: The Czjzek model. J. Magn. Reson. 2008, 192, 244–251. 10.1016/j.jmr.2008.03.001. 18362082

[ref60] Werner-Zwanziger U.; Paterson A. L.; Zwanziger J. W. The Czjzek distribution in solid-state NMR: Scaling properties of central and satellite transitions. J. Non-Cryst. Solids 2020, 550, 120383 10.1016/j.jnoncrysol.2020.120383.

[ref61] Kempgens P. Semi-analytical description of the S = 9/2 quadrupole nutation NMR experiment: multinuclear application to 113In and 115In in indium phosphide. Magn. Reson. Chem. 2015, 53, 261–266. 10.1002/mrc.4186. 25616015

[ref62] van Meerten S. G. J.; Franssen W. M. J.; Kentgens A. P. M. ssNake: A cross-platform open-source NMR data processing and fitting application. J. Magn. Reson. 2019, 301, 56–66. 10.1016/j.jmr.2019.02.006. 30851666

[ref63] Adolphi N. L.; Conradi M. S.; Buhro W. E. The ^31^P NMR spectrum of InP. J. Phys. Chem. Solids 1992, 53, 1073–1074. 10.1016/0022-3697(92)90080-W.

[ref64] Sears R. E. J.; Ni Q. W. ^115^In hexadecapole interaction upper limit in InP: ^31^P–^115^In exchange and pseudodipolar coupling constants. J. Chem. Phys. 1994, 100, 4708–4709. 10.1063/1.466258.

[ref65] Wasylishen R. E.; Wright K. C.; Eichele K.; Cameron T. S. Characterization of the J(indium-115, phosphorus-31) tensor for a 1:1 adduct of indium tribromide and a triarylphosphine. Inorg. Chem. 1994, 33, 407–408. 10.1021/ic00081a003.

[ref66] Chen F.; Ma G.; Bernard G. M.; Cavell R. G.; McDonald R.; Ferguson M. J.; Wasylishen R. E. Solid-State ^115^In and ^31^P NMR Studies of Triarylphosphine Indium Trihalide Adducts. J. Am. Chem. Soc. 2010, 132, 5479–5493. 10.1021/ja100625p. 20349956

[ref67] Iijima T.; Hashi K.; Goto A.; Shimizu T.; Ohki S. Anisotropic indirect nuclear spin–spin coupling in InP: ^31^P CP NMR study under slow MAS condition. Chem. Phys. Lett. 2006, 419, 28–32. 10.1016/j.cplett.2005.11.049.

[ref68] Iijima T.; Hashi K.; Goto A.; Shimizu T.; Ohki S. Indirect nuclear spin–spin coupling in InP studied by CP/MAS NMR. Phys. B 2004, 346–347, 476–478. 10.1016/j.physb.2004.01.130.

[ref69] Wang L.-W.; Zunger A. Pseudopotential calculations of nanoscale CdSe quantum dots. Phys. Rev. B 1996, 53, 9579–9582. 10.1103/PhysRevB.53.9579. 9982505

[ref70] Wang L.-W.; Zunger A. Local-density-derived semiempirical pseudopotentials. Phys. Rev. B 1995, 51, 17398–17416. 10.1103/PhysRevB.51.17398. 9978766

[ref71] Enright M. J.; Jasrasaria D.; Hanchard M. M.; Needell D. R.; Phelan M. E.; Weinberg D.; McDowell B. E.; Hsiao H.-W.; Akbari H.; Kottwitz M.; Potter M. M.; Wong J.; Zuo J.-M.; Atwater H. A.; Rabani E.; Nuzzo R. G. Role of Atomic Structure on Exciton Dynamics and Photoluminescence in NIR Emissive InAs/InP/ZnSe Quantum Dots. J. Phys. Chem. C 2022, 126, 7576–7587. 10.1021/acs.jpcc.2c01499.

[ref72] Rabani E.; Hetényi B.; Berne B. J.; Brus L. E. Electronic properties of CdSe nanocrystals in the absence and presence of a dielectric medium. J. Chem. Phys. 1999, 110, 5355–5369. 10.1063/1.478431.

[ref73] Eshet H.; Grünwald M.; Rabani E. The Electronic Structure of CdSe/CdS Core/Shell Seeded Nanorods: Type-I or Quasi-Type-II?. Nano Lett. 2013, 13, 5880–5885. 10.1021/nl402722n. 24215466 PMC3862461

[ref74] Rohlfing M.; Louie S. G. Electron-hole excitations and optical spectra from first principles. Phys. Rev. B 2000, 62, 4927–4944. 10.1103/PhysRevB.62.4927.

[ref75] Jasrasaria D.; Weinberg D.; Philbin J. P.; Rabani E. Simulations of nonradiative processes in semiconductor nanocrystals. J. Chem. Phys. 2022, 157, 020901 10.1063/5.0095897. 35840368

[ref76] Powell D.; Migliorato M. A.; Cullis A. G. Optimized Tersoff potential parameters for tetrahedrally bonded III-V semiconductors. Phys. Rev. B 2007, 75, 115202 10.1103/PhysRevB.75.115202.

[ref77] Klimov V. I. Optical Nonlinearities and Ultrafast Carrier Dynamics in Semiconductor Nanocrystals. J. Phys. Chem. B 2000, 104, 6112–6123. 10.1021/jp9944132.

[ref78] Mourad D.; Guille A.; Aubert T.; Brainis E.; Hens Z. Random-Alloying Induced Signatures in the Absorption Spectra of Colloidal Quantum Dots. Chem. Mater. 2014, 26, 6852–6862. 10.1021/cm5035408.

[ref79] Mlinar V.; Zunger A. Effect of atomic-scale randomness on the optical polarization of semiconductor quantum dots. Phys. Rev. B 2009, 79, 115416 10.1103/PhysRevB.79.115416.

[ref80] Corney A. The Spontaneous Radiation of Emission. Atomic and Laser Spectroscopy; Clarendon Press: Oxford, Great Britain, 1978; pp 93–115.

[ref81] Jasieniak J.; Smith L.; van Embden J.; Mulvaney P.; Califano M. Re-examination of the Size-Dependent Absorption Properties of CdSe Quantum Dots. J. Phys. Chem. C 2009, 113, 19468–19474. 10.1021/jp906827m.

[ref82] Makuła P.; Pacia M.; Macyk W. How To Correctly Determine the Band Gap Energy of Modified Semiconductor Photocatalysts Based on UV–Vis Spectra. J. Phys. Chem. Lett. 2018, 9, 6814–6817. 10.1021/acs.jpclett.8b02892. 30990726

[ref83] Jasrasaria D.; Philbin J. P.; Yan C.; Weinberg D.; Alivisatos A. P.; Rabani E. Sub-Bandgap Photoinduced Transient Absorption Features in CdSe Nanostructures: The Role of Trapped Holes. J. Phys. Chem. C 2020, 124, 17372–17378. 10.1021/acs.jpcc.0c04746.

[ref84] Makarov N. S.; Guo S.; Isaienko O.; Liu W.; Robel I.; Klimov V. I. Spectral and Dynamical Properties of Single Excitons, Biexcitons, and Trions in Cesium–Lead-Halide Perovskite Quantum Dots. Nano Lett. 2016, 16, 2349–2362. 10.1021/acs.nanolett.5b05077. 26882294

[ref200] Luo J.-W.; Franceschetti A.; Zunger A. Quantum-Size-Induced Electronic Transitions in Quantum Dots: Indirect Band-Gap GaAs. Phys. Rev. B 2008, 78, 035306 10.1103/PhysRevB.78.035306.

[ref85] Klimov V. I. Multicarrier Interactions in Semiconductor Nanocrystals in Relation to the Phenomena of Auger Recombination and Carrier Multiplication. Annu. Rev. Condens. Matter Phys. 2014, 5, 285–316. 10.1146/annurev-conmatphys-031113-133900.

[ref86] Chen Y.; Vela J.; Htoon H.; Casson J. L.; Werder D. J.; Bussian D. A.; Klimov V. I.; Hollingsworth J. A. “Giant” Multishell CdSe Nanocrystal Quantum Dots with Suppressed Blinking. J. Am. Chem. Soc. 2008, 130, 5026–5027. 10.1021/ja711379k. 18355011

[ref87] Cragg G. E.; Efros A. L. Suppression of Auger Processes in Confined Structures. Nano Lett. 2010, 10, 313–317. 10.1021/nl903592h. 20017564

[ref88] Varshni Y. P. Temperature dependence of the energy gap in semiconductors. Physica 1967, 34, 149–154. 10.1016/0031-8914(67)90062-6.

[ref89] Rowland C. E.; Liu W.; Hannah D. C.; Chan M. K. Y.; Talapin D. V.; Schaller R. D. Thermal Stability of Colloidal InP Nanocrystals: Small Inorganic Ligands Boost High-Temperature Photoluminescence. ACS Nano 2014, 8, 977–985. 10.1021/nn405811p. 24328364

[ref90] Zhao Y.; Riemersma C.; Pietra F.; Koole R.; de Mello Donegá C.; Meijerink A. High-Temperature Luminescence Quenching of Colloidal Quantum Dots. ACS Nano 2012, 6, 9058–9067. 10.1021/nn303217q. 22978378

[ref91] Rowland C. E.; Fedin I.; Diroll B. T.; Liu Y.; Talapin D. V.; Schaller R. D. Elevated Temperature Photophysical Properties and Morphological Stability of CdSe and CdSe/CdS Nanoplatelets. J. Phys. Chem. Lett. 2018, 9, 286–293. 10.1021/acs.jpclett.7b02793. 29283580

[ref92] Porter V. J.; Geyer S.; Halpert J. E.; Kastner M. A.; Bawendi M. G. Photoconduction in Annealed and Chemically Treated CdSe/ZnS Inorganic Nanocrystal Films. J. Phys. Chem. C 2008, 112, 2308–2316. 10.1021/jp710173q.

[ref93] Talapin D. V.; Lee J.-S.; Kovalenko M. V.; Shevchenko E. V. Prospects of Colloidal Nanocrystals for Electronic and Optoelectronic Applications. Chem. Rev. 2010, 110, 389–458. 10.1021/cr900137k. 19958036

[ref94] Levinshtein M.; Rumyantsev S.; Shur M. Handbook Series on Semiconductor Parameters; World Scientific: London, 1996; Vol. 1, pp 169–190.

