# Supplementary material for: Composition-Defined Optical Properties and the Direct-to-Indirect Transition in Core–Shell In1–xGaxP/ZnS Colloidal Quantum Dots
Source: J Am Chem Soc. 2023 Jul 19;145(30):16429–48. doi: 10.1021/jacs.3c02709 (PMC10401719; doi:10.1021/jacs.3c02709)
Supplement: Supplementary file 1 — ja3c02709_si_001.pdf [file ja3c02709_si_001.pdf]

Supporting Information

Composition-Defined Optical Properties and the Direct-to-Indirect Transition in Core-Shell $In_{1-x}Ga_xP$/ZnS Colloidal Quantum Dots

Aritrajit Gupta,[1§] Justin C. Ondry,[1§] Kailai Lin,[3] Yunhua Chen,[4,5] Margaret H. Hudson,[1] Min Chen,[1] Richard D. Schaller,[2,6] Aaron J. Rossini[4,5], Eran Rabani[3,7,8], and Dmitri V. Talapin[1,2]*

[1]*Department of Chemistry, James Franck Institute, and Pritzker School of Molecular Engineering, University of Chicago, Chicago, Illinois 60637, United States*

[2]*Center for Nanoscale Materials, Argonne National Laboratory, Argonne, Illinois 60439, United States*

[3]*Department of Chemistry, University of California, Berkeley, California 94720, USA*

[4]*US DOE Ames Laboratory, Ames, Iowa 50011, United States*

[5]*Department of Chemistry, Iowa State University, Ames, Iowa 50011, United States*

[6]*Department of Chemistry, Northwestern University, Evanston, Illinois 60208, United States*

[7]*Materials Sciences Division, Lawrence Berkeley National Laboratory, Berkeley, California 94720, USA*

[8]*The Raymond and Beverly Sackler Center of Computational Molecular and Materials Science, Tel Aviv University, Tel Aviv 69978, Israel*

*E-mail: dvtalapin@uchicago.edu

[§]These authors contributed equally.

EXPERIMENTAL SECTION

Chemicals: Trioctylphosphine (TOP, 97%), trioctylphosphine oxide (TOPO, 99%), hexamethylphosphoramide (HMPA, 99%), myristic acid (99%), sulfur (99.998%) and anhydrous solvents (hexane, toluene, ethanol (EtOH), methyl acetate, isopropanol (IPA), acetonitrile (MeCN)) were purchased from Sigma Aldrich and used as received. Oleylamine (OAm, technical grade, 70%) was purchased from Sigma Aldrich and purified by freezing, thawing, and then centrifuging to remove any insoluble solids. The resulting purified oleylamine was dried under dynamic vacuum at 100°C overnight and stored in a nitrogen glovebox. 1-Octadecene (ODE, 90%) was purchased from Sigma Aldrich, dried under dynamic vacuum at 80°C overnight and stored in a nitrogen glovebox. Dodecylamine (98%) and octadecylamine (technical grade, 90%) were purchased from Sigma Aldrich and dried under dynamic vacuum before storage in a nitrogen glovebox. Potassium iodide (ultra-dry, 99.998%), gallium(iii) iodide (ultra-dry, 99.999%), indium(iii) acetate (99.99%), zinc chloride (ultra-dry, 99.999%) and N,N-dimethylformamide (DMF, anhydrous 99.9%) were purchased from Alfa Aesar and used as received. *Tris*(trimethylsilyl)phosphine ($(TMS)_3P$, 98%, stored frozen) and indium(iii) chloride (anhydrous, 99.999%) were purchased from Strem Chemicals and used as received.

InP nanocrystal synthesis:

Large InP QDs. Large InP nanocrystals (~4.9 nm diameter) were synthesized following a route established by Micic and others.[1-2] Briefly, 1 g $InCl_3$ (4.52 mmol), 1.5 g trioctylphosphine oxide (3.88 mmol) and 15 mL of trioctylphosphine were combined in a nitrogen glovebox and stirred at room temperature. After complete dissolution of the solids, the solution was filtered through a 0.2 µm PTFE filter into a three-neck flask, which was then sealed and transferred air-free to a Schlenk line under nitrogen purge. With the contents of the flask at 25°C, 0.75g (869 µL, 4 mmol) of tris(trimethylsilyl)phosphine was injected. The temperature of the flask was then raised to 270°C and maintained under stirring for 30 hours. Particles were washed under the inert atmosphere of a nitrogen filled glovebox by precipitation with ethanol followed by redispersal in toluene. After another step of precipitation/redispersal, the particles were size selectively precipitated from toluene by incremental addition of ethanol followed by centrifugation after each addition. The largest particles contained in the first fraction were used in further steps.

InP QDs of intermediate size. A consistent sample of nearly monodisperse InP nanocrystals, (4 nm diameter), was obtained from commercial sources as a solution in octadecene (ODE) and stored in a nitrogen glove box. The QDs were precipitated by addition of ethanol (EtOH), centrifuged, and redispersed in toluene for further processing.

Small InP QDs. The synthesis of small InP nanocrystals (~3.2 nm diameter) was adapted from a prior report by the Peng group.[3] 5 mmol $In(Ac)_3$, 15 mmol myristic acid and 35 mL octadecene were mixed in a 250 mL flask. After degassing for 1 hour under vacuum at 120 °C, 5 mL TOP was injected. Into this solution, a solution of 2.5 mmol $(TMS)_3P$ in ODE (8 mL) was injected at 150°C. The reaction temperature was next raised to 270 °C for growing the nanocrystals. After 5 minutes, the reaction mixture was cooled down, and the synthesized particles were washed three times using ethanol and methyl acetate with added trioctylphosphine ligands. Finally, the nanocrystals were stored as a colloidal solution in hexane.

GaI_3 ligand exchange. The GaI_3 ligand exchange was adapted from a previous publication.[4] A solution of as-synthesized InP in hexane (15 mL, 1 mg/mL) was layered atop a solution of GaI_3 in dimethylformamide (DMF) (2 mL, 0.05 M), and stirred until all particles transferred to the DMF phase. The particles were precipitated by addition of toluene, centrifuged, and redispersed in DMF. The washing step was repeated 3 times. The particles were then redispersed in acetonitrile, washed with toluene, and allowed to dry overnight under nitrogen. A stable colloidal solution of GaI_3 exchanged nanocrystals could be obtained in DMF plus 3 vol% HMPA in order to obtain absorption spectra.

Molten salt dispersal and subsequent gallium exchange. A $KGaI_4$ salt phase was obtained by grinding and mixing GaI_3 and KI in a 1:1 molar ratio and subsequently melting the mixture under stirring at 250°C on a hotplate (using a glass-coated stir-bar). A portion of 1.2 g of the salt mixture was combined with the GaI_3 ligand-exchanged NCs by adding them to the salt as a dry powder and combining them in a mortar and pestle. Next, the particle-salt mixture was loaded into a quartz ampoule and heated to 250°C under vigorous stirring to obtain a visually homogeneous dispersion. The ampoule was sealed under vacuum using an oxy-hydrogen torch, transferred into a muffle furnace, and annealed for different durations at different temperatures. A higher annealing temperature or longer duration led to higher extents of gallium incorporation.

QD recovery following molten salt treatment. The salt matrix was dissolved in acetonitrile (~6 mL) at 60 °C, and the QDs were recovered as a solid by centrifugation. The QD solid was washed with 4 mL fresh acetonitrile to remove residual salt. The particles could regain colloidal stability in nonpolar solvents through treatment with 1:1 molar dodecylamine/octadecylamine. The amine recovery was performed by utilizing an adaptation of the entropic ligand mixture concept introduced by the Peng group.[5] A solution of dodecylamine and octadecylamine (0.01 M each) was obtained by dissolving the corresponding ligands in toluene at 80^oC under stirring. 2 ml of this toluene solution was combined with the QD powder and stirred at 60^oC for an hour. The resulting QD solution in toluene was washed with methyl acetate to remove excess ligands and then stored in toluene.

ZnS shelling. In a typical shelling step using $In_{1-x}Ga_xP$ cores derived from InP particles of the large (Micic) and medium sizes (commercial), anhydrous $ZnCl_2$ (157 mg, 1.15 mmol) was combined with 5 ml of freeze-thaw purified oleylamine and 2.6 ml of TOP and degassed at 120^oC for 1 hour. The amine capped $In_{1-x}Ga_xP$ particles were introduced into the reaction mixture as a toluene solution (500 µL, ~30 mg/ml) under N_2 flow, followed by degassing at 120^oC for another 30 min. Next, the temperature was raised to 300^oC, and 2.4 ml of a 0.4 M solution of TOP-S was slowly injected into the reaction mixture at a rate of 1 ml/hour using a syringe pump. The reaction mixture was stirred at 300^oC for a total of 3 hours, cooled down, diluted with hexane, and washed with ethanol. Once separated from the reaction mixture, the particles were redispersed in hexane, washed using a mixture of methyl acetate and acetonitrile, and finally stored as a stable colloidal solution in hexane.

While shelling the 4 nm InP particles, native organic ligands were first exchanged with GaI_3, then the particles were dispersed in toluene with the addition of dodecylamine/ octadecylamine. Subsequently, the same synthetic protocol could be followed to grow a ZnS shell on the ligand exchanged particles.

In order to shell alloyed $In_{1-x}Ga_xP$ cores derived from small (Peng) InP particles, anhydrous $ZnCl_2$ (628 mg, 2.30 mmol) was combined with 7 ml of freeze-thaw purified oleylamine along with 3 ml of TOP and degassed at 120^oC for 1 hour. The amine-capped $In_{1-x}Ga_xP$ particles were introduced into the reaction mixture as a toluene solution (500 µL, ~30 mg/ml) under N_2 flow, followed by degassing at 120^oC for another 30 min. Next, the temperature was raised to 300^oC, and 4.8 ml of

a 0.8 M solution of TOP-S was slowly injected into the reaction mixture at a rate of 2 ml/hour using a syringe pump. The reaction mixture was stirred at 300°C for a total of 3 hours, cooled down, diluted with hexane, and washed with ethanol. Once separated from the reaction mixture, the particles were redispersed in hexane, washed using a mixture of methyl acetate and acetonitrile, and finally stored as a stable colloidal solution in hexane.

Core-shell InP/ZnSe/ZnS nanocrystals. Colloidal core-shell InP/ZnSe/ZnS nanocrystal samples with near unity PLQY were obtained from commercial sources as a solution in hexane and were stored in a nitrogen glove box.

Optical characterization. UV-vis spectra were collected on colloidal solutions of QDs with a Shimadzu UV-3600i Plus UV-Vis-NIR spectrophotometer in transmission mode. Photoluminescence emission spectra and Excitation-Emission Maps were collected on a Horiba Jobin Yvon Fluorolog-3 spectrophotometer equipped with a Synapse OE-CCD detector. Time resolved PL decay profiles were recorded with a Horiba Jobin Yvon Tempro fluorescence lifetime system with a 392nm pulsed LED source. High temperature PL spectra were recorded using a dilute colloidal solution of nanocrystals in ODE contained in a quartz ampoule sealed under inert atmosphere. A custom-built furnace setup interfaced with an Ocean Insight HR4Pro high-resolution spectrometer was utilized to record the high temperature PL spectra, which were corrected using a calibrated Ocean Insight HL-3plus precision lamp source. A 450 nm laser (CPS450, Thorlabs, 4.5 mW) was employed as the excitation source.

PL quantum yield was calculated relative to a dilute solution of the reference dye coumarin 153 in ethanol. Integrated areas of the recorded PL spectra were used to estimate the quantum yield as

$$\varphi = \varphi_{ref} \frac{I_s}{I_{ref}} \frac{f_{ref}}{f_s} \frac{\eta_s{}^2}{\eta_{ref}{}^2}$$

Where s is the sample and ref is coumarin 153. The quantum yield of coumarin 153 was taken as 0.53.[6] I represents the integrated PL intensity, f denotes the absorption factor at excitation wavelength, and n representing the refractive indices of the solvents. Absolute PLQY measurements were carried out using an integrating sphere (Labsphere) coupled with a computer-controlled spectrometer (Ocean Optics QE Pro) together with a 415-nm fiber-coupled LED excitation source (M415F3, Thorlabs, 14.4 mW).

Powder X-ray diffraction. Powder x-ray diffraction patterns were collected on a Rigaku Miniflex X-ray diffractometer. Samples were deposited on (511)-oriented Si low background substrates. For $In_{1-x}Ga_xP$ nanocrystals, the lattice parameter was extracted by employing Le Bail refinement to fit the full XRD pattern using the TOPAS software package (version 5, Bruker AXS). A twelfth-order Chebyshev polynomial was used to fit the background, and the crystalline phase was assumed to contain a zinc blende phase (F$\overline{4}$3m space group) exclusively. The lattice constants of bulk InP (5.8687 Å) and GaP (5.4505 Å) were used to calculate the alloy composition *x*, assuming a linear relationship between the lattice constant and alloying composition (Vegard's law) as follows:

$$a\left(In_{1-x}Ga_xP\right) = \left(1-x\right)a_{InP} + xa_{GaP}$$

Transmission electron microscopy. TEM images were obtained on an FEI Tecnai F30 microscope at 300 kV.

Small Angle X-ray Scattering (SAXS). Colloidal solutions of QDs in toluene were prepared in sealed Kapton capillaries for small-angle x-ray scattering (SAXS) experiments. SAXS patterns were collected using a SAXSLab Ganesha instrument with Cu Kα radiation (λ = 1.54 Å). The SAXS curves were analyzed by fitting to a quantitative model in Igor Pro using the Irena package (available at http://usaxs.xray.aps.anl.gov/staff/ilavsky/irena.html).[7] The scattering curves were fit in the particle size distribution module using the model-free Maximum Entropy approach. The particles' form factor was assumed to be that of a sphere with an aspect ratio of 1. The extracted size distributions were further fit with symmetric Gaussians to estimate average particle diameters along with the associated standard deviations.

X-Ray Fluorescence (XRF). XRF elemental analysis was performed using a Rigaku NEX DE instrument under helium atmosphere.

Transient Absorption Spectroscopy. Measurements were performed using a 35-fs, 2-kHz Ti:sapphire laser with time-delayed white light probe pulses produced in a 2 mm sapphire plate and 1-kHz pump pulses centered at 400 nm produced from second harmonic generation of the fundamental. At these pump energies, we are exciting the samples well above the band gap of the $In_{1-x}Ga_xP$ core, but below the absorption onset of the ZnS shell. All time-resolved absorption spectra were collected at room temperature from stirred solutions, beginning at low excitation fluence such that only single-exciton dynamics were observed.

Solid-State Nuclear Magnetic Resonance (SSNMR). All solid-state NMR experiments were performed on a Bruker wide-bore 9.4 T ($\nu_0(^1H)$ = 400 MHz) NMR spectrometer equipped with a Bruker Avance III HD console. All experiments were performed on the 2.5 mm HXY probe. The probe was configured in ^1H-^{31}P-^{13}C mode and the Y channel could be tuned from ^{115}In to ^{69}Ga. A MAS frequency of 25 kHz was used. ^1H, ^{31}P, ^{69}Ga, ^{115}In pulse durations and powers were directly optimized on the samples. ^1H chemical shifts were referenced to neat tetramethylsilane using adamantane ($\delta_{iso}(^1H)$ = 1.82 ppm) as a secondary chemical shift standard. ^{31}P, ^{69}Ga, and ^{115}In were directly referenced to the established chemical shift standards using the previously established relative NMR frequencies.[8]

^{1}H SSNMR. The ^1H spin echo[9] was performed with previously described pulse sequences and with 100 kHz RF fields ^1H pulses (2.5 μs π/2-pulse).

^{31}P SSNMR. The ^{31}P spin echo spectra were acquired with 90.1 kHz RF field ^{31}P pulses (2.75 μs π/2-pulse). SPINAL-64 heteronuclear decoupling was applied with an RF field of ca. 100 kHz.[10]

In all CP experiments, the spin lock pulses were linearly ramped from 90 to 100% of the spin lock RF fields.[11] $^1H \rightarrow {}^{31}P$ CP-HETCOR experiments used spin lock pulses with RF fields at 91 kHz RF and 116 kHz RF for ^1H and ^{31}P, respectively, and contact times were 1 ms. SPINAL-64 heteronuclear decoupling was applied with an RF field of ca. 100 kHz for ^{31}P NMR experiments.[10] eDUMBO1-22 homonuclear dipolar decoupling[12] was applied during the indirect ^1H dimension evolution to improve ^1H resolution in the $^1H \rightarrow {}^{31}P$ CP-HETCOR experiments. The initial phase and offset of eDUMBO1-22 was optimized directly on each sample and used 32 μs pulse durations and 100 kHz RF fields.

^{69}Ga SSNMR. The ^{69}Ga MAS NMR spectrum was acquired using the QCPMG pulse sequence.[13-15] The QCPMG spectrum was acquired with a spectral window of 2 MHz and an echo train comprising 24 echoes of 800 μs duration each, and ^{69}Ga π/2 and π pulse lengths of 2.25 and 4.50 μs, corresponding to a 55.6 kHz RF field and a 111.1 kHz CT nutation frequency. ^1H SPINAL-64 heteronuclear decoupling was applied with an RF field of ca. 100 kHz. The simulated ^{69}Ga spectrum was generated using ssNake[16] with a Czjzek distribution (σ) in the quadrupolar interaction parameters presented in the Supporting Information.

^{115}In SSNMR. The ^{115}In static NMR spectra were acquired using a spin echo pulse sequence,[9] and ^{115}In $\pi/2$ and π pulse lengths of 1.0 and 2.0 μs, corresponding to a 50 kHz RF field and a 250 kHz CT nutation frequency. The simulated ^{115}In spectrum was generated using ssNake[16] with a Czjzek distribution (σ) in the quadrupolar interaction parameters presented in the Supporting Information.
^{1}H{^{69}Ga}/^{1}H{^{115}In} S-RESPDOR Experiments. In the S-RESPDOR/S-REDOR experiments,[17-19] $SR4_1^2$ dipolar recoupling[20-22] was used. ^{1}H spectra with varying recoupling times were acquired with (S) and without (S_0) the application of a dephasing pulse on the heteronucleus. The duration of dipolar recoupling was incremented in a linear manner. For ^{69}Ga and/or ^{115}In S-RESPDOR experiments, a saturation pulse with a duration of 1.5 times rotor cycles and a ~ 100 kHz RF field was centered on the ^{1}H refocusing π-pulse. The ^{69}Ga and ^{115}In RF fields were experimentally optimized to give maximum saturation on each sample. The recoupling curves were obtained by plotting $\Delta S/S_0$ as a function of the recoupling time and were fitted using SIMPSON simulation v 4.1.1.[23]
^{1}H → ^{31}P{^{69}Ga, ^{115}In} *J*-resolved Experiments. ^{1}H → ^{31}P{^{69}Ga, ^{115}In} *J*-resolved experiments[24-27] were recorded with ^{1}H → ^{31}P CP at the beginning of the experiment. The ^{31}P{^{69}Ga, ^{115}In} *J*-resolved experiments were performed with ^{31}P π pulse and ^{69}Ga/^{115}In 1.5 times rotor cycles saturation pulses. The maximum *J*-evolution time was 8 ms. The *J*-resolved curves were obtained by plotting $\Delta S/S_0$ as a function of the total *J*-coupling time.

THEORETICAL MODELING OF $In_{1-x}Ga_xP$ NANOCRYSTALS.

Construction of $In_{1-x}Ga_xP$ QD geometries. To construct the alloyed QD geometries, an initial tetrahedral InP nanocluster was cut from the bulk zincblende InP configuration with In atoms as the surface terminations. The edge lengths of these tetrahedrons were chosen to match the sizes of the experimentally synthesized QDs. Since the $In_{1-x}Ga_xP$ QDs are experimentally synthesized from spherical InP QDs, we converted the diameter (d) of the spherical InP QD to the edge length (a) of the tetrahedral $In_{1-x}Ga_xP$ QDs by equating their volumes. Thus, the tetrahedral $In_{1-x}Ga_xP$ QDs constructed from 4.0nm diameter InP QD have edge lengths of around 6.6nm.

Next, In cations were randomly selected from the initial configuration and replaced with Ga cations. The number of In-to-Ga exchanges was calculated by the desired Ga percentage (14%, 38%, 57%, 65%, 78%, 92%, and 100%). For each Ga composition, three different random-In-to-

Ga exchanges were considered. $In_{1-x}Ga_xP$ QDs with inhomogeneous gallium distribution were also considered. See the end of this subsection for detailed descriptions.

To account for the local strain effects due to the cation exchange, the structures were then relaxed using the conjugate gradient descent algorithm implemented in LAMMPS. The Tersoff-type classical force field previously parameterized for the III-V semiconductors of interest was used for the energy minimization.[28] Lastly, the singly-bonded P atoms at each of the four corners of the tetrahedron were removed, and the QD was passivated with ligand potentials.

The relevant $In_{1-x}Ga_xP$ structures are tabulated below in Table S1. The geometry of an $In_{0.35}Ga_{0.65}P$ constructed from 4.0nm InP QD with random gallium distribution is shown in Fig. S1.

Table S1. The relevant tetrahedral $In_{1-x}Ga_xP$ QD structures constructed from 4.0nm InP spherical QD used in the calculations of this work. The numbers of In, Ga, and P atoms for each gallium content percentage are tabulated.

	In atoms	Ga atoms	P atoms
Spherical InP	727	0	736
$In_{0.86}Ga_{0.14}P$	829	136	816
$In_{0.62}Ga_{0.38}P$	597	368	816
$In_{0.43}Ga_{0.57}P$	417	548	816
$In_{0.35}Ga_{0.65}P$	339	626	816
$In_{0.22}Ga_{0.78}P$	211	754	816
$In_{0.08}Ga_{0.92}P$	78	887	816
GaP	0	965	816

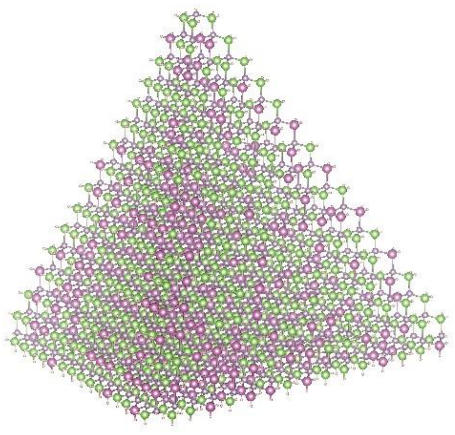

Figure S1. The configuration of an $In_{0.35}Ga_{0.65}P$ QD used in the electronic structure calculations. The alloyed QD is constructed from 4.0nm InP with random In-to-Ga cation exchanges. The pseudo-ligands used for surface passivation in the atomistic pseudopotential calculations are replaced with H atoms for clarity. Colors: Pink-In, Green-Ga, Purple-P, White-H.

In addition to uniformly distributed Ga, alloyed QDs with a Ga-rich centers or Ga-rich exteriors were also constructed and calculated. In these cases, at the step of the QD construction where In cations were replaced with Ga, the cations were categorized into the "center" and the "exterior" by the sphere that inscribes tetrahedron. Random In-to-Ga replacements were then carried out in each section, with a higher Ga concentration within (or outside of) the inscribed sphere. The rest of the theoretical treatments remain the same.

Calculated optoelectronic properties of $In_{1-x}Ga_xP$ QDs with inhomogeneous Ga distributions (particularly the Ga-rich surface QDs) show qualitative deviations from experimental measurements in this work, further reaffirming the homogeneous alloying of cations during the cation exchange synthesis step. As shown in Figure 1 of main text, the calculated optical gap of $In_{1-x}Ga_xP$ QDs with Ga-rich surfaces is very different from those calculated for the homogeneous gallium distributions as well as the experimental observations. Further, as shown below in Fig. S2, the oscillator strength and absorption spectra are also qualitatively different for QDs with Ga-rich surfaces, manifesting in a different first peak energy and more pronounced higher energy peaks. These theoretical results of optoelectronic properties corroborate the experimental evidence from ssNMR that homogeneous gallium alloying occurs in the $In_{1-x}Ga_xP$ QDs in this work.

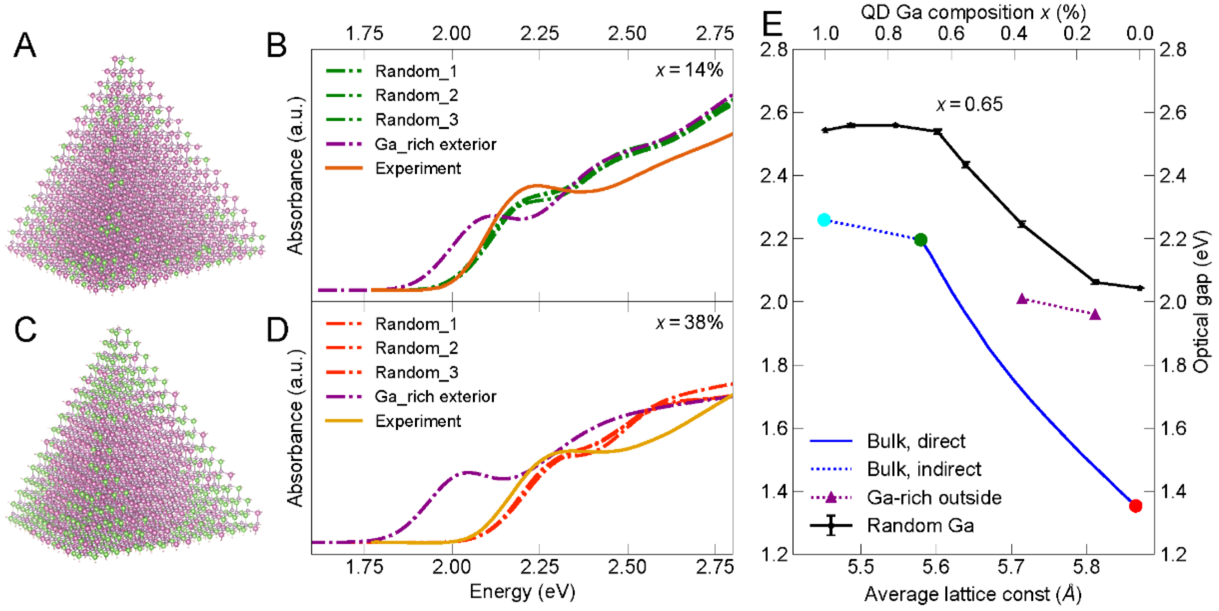

Figure S2. (A,C) The geometries of the $In_{1-x}Ga_xP$ QDs with Ga-rich exterior for x = 14% and x = 38% are shown. **(B,D)** The calculated absorption spectra (dash-dot lines) for $In_{1-x}Ga_xP$ QDs with x = 14% and 38% gallium composition are compared to the experimental measurements (solid lines). For the theoretical calculations, three QDs with spatially random gallium distributions and one QD with Ga-rich exterior are calculated and shown. **(E)** The optical gaps of $In_{1-x}Ga_xP$ QDs with spatially random gallium distributions and with Ga-rich exterior are shown and compared with the band gaps of bulk alloys.

Fitting atomistic pseudopotentials. The excitonic states and energies of the alloyed $In_{1-x}Ga_xP$ nanocrystals were calculated using the atomistic semiempirical pseudopotential method[29-32] combined with the Bethe-Salpeter Equation (BSE).[33-34] The pseudopotentials of In, Ga, P and As were fitted to accurately reproduce the literature bulk band structures[35-36] and bulk deformation potentials[37] of four binary semiconductors: InP, GaP, InAs and GaAs. Following previous work,[31, 38] we used pseudopotentials with a local form in the reciprocal space with six fitting parameters $\{a_0, a_1, a_2, a_3, a_4, a_5\}$:

$$\tilde{v}_\mu(q) = a_0\left(1 + a_4 Tr\epsilon_\mu + a_5\left(Tr\epsilon_\mu\right)^3\right)\frac{q^2 - a_1}{a_2\exp\left(a_3 q^2\right) - 1} \quad \text{(Eq. S1)}$$

where $\tilde{v}_\mu(q)$ is the pseudopotential around atom μ (In, Ga, P, or As) in the reciprocal space. ϵ_μ is the local strain tensor around atom μ, and its trace is calculated using the tetrahedron volume formed by the nearest neighbors.[38] Parameters a_0 through a_3 were fitted to the bulk band structures of InAs, InP, GaAs, GaP obtained through high-accuracy electronic structure calculations by Cohen et al[35-36] The local strain-dependent prefactors (a_4 and a_5) were fitted to reproduce the literature bulk deformation potentials of the CBM and VBM for each semiconductor material.[37]

The parameters for fitted pseudopotentials of As, Ga, In, and P are summarized in Table. S2. The a_5 parameter for the cubic strain tensor term is only needed for Ga to give well fitted results to the bulk deformation potentials of Ga-containing binary semiconductors. Fig. S3 shows the bulk band structures and deformation potentials generated from our pseudopotentials as compared to literature, in good quantitative agreement.

Table S2. The a_0 through a_5 parameters for the best-fit atomistic pseudopotentials of As, Ga, In, P. See Eq. S1 for the function form of the pseudopotential.

	a_0	a_1	a_2	a_3	a_4	a_5
As	25.68513132	2.69329915	1.63958593	0.49218946	0.01647533	0
Ga	48.55247982	2.28309682	3.77112430	0.45685179	0.56676447	8.09704232
In	50.52631742	1.98680525	3.15524320	0.43784813	0.36378779	0
P	28.76635710	2.67994632	1.78491672	0.46023571	-0.00357477	0

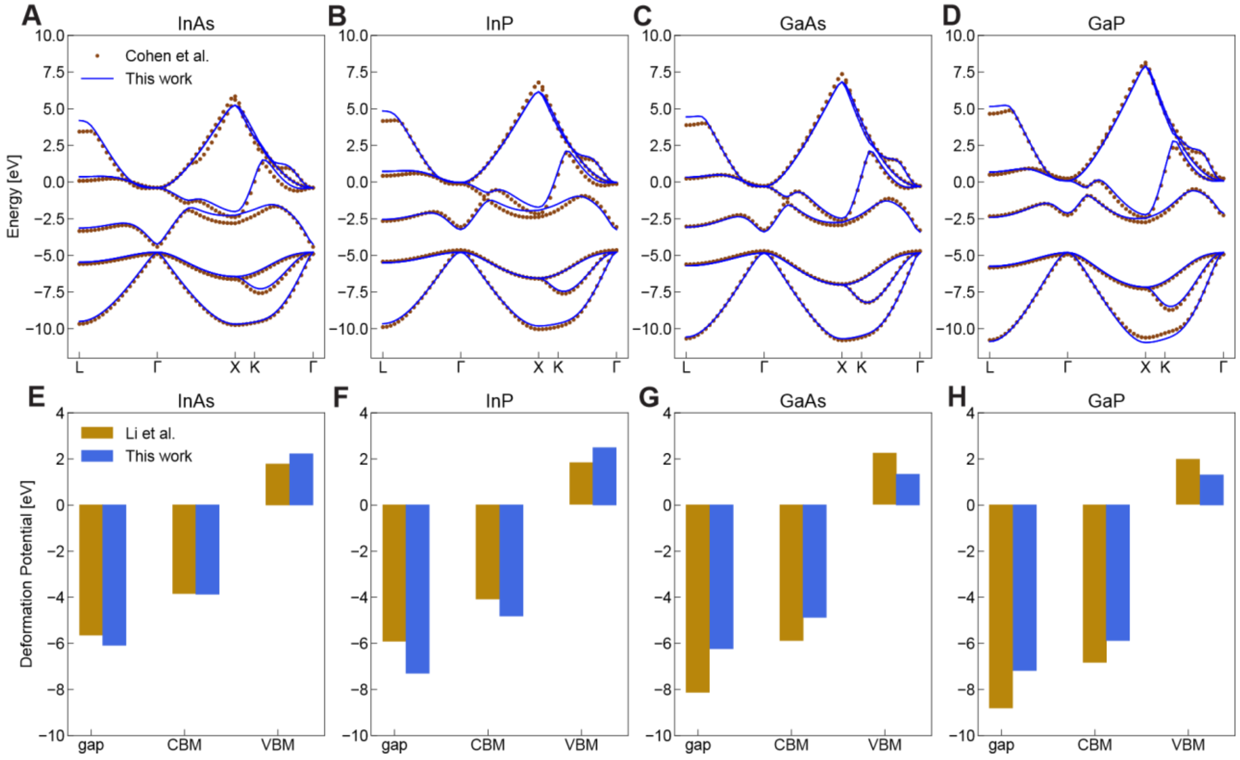

Figure S3. The bulk band structures (A-D) and deformation potentials (E-H) calculated using the fitted pseudopotentials of this work (blue lines and bars) is compared to the literature bulk properties (gold dots and bars)[35-37] for the relevant binary semiconductors: InAs, InP, GaAs, and GaP.

Calculation of excitonic states, absorption spectra, and radiative lifetimes. The fitted pseudopotentials were Fourier transformed into the real-space form and used to construct the single quasiparticle Hamiltonian in the non-interacting electron picture:

$$H_{qp} = -\frac{1}{2}\nabla^2 + \sum_{\mu} \hat{v}_{\mu}\left(\left|\mathbf{r} - \mathbf{R}_{\mu}\right|\right) \qquad \text{(Eq. S2)}$$

where $\hat{v}_{\mu}(\boldsymbol{r})$ is the pseudopotential around atom μ in the real space. The stochastic filter diagonalization technique was then used to obtain the electron $\phi(\boldsymbol{r}_e)$ and hole states $\phi(\boldsymbol{r}_h)$ of the QD near its band edge. The real-space grid spacing of 0.8 Bohr was sufficient to converge the solutions. The quasiparticle eigenstates were then used as linear combinations of electron-hole product wavefunctions and the coefficients $c_{a,i}^{n}$ (Eq. S3) were obtained by solving the Bethe-

Salpether equation within the static screening approximation[6] and the Tamm-Dancof approximation [39]

$$\psi_n(\mathbf{r}_e,\mathbf{r}_h)=\sum_{a,i} c_{a,i}^n \phi_a(\mathbf{r}_e)\phi_i(\mathbf{r}_h) \quad \text{(Eq. S3)}$$

where *a* and *i* are electron and hole state indices, respectively. All the BSE calculations were converged to 0.001eV with the linear combination of 60 electron states and 100-200 holes states, depending on the size of the alloyed QD. The dielectric constants for the alloyed $In_{1-x}Ga_xP$ QDs were chosen to be the same as the InP QDs of the same size.[40-42] Given the similar bulk static dielectric constants for InP and GaP, gallium composition is assumed to have a negligible effect on the dielectric constants of the alloyed QDs. For alloyed QDs synthesized from 4.0nm InP, the dielectric constant of $\epsilon = 11.45$ was used.

The oscillator strength for each exciton transition was calculated following the equation given in the main text, where the dipole moment $\boldsymbol{\mu}_n$ for exciton *n* was calculated using the quasiparticle wavefunctions and the BSE coefficients (Eq. S4)

$$\boldsymbol{\mu}_n = e\sum_{a,i} c_{a,i}^n \langle \phi_a | \mathbf{r} | \phi_i \rangle \quad \text{(Eq. S4)}$$

The absorption spectrum was calculated using the exciton energies and oscillator strength and broadened with Gaussian functions to reflect the inhomogeneity arising from size dispersion, as shown in Eq. S5.

$$I(\hbar\omega)=\sum_n \frac{1}{\sqrt{2\pi}\Gamma_n} f_n \exp\left[-\frac{(\hbar\omega-E_n)^2}{2\Gamma_n^2}\right] \quad \text{(Eq. S5)}$$

where E_n and f_n are the energy and oscillator strength for exciton *n*. E_g is the bulk material gap, which is interpolated between $E_g^{InP} = 1.34$ eV and $E_g^{GaP} = 2.27$ eV for alloyed QDs. $\Gamma_n \propto E_n - E_g$ is the energy-dependent inhomogeneous broadening.[43]

The radiative lifetimes were calculated using the equations given in the main text. As stated in the main text, the total rate given in Eq. S6 is given by a canonical average over the Boltzmann distribution of excitonic states:

$$\tau_r^{tot} = \frac{1}{k_r^{tot}} = \frac{1}{\frac{1}{Z}\sum_n \frac{e^{-\beta(E_n-E_0)}}{\tau_r^n}} \quad \text{(Eq. S6)}$$

where $Z = \sum_n e^{-\beta(E_n - E_0)}$ is the partition function. The results for the alloyed $In_{1-x}Ga_xP$ QDs are tabulated, with multiple entries corresponding to different random Ga-distributions during the construction of the QDs.

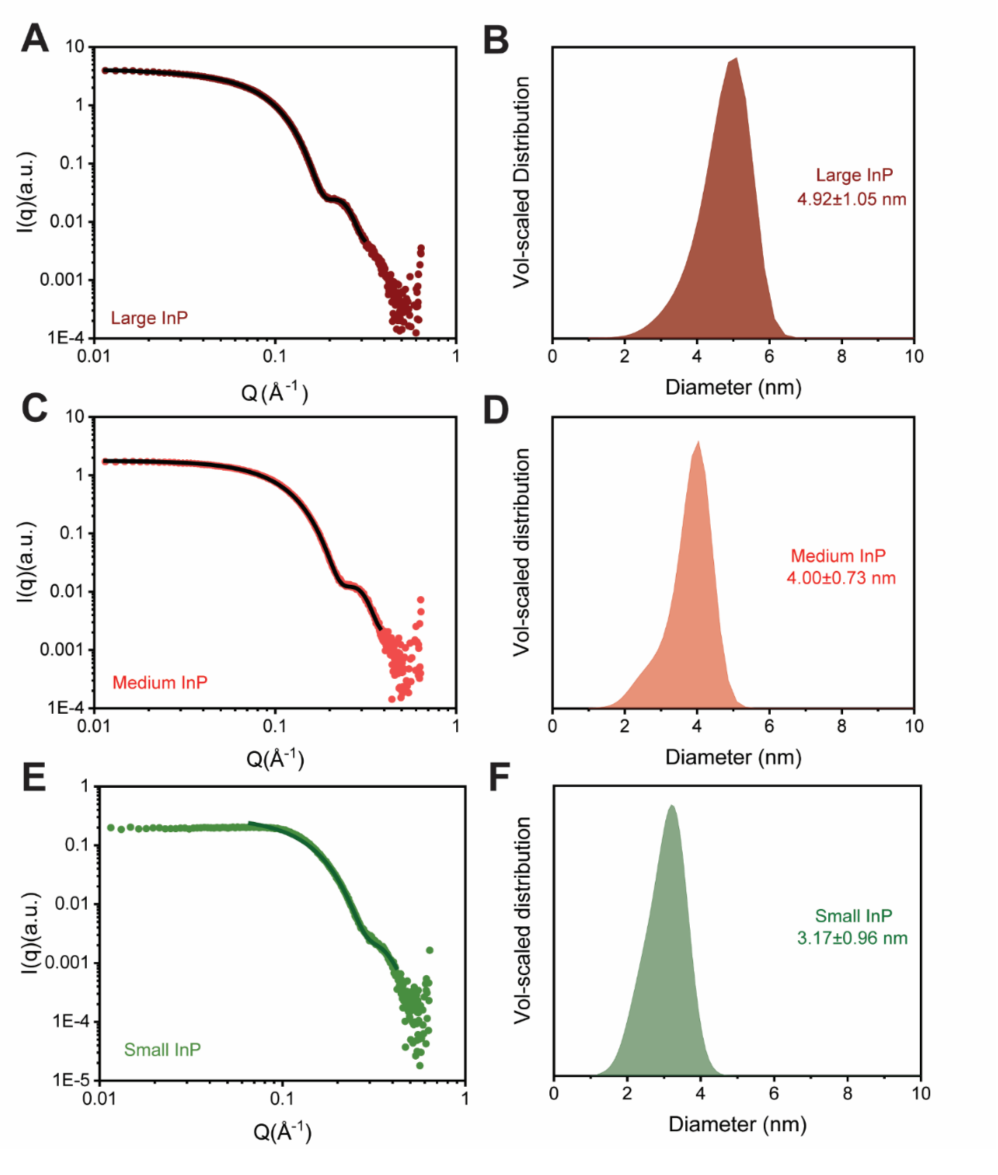

Figure S4. Size analysis: small-angle X-ray scattering (SAXS) data and maximum-entropy fits for **(A)** size-selected large InP QDs, **(C)** medium-sized InP QDs and **(E)** small InP QDs. **(B)**, **(D)** and **(F)** respectively are volume-scaled size distributions of the same obtained from a maximum-entropy fit to the SAXS data.

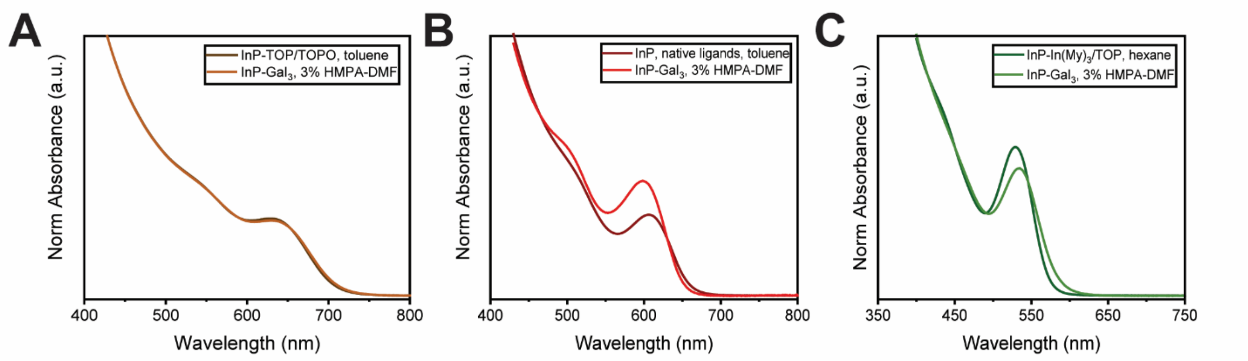

Figure S5. Comparison of optical absorption spectra of colloidal solutions of the relevant InP populations before and after GaI_3 ligand exchange: **(A)** 4.9 nm InP, **(B)** 4 nm InP and **(C)** 3.2 nm InP.

Table S3. Correlation of the molten $KGaI_4$ salt cation exchange conditions with resultant gallium compositions and optical characterization plots of the corresponding core-shell samples.

Average diameter of the initial InP QDs	Annealing temperature (°C)	Annealing duration (hr)	Estimated Ga content from PXRD x : $In_{1-x}Ga_xP$	Lattice Mismatch with ZnS	Corresponding panel in Fig. S6	
					Excitation-emission matrix	Time-resolved PL decay
4.92 nm	440	1	0.38	5.5%	A	B
4.00 nm	420	1	0.35	5.8%	C	D
3.17 nm	320/350	1+1	0.34	5.8%	E	F

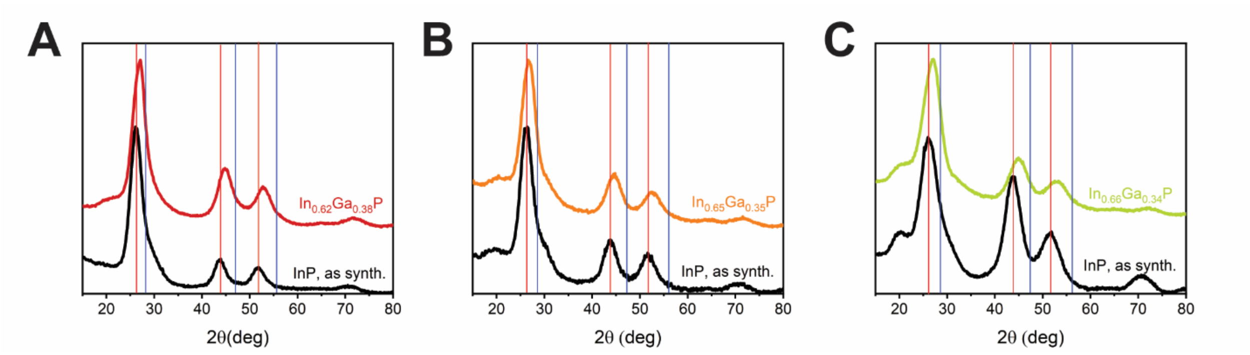

Figure S6. Diffraction patterns utilized to estimate the gallium composition in alloyed $In_{1-x}Ga_xP$ samples synthesized in a molten $KGaI_4$ salt matrix starting from **(A)** large 4.92 nm InP **(B)** medium 4.00 nm InP and **(C)** small 3.17 nm InP. Additional peaks appearing before (*i.e.*, at lower 2θ) the (111) peak in **(B)** and **(C)**, likely stem from the ordering of organic capping ligands.[44]

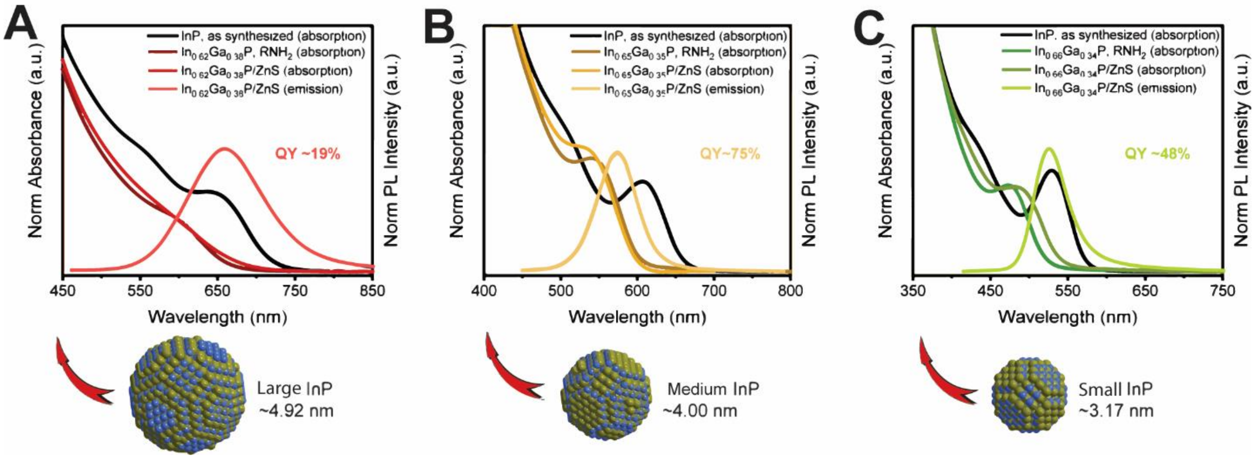

Figure S7. Absorption and emission spectra of emissive core-shell $In_{1-x}Ga_xP/ZnS$ samples with similar gallium contents, along with absorption spectra of the relevant InP and $In_{1-x}Ga_xP$ cores, synthesized from **(A)** 4.9 nm large InP (red emitting post cation exchange), **(B)** 4.0 nm medium InP (yellow) and **(C)** 3.2 nm small InP (green) populations.

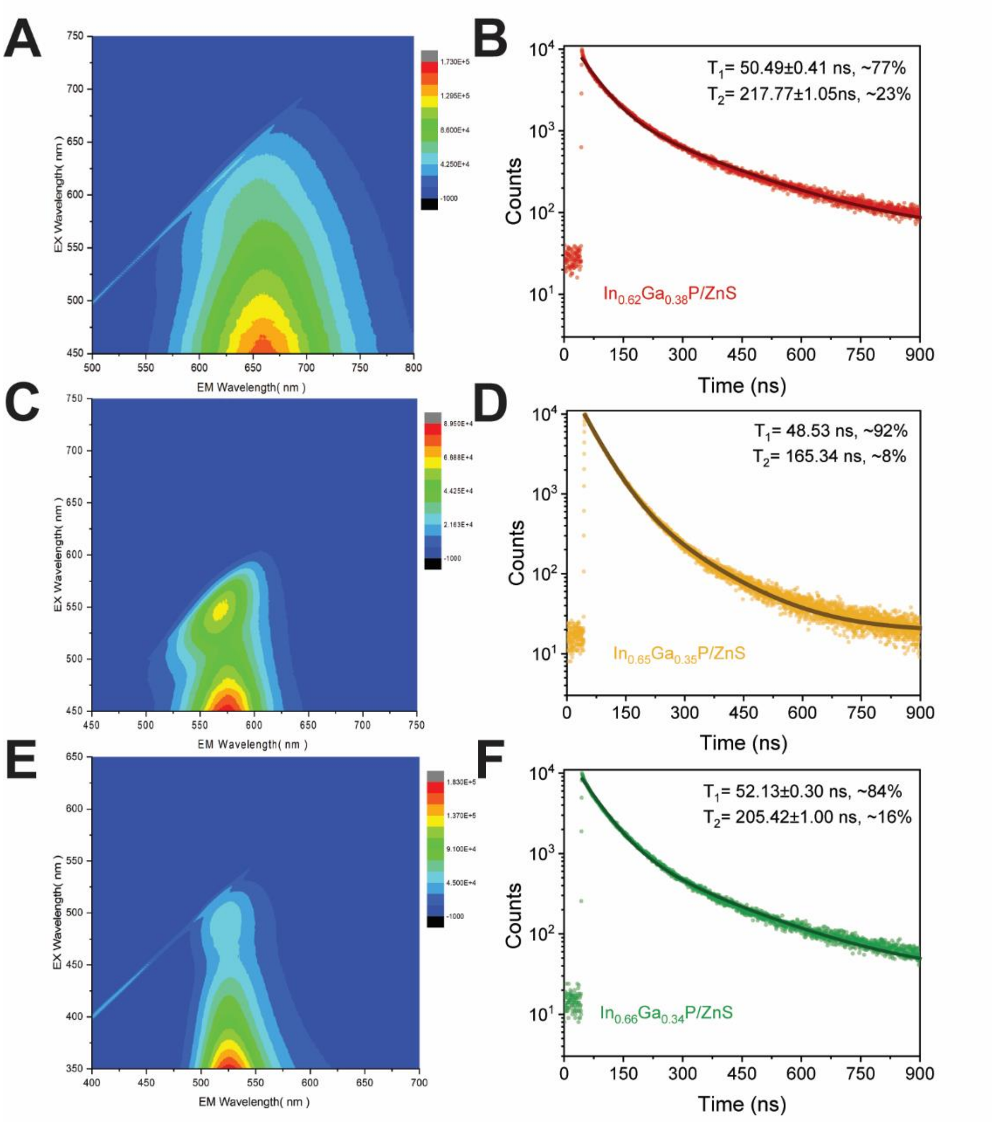

Figure S8. Excitation-emission matrices were constructed to optically characterize the bright $In_{1-x}Ga_xP/ZnS$ core-shell samples synthesized from initial InP nanocrystals with different surfaces and sizes: **(A)** $In_{0.62}Ga_{0.38}P/ZnS$ from large 4.92 nm InP QDs, **(C)** $In_{0.65}Ga_{0.35}P/ZnS$ from medium 4.00 nm InP QDs and **(E)** $In_{0.66}Ga_{0.34}P/ZnS$ from small 3.17 nm InP QDs. Recording time-resolved PL and fitting the profile to a biexponential decay allow us to estimate the lifetime of excitonic decay in the same samples **(B)**, **(D)** and **(F)**, respectively. The molten salt annealing conditions and resultant gallium contents are matched with the optical characterization panels in the following table S2.

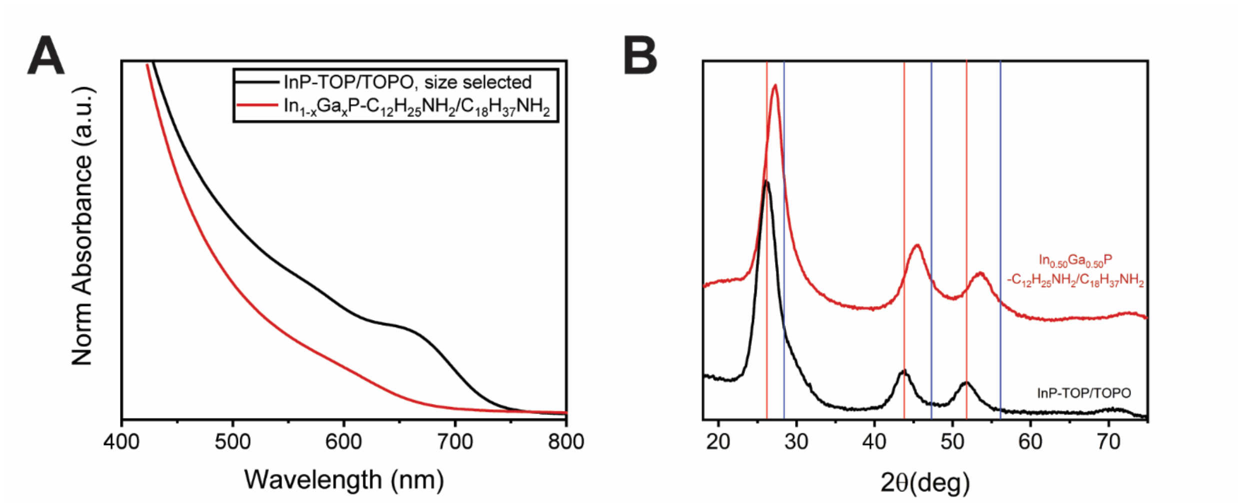

Figure S9. Characterization of the alloyed $In_{1-x}Ga_xP$ nanocrystalline sample used for solid state NMR studies: **(A)** Optical absorption spectrum exhibits a blue shift of the excitonic feature indicative of an increased bandgap. **(B)** Diffraction patterns indicate a pure zinc blende phase with peaks shifted to higher 2θ values. The alloyed sample was derived by cation exchange from the large Micic InP QDs, and a gallium composition of 50% was estimated from PXRD.

Table S4. Summary of Czjzek distributions (σ) in the quadrupolar interaction parameters derived from simulations of the spectra.

	δ_{iso} (ppm)	σ (MHz)	$\overline{C_Q}$ (MHz)	$C_{Q,\ peak}$ (MHz)	$\overline{P_Q}$ (MHz)	$P_{Q,\ peak}$ (MHz)	Lorentz (Hz)	Gauss (ppm)
^{115}In of InP QDs	750.4	12.58	24.953	22.449	27.423	25.559	0	38.25
^{115}In of $In_{1-x}Ga_xP$ QDs	714.3	15.03	29.813	26.531	32.764	30.206	0	276.4
^{69}Ga of $In_{1-x}Ga_xP$ QDs	311.5	2.878	5.709	5.306	6.274	6.041	82.96	32.35

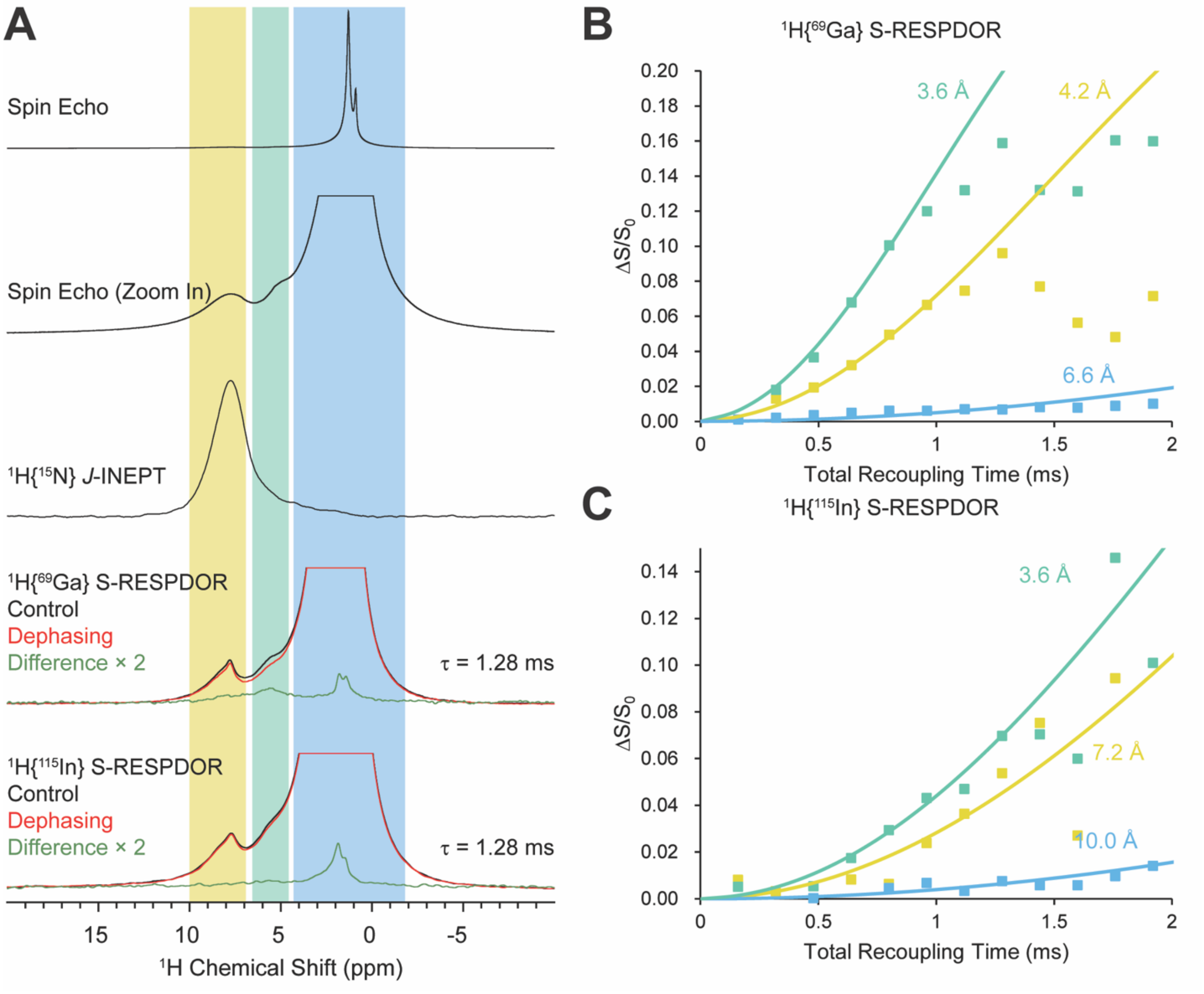

Figure S10. (A) ^{1}H spin echo spectra, ^{1}H{^{15}N} *J*-INEPT spectra, ^{1}H{^{69}Ga} S-RESPDOR control, dephasing, and difference spectra, ^{1}H{^{115}In} S-RESPDOR control, dephasing, and difference spectra of $In_{1-x}Ga_xP$ QDs. **(B & C)** The experimental $\Delta S/S_0$ intensities as a function of total recoupling time. Yellow squares, green squares, and blue squares correspond to the respective ranges of ^{1}H chemical shifts in **(A)**. Yellow, green, and blue lines are simulated dephasing curves for the ^{1}H–^{69}Ga and ^{1}H–^{115}In spin systems. The best-fit distances are indicated on the plots.

Table S5. Correlation of the molten $KGaI_4$ salt cation exchange conditions with resultant gallium compositions and optical characterization plots of the corresponding core-shell samples.

Annealing temperature (°C)	Annealing duration (hr)	Estimated Ga content from PXRD x : $In_{1-x}Ga_xP$	Estimated Ga content from XRF / Elemental Analysis x : $In_{1-x}Ga_xP$	Corresponding panel in Fig. S11		
				Relevant absorption and emission spectra	Excitation -emission map	Time-resolved PL decay
362	1	0.14	0.39	A	B	C
380	1	0.20	0.45	D	E	F
400	1	0.27	0.51	G	H	I
420	1	0.35	0.60	J	K	L
460	1	0.47	0.69	M	N	O
440	24	0.57	0.78	P	Q	R

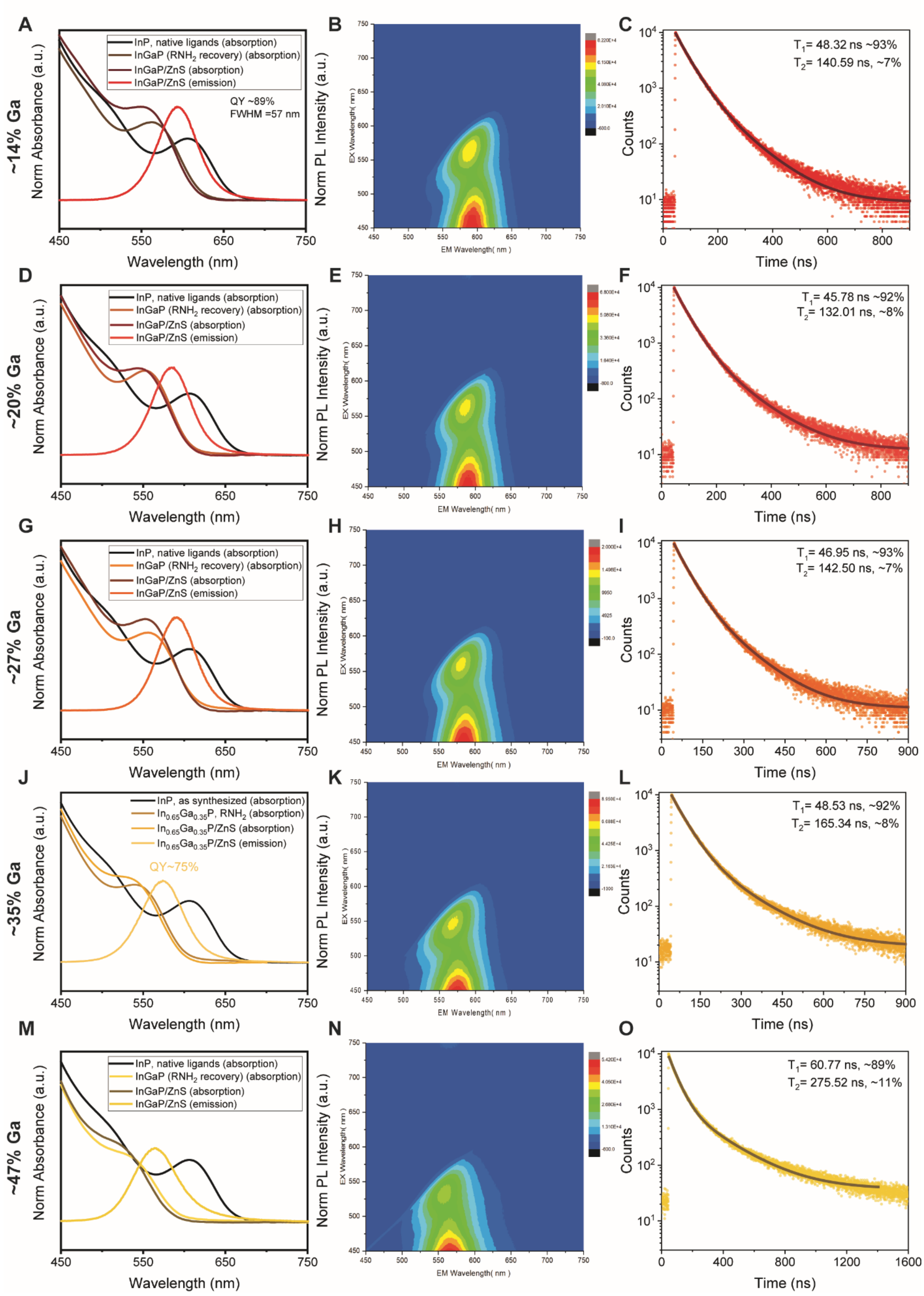
A
~14% Ga
Norm Absorbance (a.u.)
InP, native ligands (absorption)
InGaP (RNH2 recovery) (absorption)
InGaP/ZnS (absorption)
InGaP/ZnS (emission)
QY ~89%
FWHM =57 nm
Wavelength (nm)
B
Norm PL Intensity (a.u.)
EX Wavelength(nm)
EM Wavelength(nm)
C
T1= 48.32 ns ~93%
T2= 140.59 ns, ~7%
Counts
Time (ns)
D
~20% Ga
E
F
T1= 45.78 ns ~92%
T2= 132.01 ns, ~8%
G
~27% Ga
H
I
T1= 46.95 ns, ~93%
T2= 142.50 ns, ~7%
J
~35% Ga
InP, as synthesized (absorption)
In0.65Ga0.35P, RNH2 (absorption)
In0.65Ga0.35P/ZnS (absorption)
In0.65Ga0.35P/ZnS (emission)
QY~75%
K
L
T1= 48.53 ns, ~92%
T2= 165.34 ns, ~8%
M
~47% Ga
N
O
T1= 60.77 ns, ~89%
T2= 275.52 ns, ~11%

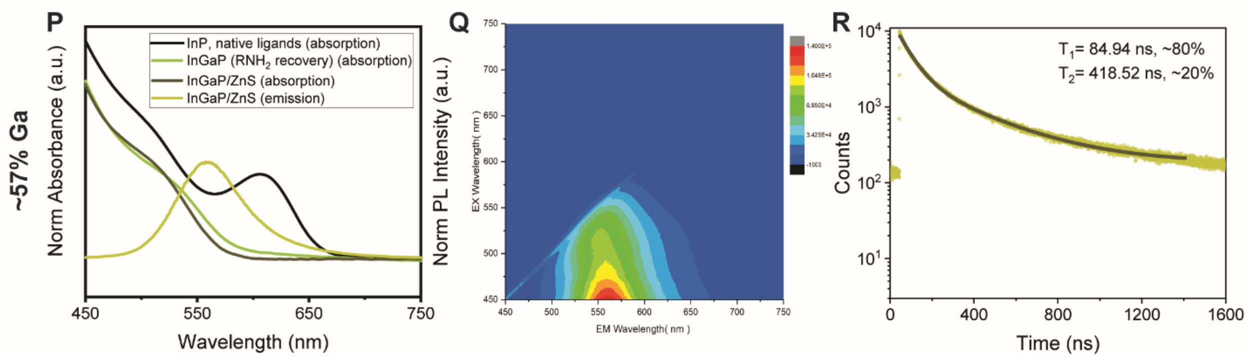

Figure S11. Optical characterization of individual core-shell $In_{1-x}Ga_xP/ZnS$ samples with varying gallium content, synthesized from the 4.0 nm InP QDs. Absorption and emission spectra of the relevant nanocrystalline core and core-shell samples, along with the corresponding excitation-emission matrices and time-resolved PL decay profiles are provided. The molten salt annealing conditions and resultant gallium contents are matched to the optical characterization panels in Table S5.

Table S6. Correlation of the molten $KGaI_4$ salt cation exchange conditions performed on large Micic InP with the resultant gallium compositions.

Annealing temperature (°C)	Annealing duration (hr)	Estimated Ga content from PXRD<br>x-value : $In_{1-x}Ga_xP$	PLQY
440	1	0.38	~19%
450	24	0.65	~2%
450	30	0.78	Non-emissive
450	48	0.92	Non-emissive

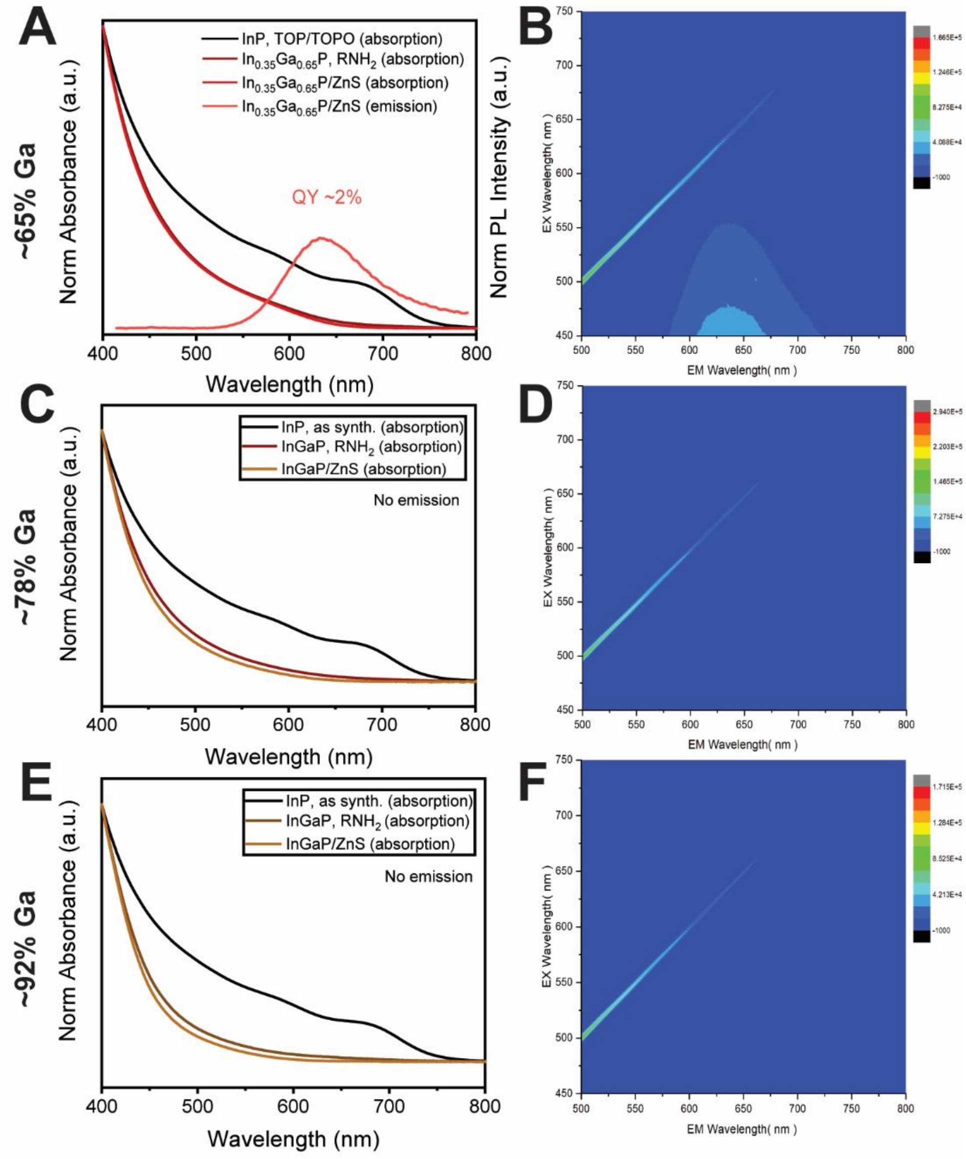

Figure S12. Optical characterization of the core-shell $In_{1-x}Ga_xP/ZnS$ samples with high Ga content derived from the large 4.92 nm InP QDs. Absorption and emission spectra of the core-shell $In_{0.35}Ga_{0.65}P/ZnS$ sample are provided in **(A)**. The emission is weak, with a solution phase PLQY of ~2%; **(B)** the corresponding excitation-emission matrix. Samples with higher gallium content are completely non-emissive, as indicated in the associated excitation-emission matrices. **(C)** and **(D)** pertain to the core-shell $In_{0.22}Ga_{0.78}P/ZnS$ sample; **(E)** and **(F)** pertain to the $In_{0.08}Ga_{0.92}P/ZnS$ sample.

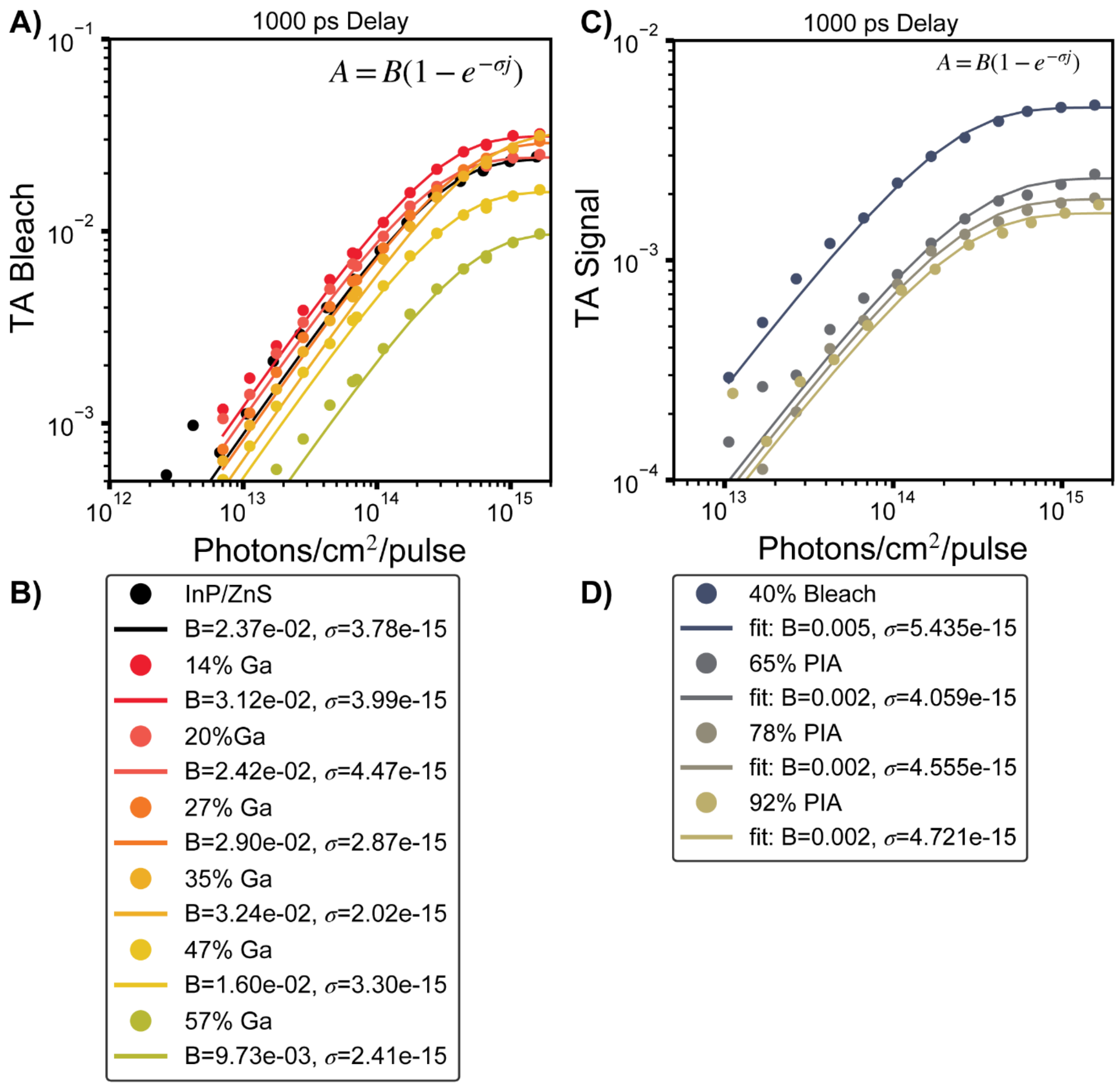

Figure S13. Determination of the absorption cross section at 400 nm for $In_{1-x}Ga_xP/ZnS$ nanocrystals. **(A)** Fluence dependent TA bleach signal for 4 nm core $In_{1-x}Ga_xP/ZnS$ nanocrystals with fits to Poissonian single exciton statistics. From this we extract the bleach amplitude scaling factor and the absorption cross section at 400 nm. **(B)** These cross sections are used to determine the absolute absorbance at the band edge for the $In_{1-x}Ga_xP/ZnS$ nanocrystals shown in Figure 6E. **(C)** Fluence dependent TA signal for 4.9 nm core $In_{1-x}Ga_xP/ZnS$ (bleach for 40% Ga, PIA for 65-92% Ga) with fits to Poissonian single exciton statistics. From this we extract the bleach amplitude scaling factor and the absorption cross section at 400 nm. **(D)** These cross sections are used to determine the absolute absorbance at the band edge for the $In_{1-x}Ga_xP/ZnS$ nanocrystals shown in Figure 6D. We note that 400nm is below the absorption onset for the ZnS shell, so we are primarily measuring the absorption cross section of the III-V core.

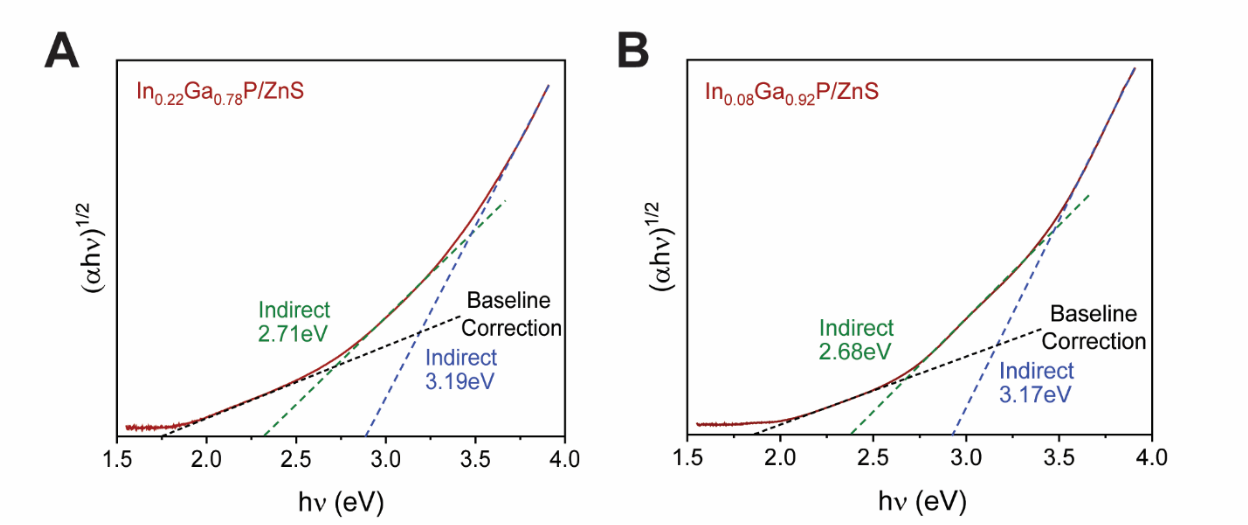

Figure S14. Tauc plots pertaining to an indirect band alignment were constructed from the absorbance values of the optically dark **(A)** $In_{0.22}Ga_{0.78}P/ZnS$ and **(B)** $In_{0.08}Ga_{0.92}P/ZnS$ samples.

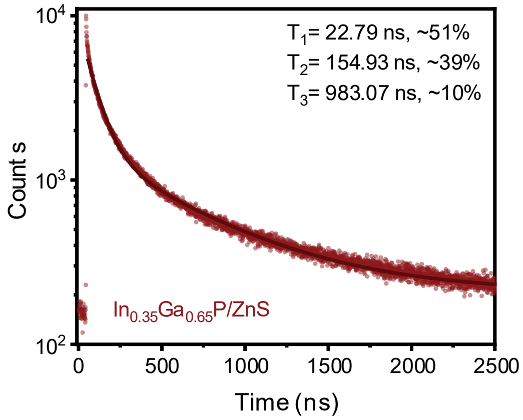

Figure S15. Time-resolved PL dynamics for 4.9 nm core diameter $In_{0.35}Ga_{0.65}P/ZnS$ nanocrystals.

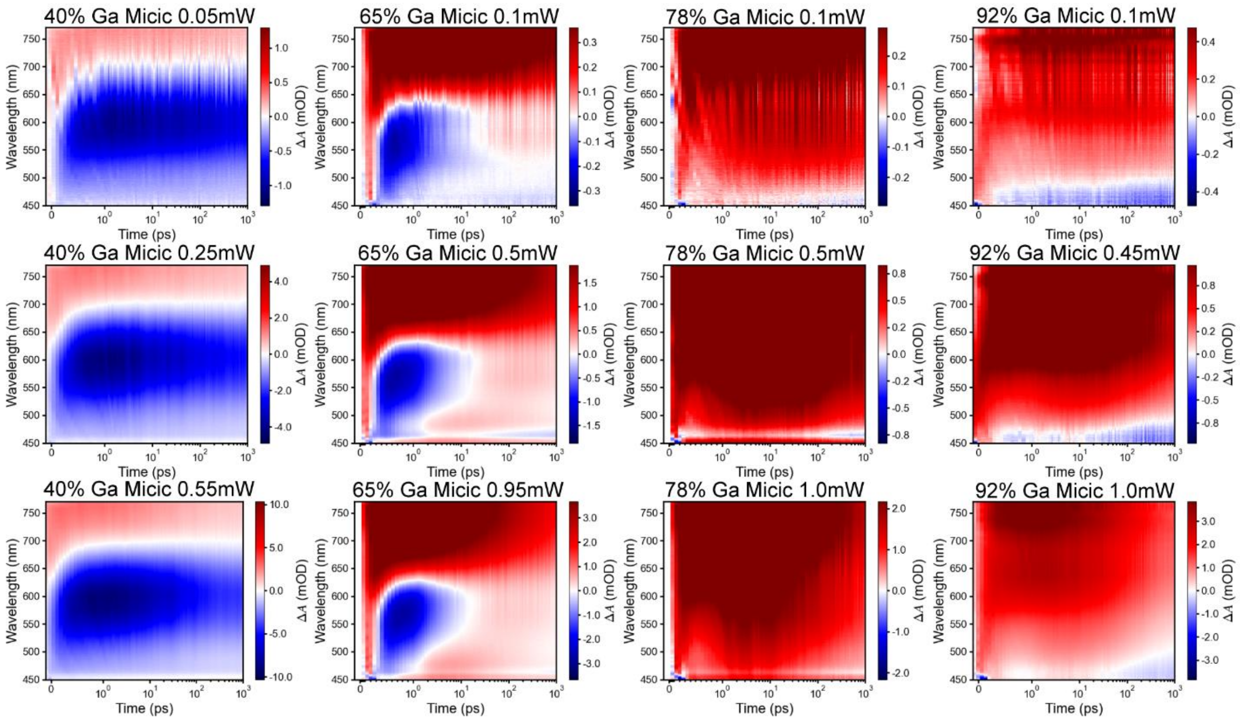

Figure S16. Transient absorption dynamics for 4.9 nm core diameter $In_{1-x}Ga_xP/ZnS$ nanocrystals as a function of increasing Ga content from left to right (40% Ga left to 92% Ga right) and a function of increasing excitation power from top to bottom. The top plots correspond to dynamics for single excitons, the middle plots correspond to dynamics for single and bi-excitons, and the bottom plots contain multi-exciton populations.

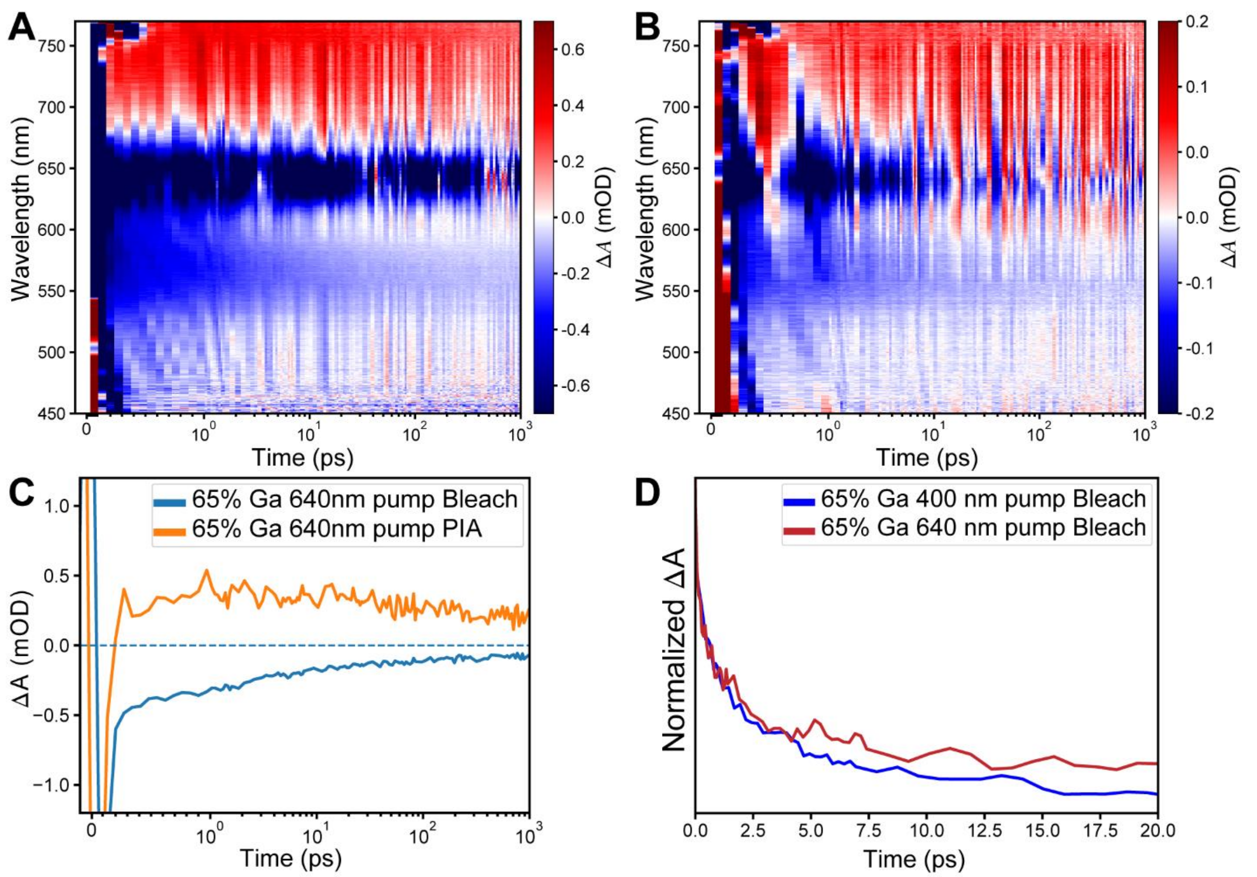

Figure S17. Transient absorption dynamics of 4.9 nm diameter core $In_{0.35}Ga_{0.65}P/ZnS$ pumped with 640nm light to directly access the ground state. **(A)** 1.0 mW pump and **(B)** 0.24 mW pump. **(C)** Dynamics of the bleach signal and PIA signal for a 1.0 mW pump and **(D)** comparison of the normalized bleach signal for a 400 nm pump and a 640 nm pump, indicating similar dynamics regardless of pump energy.

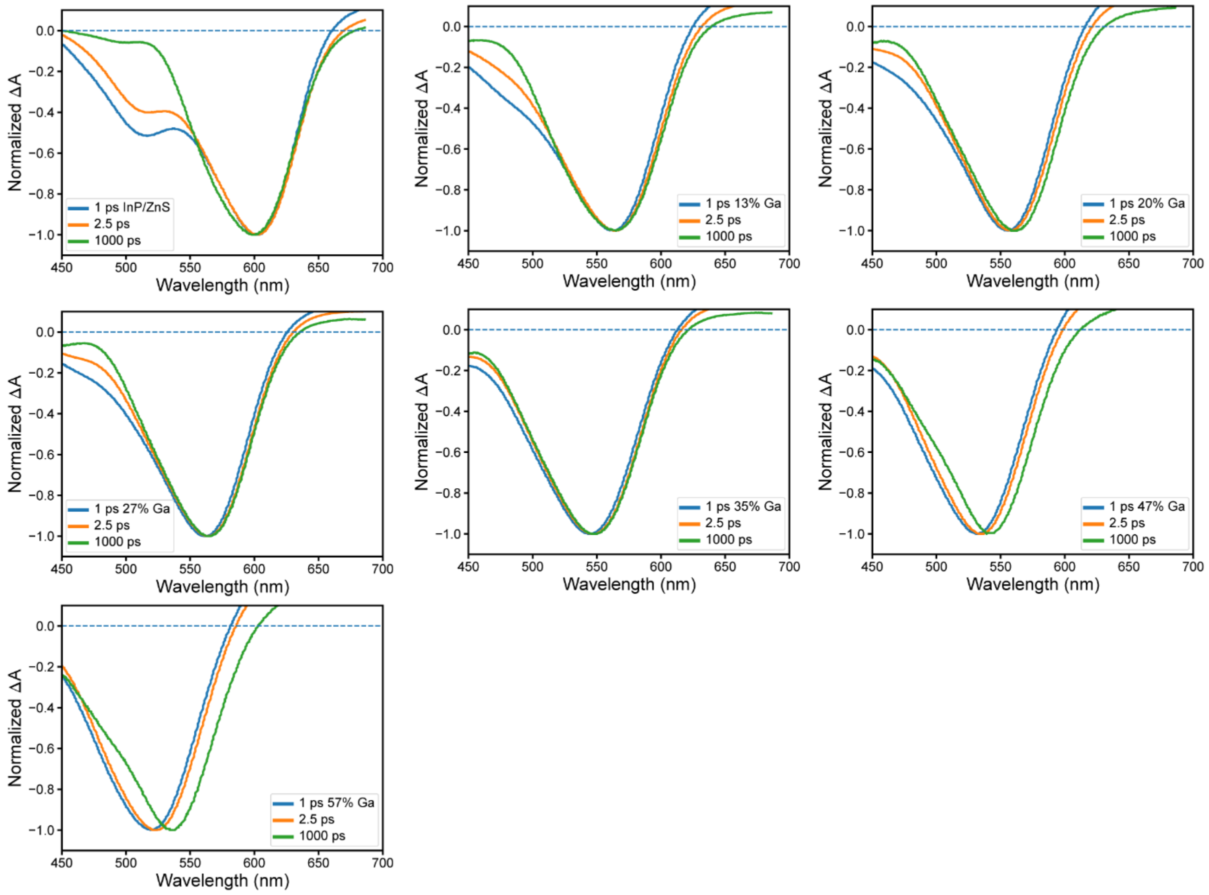

Figure S18. Transient absorption spectra for the 4 nm $In_{1-x}Ga_xP/ZnS$ under high power excitation $\left(\langle N \rangle \sim 10\right)$ for early times (1ps, blue and 2.5ps, orange) before Auger recombination and after Auger recombination (1000ps, green). For the samples from x=0-0.35, we do not observe significant shifts in the lowest energy bleach feature. For the samples with x=0.47-0.57, we observe the bleach feature redshifts at late times (single exciton per dot) compared to early times (multi excitons present). For the high gallium content samples, this indicates when there are multiple excitons in the sample, the additional excitons fill slightly higher energy states compared to the lowest allowed transition.

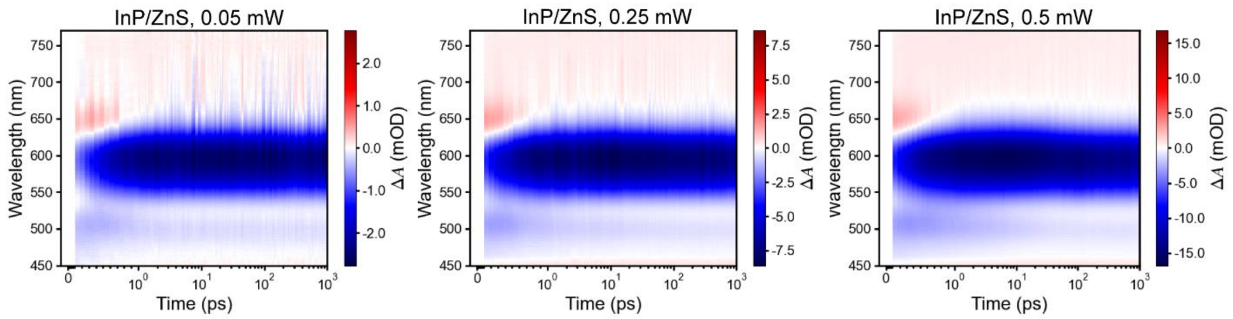

Figure S19. Transient absorption dynamics for 4 nm core diameter InP/ZnS nanocrystals at excitation fluences which produce primarily single excitons (left), single and bi-excitons (middle), and multi-excitons (right).

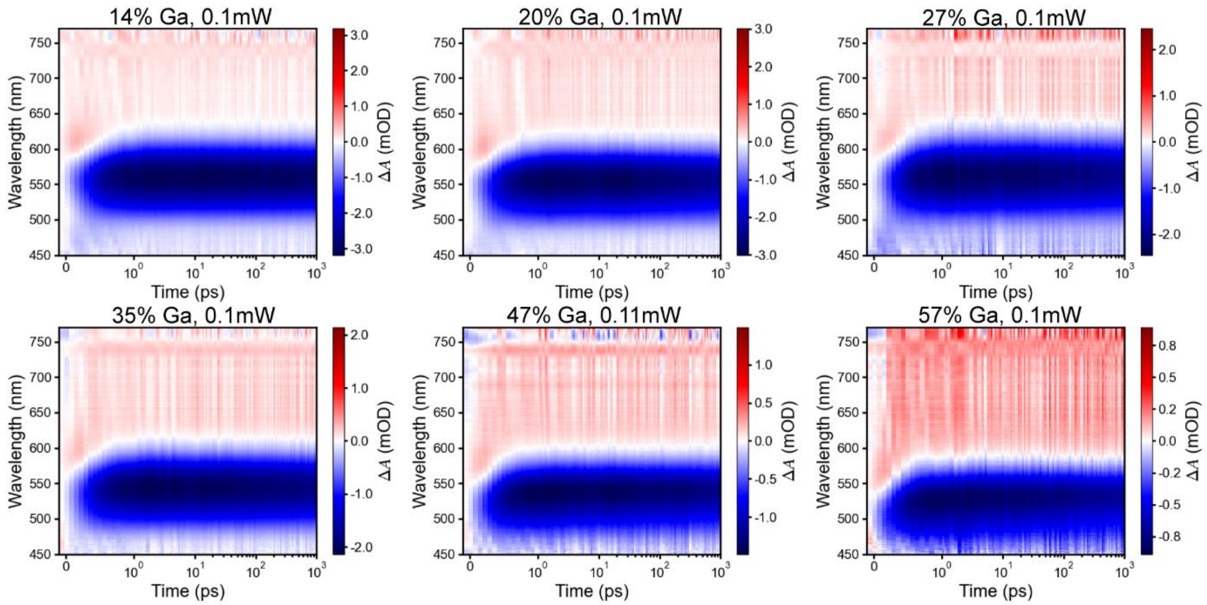

Figure S20. Transient absorption dynamics for single excitons in ~4 nm core diameter $In_{1-x}Ga_xP/ZnS$ nanocrystals with increasing gallium composition.

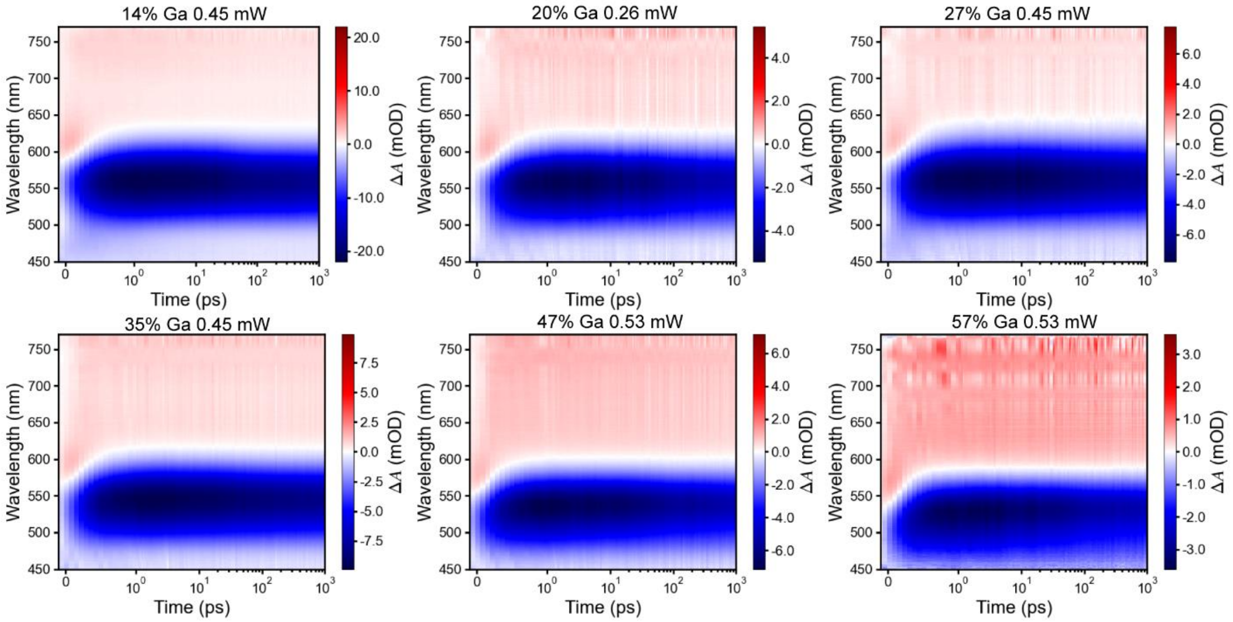

Figure S21. Transient absorption dynamics for single and bi-excitons in ~4 nm core diameter $In_{1-x}Ga_xP/ZnS$ nanocrystals with increasing gallium composition.

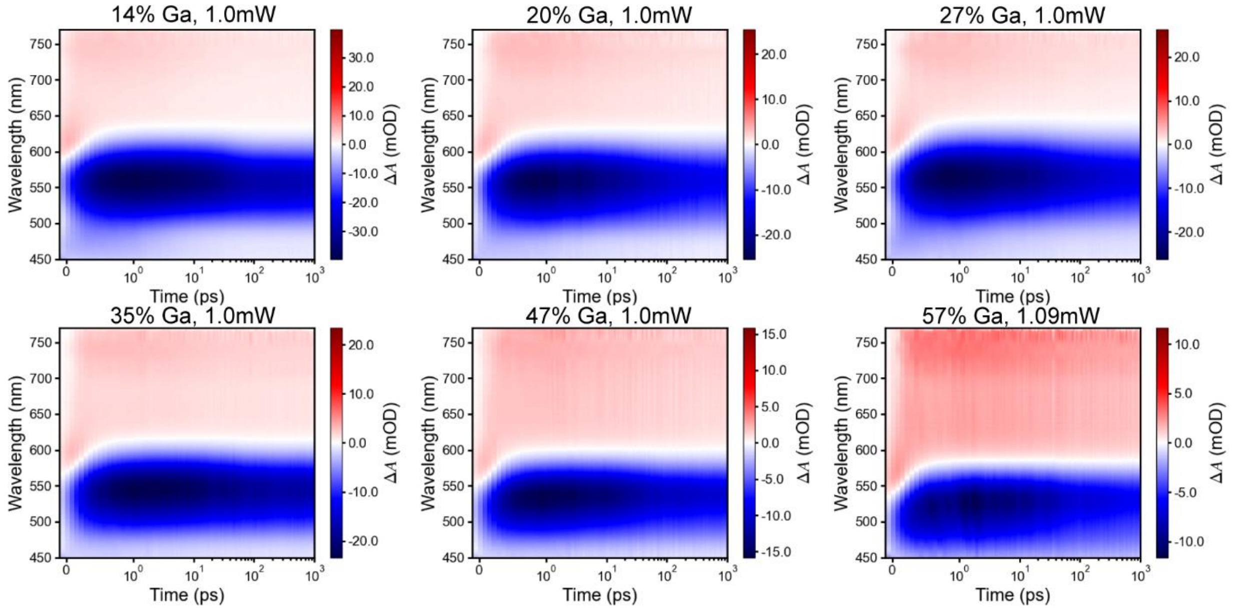

Figure S22. Transient absorption dynamics for multi-excitons in ~4 nm core diameter $In_{1-x}Ga_xP/ZnS$ nanocrystals with increasing gallium composition.

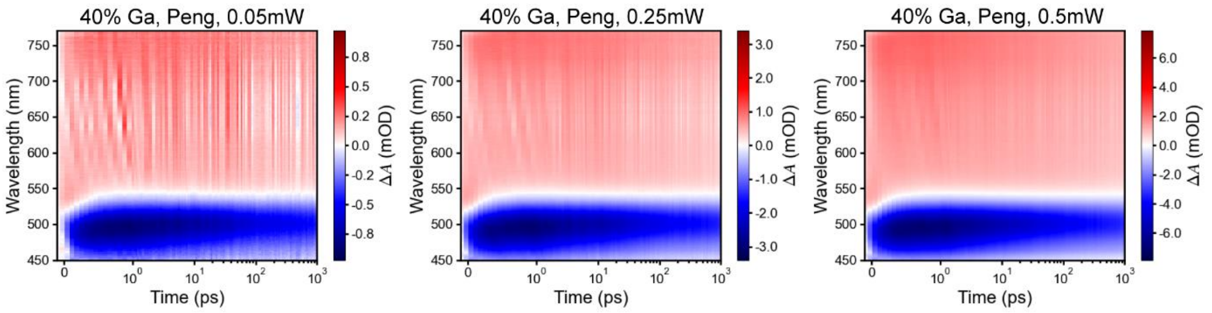

Figure S23. Transient absorption dynamics for ~3.17 nm core diameter $In_{0.60}Ga_{0.40}P/ZnS$ nanocrystals with single (left), bi-(middle) and multi-(right) excitons.

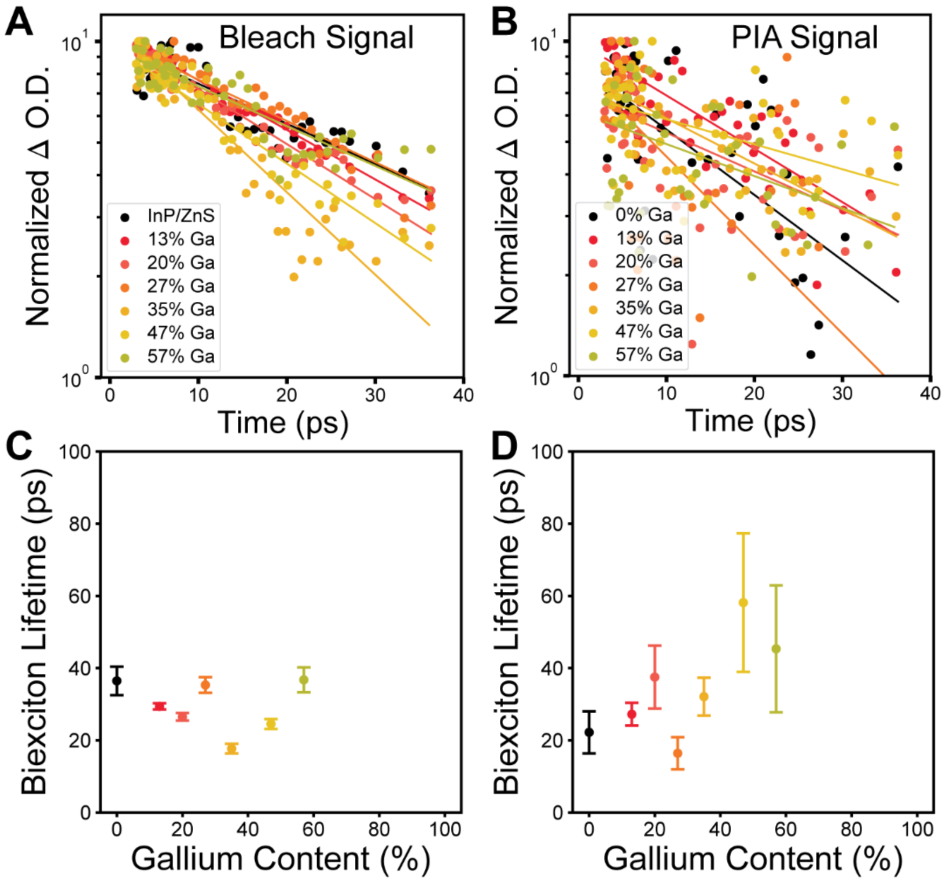

Figure S24. (A) Early time TA bleach signal for ~4 nm diameter core $In_{1-x}Ga_xP/ZnS$ nanocrystals plotted on a semilog scale with corresponding single-exponential fits. **(C)** Extracted biexciton lifetimes from the fits to the early time bleach signal for ~4 nm $In_{1-x}Ga_xP/ZnS$ nanocrystals. **(B)** Early time TA photoinduced absorption signal for ~4 nm diameter core $In_{1-x}Ga_xP/ZnS$

nanocrystals plotted on a semilog scale with corresponding single exponential fits. **(D)** Extracted biexciton lifetime from the fits to the early time photoinduced absorption signal for ~4 nm $In_{1-x}Ga_xP$/ZnS nanocrystals.

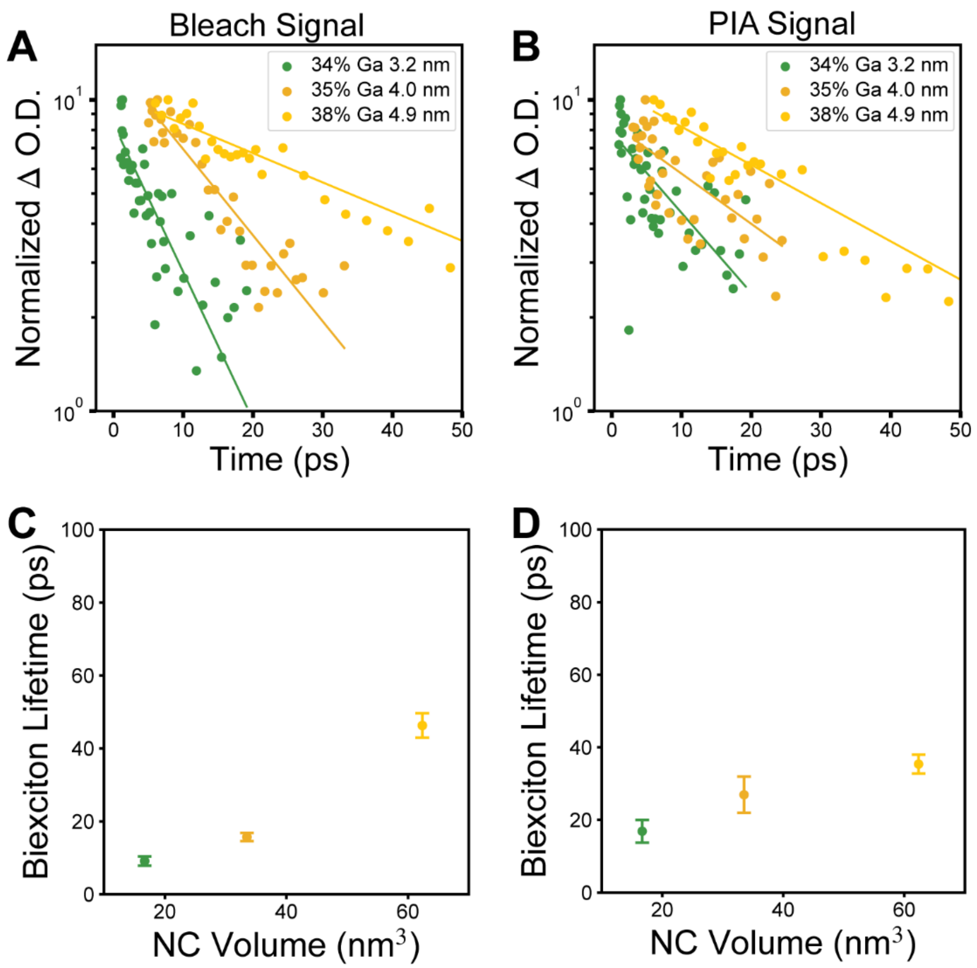

Figure S25. (A) Early time bleach signal for 34-38% Ga containing $In_{1-x}Ga_xP$/ZnS nanocrystals with increasing core diameter plotted on a semilog scale with corresponding single exponential fits. **(C)** Extracted biexciton lifetime versus particle volume using the bleach signal. **(B)** Early time photoinduced absorption signal for 34-38% Ga containing $In_{1-x}Ga_xP$/ZnS nanocrystals with increasing core diameter plotted on a semilog scale with corresponding single exponential fits. **(D)** Extracted biexciton lifetime versus particle volume using the photoinduced absorption signal.

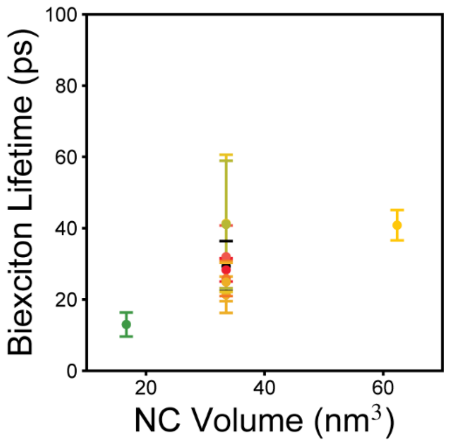

Figure S26. Volume-dependent biexciton lifetime for all $In_{1-x}Ga_xP/ZnS$ samples studied in this manuscript.

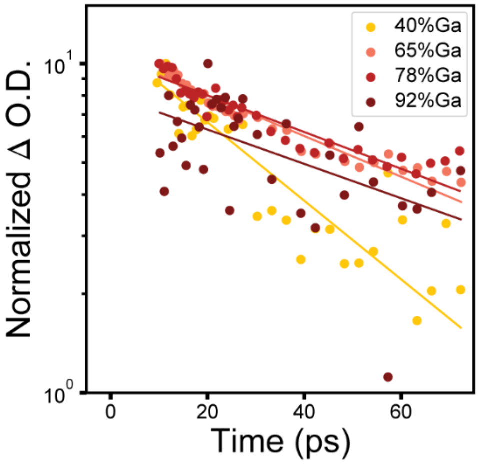

Figure S27. Early time bleach (40% Ga) and photoinduced absorption (65-92% Ga) signal for 4.9 nm diameter $In_{1-x}Ga_xP/ZnS$ nanocrystals with corresponding single-exponential fits. Extracted biexciton lifetimes are presented in Figure 9E.

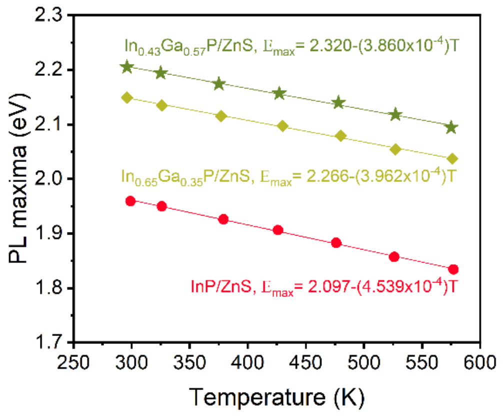

Figure S28. Varshni plots with linear fits exhibit linear trends in the evolution of optical bandgap with temperature in $In_{1-x}Ga_xP$/ZnS samples.

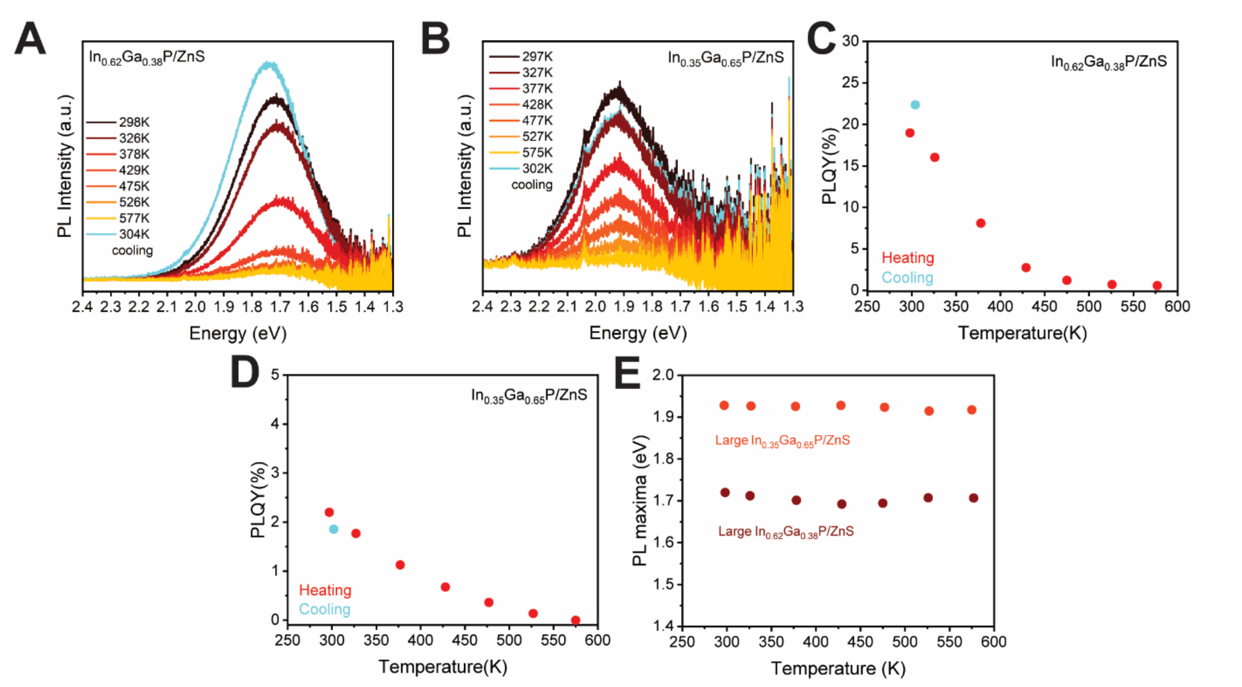

Figure S29. Evolution of PL spectra at higher temperatures in emissive core-shell **(A)** $In_{0.62}Ga_{0.38}P$/ZnS and **(B)** $In_{0.35}Ga_{0.65}P$/ZnS samples derived from the large 4.92 nm InP particles. The trends in PLQY for the same as shown in **(C)** and **(D)** are similar to those of the smaller $In_{1-x}Ga_xP$/ZnS populations described before. Evolution of the bandgap with temperature is described in a Varshni plot in **(E)** and indicates a considerably weaker dependence.

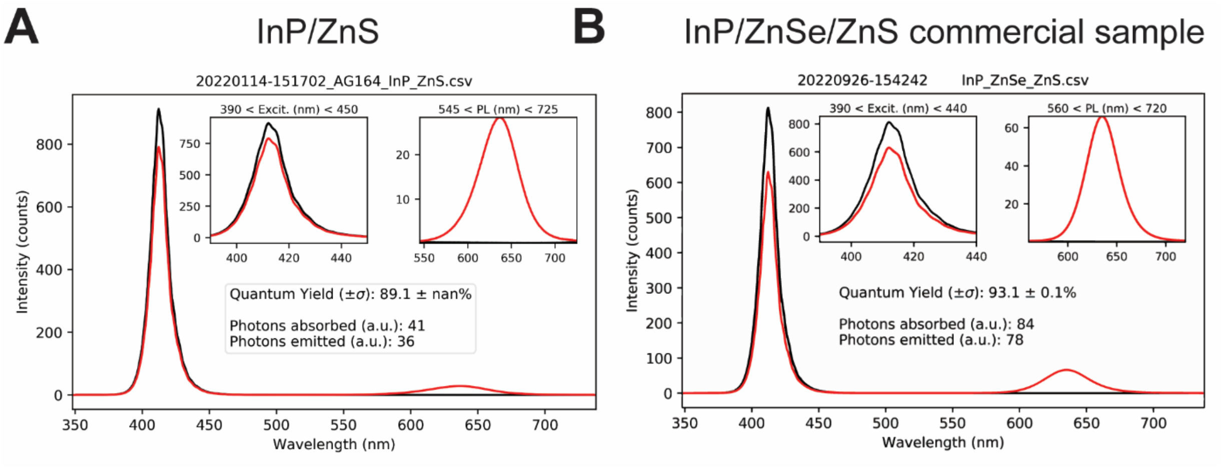

Figure S30. Absolute quantum yield measurements were performed using an integrating sphere setup. **(A)** Room temperature PLQY of the core-shell InP/ZnS sample was estimated at ~89%. **(B)** Room temperature PLQY of the commercial core-shell InP/ZnSe/ZnS sample was estimated at ~93%.

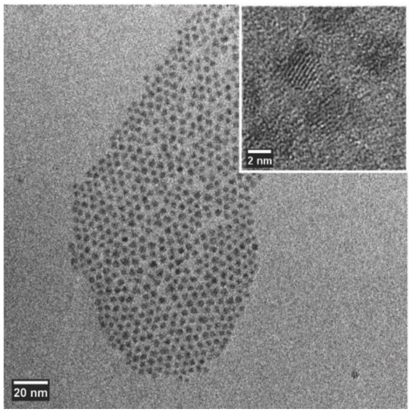

Figure S31. Representative TEM image of the InP/ZnS sample obtained from 4 nm InP nanocrystals shows that only one monolayer of zinc sulfide could be grown on InP under similar conditions. The substantial lattice mismatch likely prevents the shell from growing any thicker.

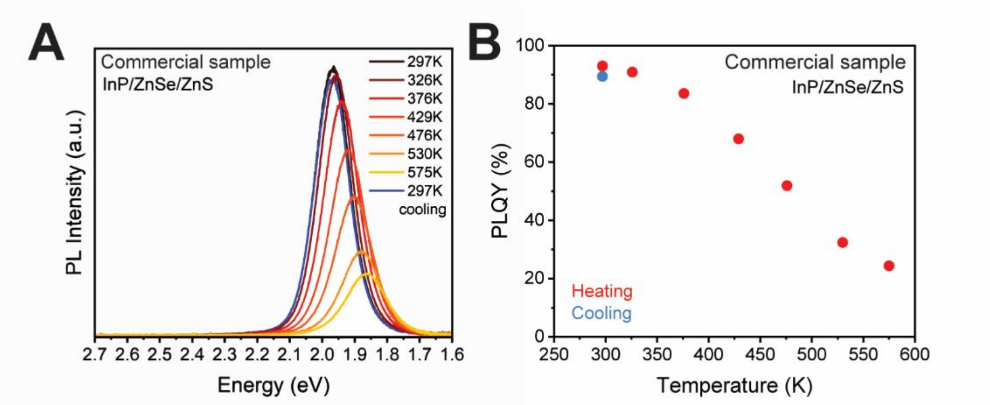

Figure S32. (A) Evolution of PL spectra as a function of elevated temperature in standard emissive core-shell InP/ZnSe/ZnS samples obtained from commercial sources. **(B)** The presence of a thick shell which is better lattice-matched, likely leads to better retention of PLQY at elevated temperatures, as well as near-perfect QY recovery after cooling.

REFERENCES

1. Micic, O. I.; Sprague, J. R.; Curtis, C. J.; Jones, K. M.; Machol, J. L.; Nozik, A. J.; Giessen, H.; Fluegel, B.; Mohs, G.; Peyghambarian, N., Synthesis and Characterization of InP, GaP, and $GaInP_2$ Quantum Dots. *J. Phys. Chem.* **2002,** *99* (19), 7754-7759. DOI: 10.1021/j100019a063.
2. Mićić, O. I.; Nozik, A. J.; Lifshitz, E.; Rajh, T.; Poluektov, O. G.; Thurnauer, M. C., Electron and Hole Adducts Formed in Illuminated InP Colloidal Quantum Dots Studied by Electron Paramagnetic Resonance. *J. Phys. Chem. B* **2002,** *106* (17), 4390-4395. DOI: 10.1021/jp014180q.
3. Li, Y.; Hou, X.; Dai, X.; Yao, Z.; Lv, L.; Jin, Y.; Peng, X., Stoichiometry-Controlled InP-Based Quantum Dots: Synthesis, Photoluminescence, and Electroluminescence. *J. Am. Chem. Soc.* **2019,** *141* (16), 6448-6452. DOI: 10.1021/jacs.8b12908.
4. Gupta, A.; Ondry, J. C.; Chen, M.; Hudson, M. H.; Coropceanu, I.; Sarma, N. A.; Talapin, D. V., Diffusion-Limited Kinetics of Isovalent Cation Exchange in III–V Nanocrystals Dispersed in Molten Salt Reaction Media. *Nano Lett.* **2022,** *22* (16), 6545-6552. DOI: 10.1021/acs.nanolett.2c01699.
5. Yang, Y.; Qin, H.; Jiang, M.; Lin, L.; Fu, T.; Dai, X.; Zhang, Z.; Niu, Y.; Cao, H.; Jin, Y.; Zhao, F.; Peng, X., Entropic Ligands for Nanocrystals: From Unexpected Solution Properties to Outstanding Processability. *Nano Lett.* **2016,** *16* (4), 2133-2138. DOI: 10.1021/acs.nanolett.6b00730.
6. Grabolle, M.; Spieles, M.; Lesnyak, V.; Gaponik, N.; Eychmüller, A.; Resch-Genger, U., Determination of the Fluorescence Quantum Yield of Quantum Dots: Suitable Procedures and Achievable Uncertainties. *Anal. Chem.* **2009,** *81* (15), 6285-6294. DOI: 10.1021/ac900308v.
7. Ilavsky, J.; Jemian, P. R., Irena: Tool Suite for Modeling and Analysis of Small-Angle Scattering. *J. Appl. Crystallogr.* **2009,** *42* (2), 347-353. DOI: doi:10.1107/S0021889809002222.
8. Harris, R. K.; Becker, E. D.; Menezes, S. M. C. d.; Goodfellow, R.; Granger, P., NMR nomenclature. Nuclear spin properties and conventions for chemical shifts(IUPAC Recommendations 2001). *Pure Appl. Chem.* **2001,** *73* (11), 1795-1818. DOI: doi:10.1351/pac200173111795.
9. Hahn, E. L., Spin Echoes. *Phys. Rev.* **1950,** *80* (4), 580-594. DOI: 10.1103/PhysRev.80.580.
10. Fung, B. M.; Khitrin, A. K.; Ermolaev, K., An Improved Broadband Decoupling Sequence for Liquid Crystals and Solids. *J. Magn. Reson.* **2000,** *142* (1), 97-101. DOI: 10.1006/jmre.1999.1896.
11. Metz, G.; Wu, X. L.; Smith, S. O., Ramped-Amplitude Cross Polarization in Magic-Angle-Spinning NMR. *J. Magn. Reson., Ser A* **1994,** *110* (2), 219-227. DOI: 10.1006/jmra.1994.1208.
12. Sakellariou, D.; Lesage, A.; Hodgkinson, P.; Emsley, L., Homonuclear dipolar decoupling in solid-state NMR using continuous phase modulation. *Chem. Phys. Lett.* **2000,** *319* (3), 253-260. DOI: 10.1016/S0009-2614(00)00127-5.
13. Carr, H. Y.; Purcell, E. M., Effects of Diffusion on Free Precession in Nuclear Magnetic Resonance Experiments. *Phys. Rev.* **1954,** *94* (3), 630-638. DOI: 10.1103/PhysRev.94.630.
14. Meiboom, S.; Gill, D., Modified Spin-Echo Method for Measuring Nuclear Relaxation Times. *Rev. Sci. Instrum.* **1958,** *29* (8), 688-691. DOI: 10.1063/1.1716296.
15. Larsen, F. H.; Jakobsen, H. J.; Ellis, P. D.; Nielsen, N. C., Sensitivity-Enhanced Quadrupolar-Echo NMR of Half-Integer Quadrupolar Nuclei. Magnitudes and Relative

Orientation of Chemical Shielding and Quadrupolar Coupling Tensors. *J. Phys. Chem. A* **1997,** *101* (46), 8597-8606. DOI: 10.1021/jp971547b.
16. van Meerten, S. G. J.; Franssen, W. M. J.; Kentgens, A. P. M., ssNake: A cross-platform open-source NMR data processing and fitting application. *J. Magn. Reson.* **2019,** *301*, 56-66. DOI: 10.1016/j.jmr.2019.02.006.
17. Chen, L.; Wang, Q.; Hu, B.; Lafon, O.; Trébosc, J.; Deng, F.; Amoureux, J.-P., Measurement of hetero-nuclear distances using a symmetry-based pulse sequence in solid-state NMR. *Phys. Chem. Chem. Phys.* **2010,** *12* (32), 9395-9405. DOI: 10.1039/B926546E.
18. Lu, X.; Lafon, O.; Trébosc, J.; Amoureux, J.-P., Detailed analysis of the S-RESPDOR solid-state NMR method for inter-nuclear distance measurement between spin-1/2 and quadrupolar nuclei. *J. Magn. Reson.* **2012,** *215*, 34-49. DOI: 10.1016/j.jmr.2011.12.009.
19. Chen, Y.; Smock, S. R.; Flintgruber, A. H.; Perras, F. A.; Brutchey, R. L.; Rossini, A. J., Surface Termination of $CsPbBr_3$ Perovskite Quantum Dots Determined by Solid-State NMR Spectroscopy. *J. Am. Chem. Soc.* **2020,** *142* (13), 6117-6127. DOI: 10.1021/jacs.9b13396.
20. Zhao, X.; Sudmeier, J. L.; Bachovchin, W. W.; Levitt, M. H., Measurement of NH Bond Lengths by Fast Magic-Angle Spinning Solid-State NMR Spectroscopy: A New Method for the Quantification of Hydrogen Bonds. *J. Am. Chem. Soc.* **2001,** *123* (44), 11097-11098. DOI: 10.1021/ja016328p.
21. van Beek, J. D.; Dupree, R.; Levitt, M. H., Symmetry-based recoupling of ^{17}O–^{1}H spin pairs in magic-angle spinning NMR. *J. Magn. Reson.* **2006,** *179* (1), 38-48. DOI: 10.1016/j.jmr.2005.11.003.
22. Brinkmann, A.; Kentgens, A. P. M., Proton-Selective ^{17}O−H Distance Measurements in Fast Magic-Angle-Spinning Solid-State NMR Spectroscopy for the Determination of Hydrogen Bond Lengths. *J. Am. Chem. Soc.* **2006,** *128* (46), 14758-14759. DOI: 10.1021/ja065415k.
23. Bak, M.; Rasmussen, J. T.; Nielsen, N. C., SIMPSON: A General Simulation Program for Solid-State NMR Spectroscopy. *J. Magn. Reson.* **2000,** *147* (2), 296-330. DOI: 10.1006/jmre.2000.2179.
24. Massiot, D.; Fayon, F.; Alonso, B.; Trebosc, J.; Amoureux, J.-P., Chemical bonding differences evidenced from J-coupling in solid state NMR experiments involving quadrupolar nuclei. *J. Magn. Reson.* **2003,** *164* (1), 160-164. DOI: 10.1016/S1090-7807(03)00134-4.
25. Kubo, A.; McDowell, C. A., One- and two-dimensional ^{31}P cross-polarization magic-angle-spinning nuclear magnetic resonance studies on two-spin systems with homonuclear dipolar coupling and J coupling. *J. Chem. Phys.* **1990,** *92* (12), 7156-7170. DOI: 10.1063/1.458255.
26. Wu, G.; Wasylishen, R. E., Homonuclear phosphorus-31 J-resolved 2D spectra of rhodium(I) phosphine complexes in the solid state. *Inorg. Chem.* **1992,** *31* (1), 145-148. DOI: 10.1021/ic00027a030.
27. Chen, Y.; Dorn, R. W.; Hanrahan, M. P.; Wei, L.; Blome-Fernández, R.; Medina-Gonzalez, A. M.; Adamson, M. A. S.; Flintgruber, A. H.; Vela, J.; Rossini, A. J., Revealing the Surface Structure of CdSe Nanocrystals by Dynamic Nuclear Polarization-Enhanced ^{77}Se and ^{113}Cd Solid-State NMR Spectroscopy. *J. Am. Chem. Soc.* **2021,** *143* (23), 8747-8760. DOI: 10.1021/jacs.1c03162.
28. Powell, D.; Migliorato, M. A.; Cullis, A. G., Optimized Tersoff potential parameters for tetrahedrally bonded III-V semiconductors. *Phys. Rev. B* **2007,** *75* (11), 115202. DOI: 10.1103/PhysRevB.75.115202.
29. Wang, L.-W.; Zunger, A., Pseudopotential calculations of nanoscale CdSe quantum dots. *Phys. Rev. B* **1996,** *53* (15), 9579-9582. DOI: 10.1103/PhysRevB.53.9579.

30. Wang, L.-W.; Zunger, A., Local-density-derived semiempirical pseudopotentials. *Phys. Rev. B* **1995,** *51* (24), 17398-17416. DOI: 10.1103/PhysRevB.51.17398.
31. Enright, M. J.; Jasrasaria, D.; Hanchard, M. M.; Needell, D. R.; Phelan, M. E.; Weinberg, D.; McDowell, B. E.; Hsiao, H.-W.; Akbari, H.; Kottwitz, M.; Potter, M. M.; Wong, J.; Zuo, J.-M.; Atwater, H. A.; Rabani, E.; Nuzzo, R. G., Role of Atomic Structure on Exciton Dynamics and Photoluminescence in NIR Emissive InAs/InP/ZnSe Quantum Dots. *J. Phys. Chem. C* **2022,** *126* (17), 7576-7587. DOI: 10.1021/acs.jpcc.2c01499.
32. Rabani, E.; Hetényi, B.; Berne, B. J.; Brus, L. E., Electronic properties of CdSe nanocrystals in the absence and presence of a dielectric medium. *J. Chem. Phys.* **1999,** *110* (11), 5355-5369. DOI: 10.1063/1.478431.
33. Eshet, H.; Grünwald, M.; Rabani, E., The Electronic Structure of CdSe/CdS Core/Shell Seeded Nanorods: Type-I or Quasi-Type-II? *Nano Lett.* **2013,** *13* (12), 5880-5885. DOI: 10.1021/nl402722n.
34. Rohlfing, M.; Louie, S. G., Electron-hole excitations and optical spectra from first principles. *Phys. Rev. B* **2000,** *62* (8), 4927-4944. DOI: 10.1103/PhysRevB.62.4927.
35. Liu, W.; Zheng, W. T.; Jiang, Q., First-principles study of the surface energy and work function of III-V semiconductor compounds. *Phys. Rev. B* **2007,** *75* (23), 235322. DOI: 10.1103/PhysRevB.75.235322.
36. Cohen, M. L.; Bergstresser, T. K., Band Structures and Pseudopotential Form Factors for Fourteen Semiconductors of the Diamond and Zinc-blende Structures. *Phys. Rev.* **1966,** *141* (2), 789-796. DOI: 10.1103/PhysRev.141.789.
37. Li, Y.-H.; Gong, X. G.; Wei, S.-H., Ab initio all-electron calculation of absolute volume deformation potentials of IV-IV, III-V, and II-VI semiconductors: The chemical trends. *Phys. Rev. B* **2006,** *73* (24), 245206. DOI: 10.1103/PhysRevB.73.245206.
38. Jasrasaria, D.; Weinberg, D.; Philbin, J. P.; Rabani, E., Simulations of nonradiative processes in semiconductor nanocrystals. *J. Chem. Phys.* **2022,** *157* (2), 020901. DOI: 10.1063/5.0095897.
39. Benedict, L. X.; Shirley, E. L.; Bohn, R. B., Optical Absorption of Insulators and the Electron-Hole Interaction: An Ab Initio Calculation. *Phys. Rev. Lett.* **1998,** *80* (20), 4514-4517. DOI: 10.1103/PhysRevLett.80.4514.
40. Williamson, A. J.; Zunger, A., Pseudopotential study of electron-hole excitations in colloidal free-standing InAs quantum dots. *Phys. Rev. B* **2000,** *61* (3), 1978-1991. DOI: 10.1103/PhysRevB.61.1978.
41. Fu, H.; Zunger, A., Local-density-derived semiempirical nonlocal pseudopotentials for InP with applications to large quantum dots. *Phys. Rev. B* **1997,** *55* (3), 1642-1653. DOI: 10.1103/PhysRevB.55.1642.
42. Seeger, K., Temperature dependence of the dielectric constants of semi-insulating III-V compounds. *Appl. Phys. Lett.* **1989,** *54* (13), 1268-1269. DOI: 10.1063/1.100735.
43. Klimov, V. I., Spectral and Dynamical Properties of Multiexcitons in Semiconductor Nanocrystals. *Annu. Rev. Phys. Chem.* **2007,** *58* (1), 635-673. DOI: 10.1146/annurev.physchem.58.032806.104537.
44. Calvin, J. J.; Kaufman, T. M.; Sedlak, A. B.; Crook, M. F.; Alivisatos, A. P., Observation of ordered organic capping ligands on semiconducting quantum dots via powder X-ray diffraction. *Nat. Commun.* **2021,** *12* (1), 2663. DOI: 10.1038/s41467-021-22947-x.
